# Tracing the stepwise Darwinian evolution of a plant halogenase

**DOI:** 10.1126/sciadv.adv6898

**Published:** 2025-08-13

**Authors:** Colin Y. Kim, David W. Kastner, Andrew J. Mitchell, Michael A. Gutierrez, Jocelyn S. Yao, Edwin N. Neumann, Heather J. Kulik, Jing-Ke Weng

**Affiliations:** ^1^ Whitehead Institute for Biomedical Research, Cambridge, MA 02142, USA.; ^2^ Department of Biological Engineering, Massachusetts Institute of Technology, Cambridge, MA 02139, USA.; ^3^ Department of Molecular and Cellular Biology, Harvard University, Cambridge, MA 02138, USA.; ^4^ Howard Hughes Medical Institute, Harvard University, Cambridge, MA 02138, USA.; ^5^ Department of Chemical Engineering, Massachusetts Institute of Technology, Cambridge, MA 02139, USA.; ^6^ Department of Chemistry, Massachusetts Institute of Technology, Cambridge, MA 02139, USA.; ^7^ Institute for Plant-Human Interface, Northeastern University, Boston, MA 02120, USA.; ^8^ Departments of Chemistry & Chemical Biology, Bioengineering, and Chemical Engineering, Northeastern University, Boston, MA 02120, USA.

## Abstract

Biohalogenation is rare in plant metabolism, with the Menispermaceae’s chloroalkaloid acutumine being an exception. This involves a specialized dechloroacutumine halogenase (DAH) from the iron- and 2-oxoglutarate–dependent dioxygenase (2ODD) family. While DAH is presumed to have evolved from an ancestral 2ODD, how enzyme specialization arises through Darwinian processes remains a fundamental question in understanding metabolic evolution. Here, we investigate the evolutionary history of DAH using the chromosomal-level genome of *Menispermum canadense*. Phylogenomic dating and synteny analyses reveal DAH evolution through tandem duplication of an ancestral flavonol synthase (FLS) gene, followed by neofunctionalization and gene loss events. Structural modeling, molecular dynamics, and site-directed mutagenesis identify mutations enabling the catalytic switch from FLS to DAH. This required traversing a complex evolutionary landscape with deep fitness valleys separating intermediate states captured in the *M. canadense* genome. Our findings illustrate how enzymatic functions evolve through lineage-specific pathways, reshaping active sites and enabling catalytic mechanism-switching mutations.

## Teaser

Moonseed evolved rare chloroalkaloids via stepwise mutations that rewire an ancestral oxidative enzyme into a halogenase.

## INTRODUCTION

Halogenation in nature is a valuable chemical transformation that enhances the diversity and functionality of natural products, contributing to their medicinal potency (*1*–*3*). While plants have developed elaborate specialized metabolic pathways to produce a dazzling array of structurally diverse secondary compounds, halogenation chemistry is rarely observed. To date, dechloroacutumine halogenase (DAH), which catalyzes the terminal chlorination in acutumine biosynthesis in the Menispermaceae family, stands out as the only characterized halogenase across all land plants (*4*). The limited occurrence of halogenated plant natural products presents an opportunity to expand nature’s chemical space through biocatalytic halogenation, potentially yielding new pharmaceutically relevant compounds. However, with DAH being the only characterized plant halogenase, understanding how these enzymes evolved remains a key challenge for rationally designing new halogenases. DAH thus offers a unique window into understanding how nature can evolve new catalytic functions through Darwinian processes.

DAH is an iron(II)- and 2-oxoglutarate [Fe(II)/2OG]–dependent halogenase (2ODH) of plant origin, belonging to the large superfamily of iron(II)- and 2-oxoglutarate–dependent dioxygenases (2ODDs) (*4*). 2ODDs are found across all kingdoms of life that use a nonheme ferrous cofactor for their radical oxidative catalysis (*5*). These enzymes target otherwise inactive sp^3^ and sp^2^ C─H bonds at a highly conserved iron-binding facial triad (HxD/EX*_n_*H) in their active site for a diverse range of reactivities, with hydroxylation being by far the most common (*6*). 2ODHs are evolutionarily derived from 2ODDs and harbor a mechanistic active-site substitution, replacing the key acidic residue Asp/Glu of the facial triad in 2ODDs with a Gly/Ala (*7*). This allows the halide ligand to occupy an iron coordination site, and in turn elicits halogenation chemistry (*8*). While the key mechanistic D-to-G mutation is only observed once in plants to yield DAH, a handful of 2ODHs have been characterized in bacteria including WelO5 from welwitindolinone biosynthesis in *Hapalosiphon welwischii* (*8*) and a group of amino acid halogenases from *Streptomyces cattleya* (*9*). Leveraging this highly conserved mechanism along with diverse reaction outcomes, 2ODD has been recognized as a promising biocatalytic scaffold for directed evolution over the past decade to engineer regio-stereospecific halogenases and azidases (*10*–*12*). In particular, a bacterial Fe(II)/2OG hydroxylase, MBT76 in lysine metabolism of *Streptomyces*, was converted to a lysine halogenase inspired by sequence and structural comparison between a pair of closely related hydroxylase and halogenase (*13*). While activity-guided directed evolution of Fe(II)/2OG hydroxylases has been explored, the natural evolutionary pathway leading to 2ODHs has not been studied in any host organism.

Plant genomes, with their large size and frequent duplication events, serve as rich but largely untapped reservoirs of evolutionary history, preserving the genomic footprints of how biosynthetic genes acquire specialized functions to expand metabolic diversity (*14*). For example, the metabolic diversity of acylsugars found in cultivated tomato roots is driven by gene duplication followed by neofunctionalization within a conserved biosynthetic gene cluster across various cultivated Solanaceae genomes (*15*). As the only known 2ODH in plant metabolism, DAH’s unique emergence in Menispermaceae provides a valuable opportunity to investigate the molecular mechanisms underlying its evolution, with potential implications for designing biocatalysts for targeted C─H functionalization. Here, we report a chromosomal-level genome assembly of *Menispermum canadense* and trace DAH’s evolution from its progenitor flavonol synthase (FLS), a Fe(II)/2OG desaturase, through two evolutionary intermediate pseudogenes preserved in the genome. Using structural models of DAH and FLS, we identify a set of amino acid substitutions and insertions necessary for the functional transition from FLS to DAH. Through mechanism-guided engineering of additional selected plant 2ODDs, we validate the rugged evolutionary landscape leading to DAH, which in turn highlights the role of lineage-specific evolutionary events in enabling subsequent mechanism-switching mutations in enzyme evolution.

## RESULTS

### Chromosomal-level assembly of the *M. canadense* genome

**Fig. 1.**
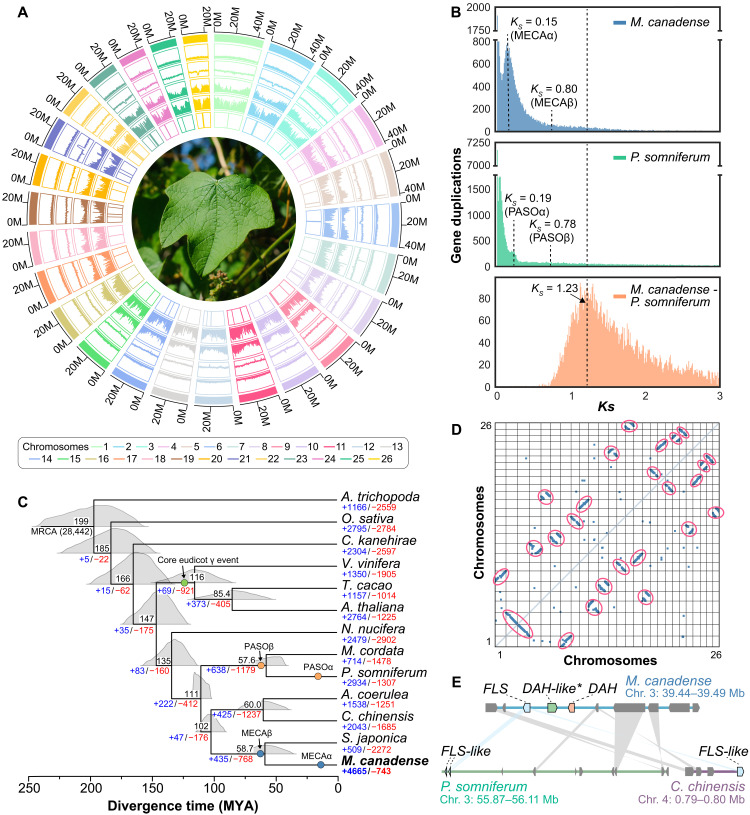
Chromosomal-level genome assembly of *M. canadense*. (**A**) Circos plot summarizing genomic features for each pseudochromosome, including chromosome size, repeat element density, GC content, gene density, transcript coverage, and assembly gaps (from outer to inner tracks). A photo of *M. canadense* is shown in the center. Image credit: C.Y.K. (**B**) *K_S_* distribution based on all-to-all blast of total orthologous gene pairs in *M. canadense* and *P. somniferum* reveals WGDs. Mixtools analysis identified *K_S_* peaks at 0.15 (MECAα) and 0.80 (MECAβ) in *M. canadense*, 0.19 (PASOα) and 0.78 (PASOβ) in *P. somniferum*, and 1.23 for their divergence. (**C**) Phylogenomic divergence time of *M. canadense* compared to 12 other angiosperm species; *Amborella trichopoda*, *Oryza sativa*, *Cinnamomum kanehirae*, *Vitis vinifera*, *Theobroma cacao*, *Arabidopsis thaliana*, *Nelumbo nucifera*, *M. cordata*, *P. somniferum*, *A. coerulea*, *C. chinensis*, and *S. japonica*. Black numbers indicate estimated divergence times (MYA); blue and red numbers indicate gene family expansions and contractions, respectively. The most recent common ancestor (MRCA) is inferred to have 28,442 gene families. Green circle indicates the relative timing of the core eudicot γ event; orange circles correspond to WGDs in *P. somniferum*; blue circles indicate WGDs in *M. canadense*. (**D**) Dot plot of intragenomic synteny within the *M. canadense* showing a total of 10,745 duplicated genomic pairs with a C-score cutoff of 0.90. Red circles highlight WGD-derived pairs. (**E**) Microsynteny comparison of DAH-containing loci in *M. canadense* (Chr. 3; 39.44 to 39.49 Mb) and *P. somniferum* (Chr. 3; 55.87 to 56.11 Mb) or *C. chinensis* (Chr. 4; 0.79 to 0.80 Mb).

To shed light on the evolutionary history of *DAH*, we sequenced and assembled the genome of *M. canadense*. This species is diploid (2*n* = 2*x* = 52), with an estimated genome size of approximately 958 Mb according to *k*-mer (*k* = 19) analysis (fig. S1) and 925 Mb according to flow cytometry (fig. S2). We extracted high-molecular weight genomic DNA from leaf tissues and performed highly accurate long-read sequencing (Pacific Biosciences). The initial draft assembly using HiFi circular consensus sequencing reads yielded a size of approximately 970 Mb with 1327 contigs and an area under the N*x*-curve (auN) of 15.6 Mb (fig. S3 and table S1). To improve the assembly, we used the high-throughput chromosome conformation capture (Hi-C) technique to scaffold 890.5 Mb of the assembly onto 26 pseudochromosomes with an auN of 34.2 Mb, covering 96.28% of the expected genome size from flow cytometry (Fig. 1A, figs. S4 and S5, and table S2). We mapped Illumina short-read sequencing data onto this reference genome, resulting in 98.87% of the 1.821 billion total reads being mapped onto 26 pseudochromosomes (table S3).

On the basis of this assembled *M. canadense* genome, we annotated 65,843 protein-coding genes with homologous alignments, ab initio gene models, and transcriptome data from our previous study (*4*). In total, 99.9% of the transcripts longer than 1000 base pair could be mapped onto the *M. canadense* genome with >50% sequence coverage (table S4). To assess its completeness, the Benchmarking Universal Single-Copy Orthologs (BUSCO) (*16*) analysis was conducted, which demonstrated a score of 99.4% (1604 of 1614 conserved genes) (fig. S6). In addition, the OMArk (*17*) analysis for its proteome revealed its high completeness with a score of 96.37% (7832 of 8127 conserved hierarchical orthologous groups) (fig. S7). Moreover, Merqury (*18*) analysis of the Hi-C scaffolded assembly showed a completeness score of 83.97% with the quality value of 59.66 and low error rate of 1.08 × 10^−6^ (table S5). The final assembly statistics are shown in a Circos plot (Fig. 1A). The highly contiguous and complete genome of *M. canadense* enables us to leverage bioinformatic analyses to investigate the genomic distribution and evolution of enzymes involved in acutumine-type alkaloid biosynthesis.

### Phylogenomic analysis of the *M. canadense* genome

To enable comparative genomic study of *M. canadense* relative to other plants with sequenced genomes, we first used OrthoFinder (*19*) to obtain orthologous groups between *M. canadense* and 12 other angiosperm species (table S6). Of 55,164 curated genes from the *M. canadense* genome, 47,394 (85.9%) genes were placed in orthologous groups and 15,244 (27.6%) in *M. canadense*–specific groups (table S7). Using 249 single-copy orthologous sequences across all 13 species, we constructed a maximum-likelihood phylogenetic tree. The coalescence-based analysis suggests that *M. canadense* shares a recent common ancestor with *Stephania japonica*, a Menispermaceae plant that does not produce acutumine (Fig. 1C). Menispermaceae plants are most closely related to the Ranunculaceae family, which includes *Coptis chinensis* and *Aquilegia coerulea*, while the Papaveraceae family, containing *Papaver somniferum* and *Macleaya cordata*, is the immediate outgroup (Fig. 1C). *M. canadense* belongs to the Ranunculales, a basal eudicot group evolved in parallel to the core eudicots (Fig. 1C). Using MCMCtree (*20*) with molecular clock calibrations, we estimated that eudicots diverged from early angiosperms ~166 million years ago (MYA), with basal eudicots and core eudicots diverging ~19 MYA afterward. Within Ranunculales, Papaveraceae species diverged ~111 MYA, Menispermaceae species diverged from Ranunculaceae ~103 MYA, and *M. canadense* diverged from *S. japonica* ~59 MYA (Fig. 1C). Analysis of gene family evolution revealed 5507 expanded and 2518 contracted families in *M. canadense*, compared to 1351 expanded and 4047 contracted in *S. japonica*, 2875 expanded and 3929 contracted in *C. chinensis*, and 3932 expanded and 3317 contracted in *P. somniferum* (Fig. 1C). Among 158 gene families annotated as 2ODD, we observed 47 expansions and 22 contractions in *M. canadense* (fig. S8).

### WGD events indicated by the *M. canadense* genome

Ancient whole-genome duplication (WGD) provides a primary mechanism for generating copy number variation of biosynthetic genes in plants and can be detected through analysis of paralogous gene age distributions (*21*, *22*). Given the large gene family expansions in *M. canadense*, we investigated whether Menispermaceae-family plants had undergone lineage-specific WGD events that might have contributed to the emergence of *DAH*. We analyzed potential WGD events by calculating the number of synonymous mutations per synonymous site (*K*_S_) for paralogous genes (*22*, *23*), followed by peak detection using a mixture model from the mixtools R package (*24*). The distribution of reciprocal best hit paralogous gene pair *K*_S_ values exhibits peaks at 0.80 (41.7 MYA) in *M. canadense*, 0.89 (46.3 MYA) in *S. japonica*, 0.78 (40.6 MYA) in *P. somniferum*, and 0.72 (37.5 MYA) in *C. chinensis*, suggesting ancient WGD events (Fig. 1B and fig. S9). This WGD event, designated as MECAβ, occurred after the divergence of *M. canadense* from *P. somniferum* (*K*_S_ = 1.23; 64.1 MYA) and *C. chinensis* (*K*_S_ = 1.10; 57.3 MYA), but before its divergence from *S. japonica* (*K*_S_ = 0.7; 36.5 MYA) (Fig. 1B and fig. S9). This indicates that an ancient WGD event occurred in the evolutionary history of *M. canadense* after Menispermaceae split from other Ranunculales plants, independent of other Ranunculales WGD events. This WGD event is evident in the syntenic dotplot of the *M. canadense* genome using paralogous gene pairs (Fig. 1D and fig. S10A).

To examine additional rounds of ancient WGD, we compared syntenic depth ratios between *M. canadense* and *Amborella trichopoda*. We observed a four-to-one syntenic depth ratio, indicating that a single *A. trichopoda* genomic region aligns to four *M. canadense* blocks (fig. S10B). Since *A. trichopoda* has not experienced any WGD after the ancestral angiosperm genome duplication event (*25*), this ratio suggests that *M. canadense* underwent two rounds of ancient WGD since their last common ancestor. Furthermore, we observed two-to-one and four-to-four syntenic depth ratios between *M. canadense* and *C. chinensis* and *P. somniferum*, respectively (fig. S10, C to E). Previous analyses show that *C. chinensis* experienced one WGD event (*K*_S_ = 0.72; 37.5 MYA), while *P. somniferum* underwent both a Papaveraceae-specific WGD (PASOβ; *K*_S_ = 0.78; 40.6 MYA) and an additional *Papaver*-specific WGD (*26*) (PASOα; *K*_S_ = 0.19; 9.9 MYA) (Fig. 1B and figs. S9 and S10). Similarly, *M. canadense* experienced an additional WGD event (MECAα; *K*_S_ = 0.15; 7.8 MYA) after the Menispermaceae-specific WGD (MECAβ; *K*_S_ = 0.80; 41.7 MYA) (Fig. 1B and fig. S9). The two-to-one syntenic depth ratio between *M. canadense* and *S. japonica* and two-to-two ratio between *S. japonica* and *C. chinensis* (*27*) support that MECAβ is shared within Menispermaceae while MECAα is specific to the *Menispermum* genus (fig. S10, G and H).

### Genomic signatures of *DAH* evolution in the *M. canadense* genome

**Fig. 2.**
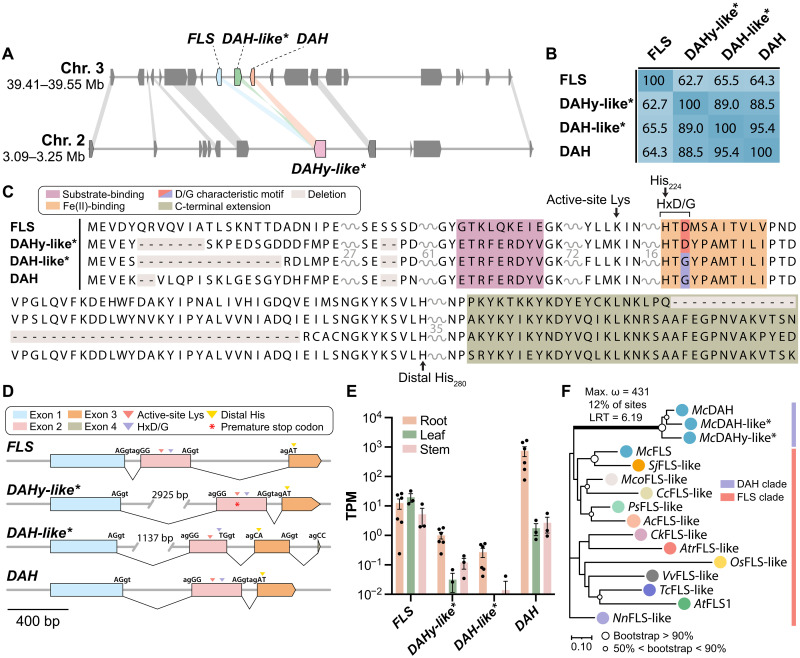
Synteny-based analysis of *DAH* locus and its evolutionary context. (**A**) WGD region on chromosomes 3 and 2 containing *FLS*, *DAH*, and its paralogous genes. Asterisk denotes pseudogenes. (**B**) Amino acid sequence identity matrix of FLS, DAHy-like, DAH-like, and DAH calculated by local pairwise alignments using EMBOSS supermatcher (*105*). Asterisk denotes nonfunctional proteins. (**C**) MSA of FLS, DAHy-like, DAH-like, and DAH found on chromosomes 2 and 3. Asterisk denotes nonfunctional proteins. (**D**) Intron-exon structure of *FLS*, *DAHy-like*, *DAH-like*, and *DAH*. All gene structures are shown in 5′-3′ orientation. All genes share the first two intron-exon splice junction sequences. Asterisk denotes pseudogenes. (**E**) TPM values of tissue specific RNA-seq reads of *M. canadense* mapped onto *FLS*, *DAHy-like*, *DAH-like*, and *DAH*. TPM values are shown at log_10_ scale. Data are presented as mean values ± SEM with six biological replicates of root tissues, three of leaf and three of stem. (**F**) Maximum-likelihood phylogenetic tree of DAH, its paralogs and FLS orthologs from select angiosperm species represented in Fig. 1C. Bootstrap statistics (200 replicates) are indicated at the tree nodes. The scale measures evolutionary distance in substitutions per amino acid. The branch leading to the common ancestor of DAH and FLS recovered with significant (*P* = 0.0161) evidence of positive selection, with 12% of sites showing strong directional selection (ω or max d_N_/d_S_ = 431) according to the aBSREL method. Asterisk denotes nonfunctional proteins.

To probe the evolutionary origin of *DAH*, we examined the genomic loci containing *DAH* and its closely related homologs in the *M. canadense* genome. We found that the *DAH* gene, located on chromosome 3, is proximal to two paralogous genes: *DAH-like*, positioned next to *DAH*, contains the halogenase sequence motif HxGX*_n_*H and shares 95.4% amino acid sequence identity with DAH; and *FLS*, positioned next to *DAH-like*, contains the hydroxylase sequence motif HxDX*_n_*H and shares 64.3% amino acid sequence identity with DAH (Fig. 2, A to C). Syntenic analysis revealed corresponding regions in *P. somniferum* and *C. chinensis* genomes, containing tandemly duplicated *FLS-like* genes in *P. somniferum* and a single *FLS-like* gene in *C. chinensis*, but no *DAH-like* genes in either case (Fig. 1E). In addition, the WGD event (MECAα) within the *M. canadense* genome produced a duplicated region of this locus on chromosome 2 (Fig. 2A). Unlike the tandem arrangement on chromosome 3, this duplicated region contains only one gene, named *dechloroacutumine hydroxylase–like* (*DAHy-like*), which contains the hydroxylase sequence motif HxDX*_n_*H and shares 88.5% amino acid sequence identity with DAH (Fig. 2, A to C). Similarly, WGD (PASOα) within the *P. somniferum* genome duplicated the *FLS-like* gene locus, resulting in a total of four *FLS-like* genes (fig. S11).

To investigate the evolutionary relationship among *DAH*, *DAH-like*, *DAHy-like*, and *FLS* in *M. canadense*, we examined their exon-intron structures and found that the splicing junction sequences between *FLS*, *DAHy-like*, and *DAH* are conserved (Fig. 2D). For *DAH-like*, only the splicing junction sequences for the first intron are conserved. At the protein level, multiple sequence alignment (MSA) shows that DAH, DAH-like, and DAHy-like all contain a 13–amino acid C-terminal extension compared to FLS, while DAH-like has a 30–amino acid internal truncation compared to DAH in a region likely critical for protein folding and catalysis, suggesting a loss of function (Fig. 2C). This truncation is found in the second exon of *DAH-like*, which contains unique splicing junction sequences for its second intron and a unique third exon (Fig. 2D and figs. S12 and S13). Both *DAHy-like* and *DAH-like* genes have elongated first introns compared to *FLS* and *DAH*, suggesting enhanced potential for regulation and increased transcriptional cost as a result of gene duplication (Fig. 2D) (*28*, *29*). Moreover, analysis of tissue-specific transcriptome datasets from our previous study (*4*) revealed that *DAHy-like* and *DAH-like* exhibited generally lower transcript-per-million values than *FLS* and *DAH* across all three tissues (Fig. 2E). Mapped RNA sequencing (RNA-seq) reads for *DAHy-like* indicate that an independent *M. canadense* sample carries a naturally occurring allele with two single-nucleotide polymorphisms at the Lys^200^ codon (“AAA” to “TAG”), resulting in a premature stop codon (fig. S14). While this suggests the possibility of relaxed selection or early pseudogenization, we interpret *DAHy-like* and *DAH-like* as potential remnants of evolutionary intermediates in the trajectory linking the *FLS* progenitor to *DAH*. The low expression levels, large truncation in *DAH-like*, and natural presence of an allele containing a premature stop codon in *DAHy-like* suggest that these two *DAH* paralogs are likely no longer functional and are undergoing pseudogenization in the *M. canadense* genome.

To further resolve the phylogenetic relationships between FLS and the three DAH paralogs, we constructed a maximum-likelihood phylogenetic tree using DAH, DAH-like, DAHy-like, and representative FLS orthologs from the orthologous groups generated in *M. canadense* and 11 relevant angiosperm species (Fig. 2F). DAH, DAH-like, and DAHy-like appear to form a distinct clade separate from the FLS clade. Moreover, the branching pattern shows that proteins with the acquired halogenase sequence motif HxGX*_n_*H (i.e., DAH and DAH-like; His^244^ as the first histidine, His^280^ as the second, distal histidine) were likely derived from an ancestral DAHy-like protein, which harbored the hydroxylase motif HxDX*_n_*H. Overall, these phylogenetic analyses suggest that the characteristic D-to-G mechanism-switching mutation observed in DAH and DAH-like occurred in an evolutionary intermediate paralogous to DAHy-like after it had diverged from FLS. In addition, FLS homologs can be found in angiosperms ranging from early monocots to core eudicots (Fig. 2F). This indicates that flavonol and flavonoid biosynthesis are highly conserved across these species, yet the divergence toward DAH appears limited to *Menispermum* plants alone. To further test for positive selection of DAH, we performed a targeted molecular adaptation analysis using the coding nucleotide sequences of the genes from this phylogenetic tree for the adaptive branch-site random effects likelihood test for episodic diversification (aBSREL). Our analysis revealed that 12% of the nucleotide coding sequence sites along the branch leading to the DAH clade containing *McDAH*, *McDAH-like*, and *McDAHy-like* exhibited a statistically significant signal of episodic positive selection, with *d*_N_/*d*_S_ > 1 (ω or max *d*_N_/*d*_S_ = 431) (Fig. 2F). This finding suggests a signal of neofunctionalization, where DAH has acquired distinct functional capabilities from its ancestral FLS role.

### Structural basis for the evolution of DAH from an FLS progenitor

**Fig. 3.**
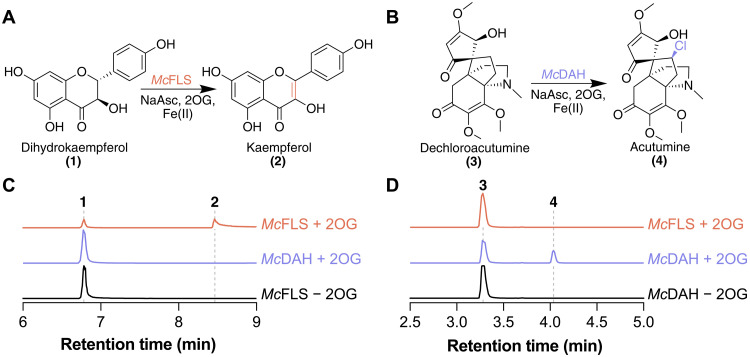
In vitro biochemical assay of FLS and DAH. (**A**) Reaction schematic of the conversion of dihydrokaempferol; **1** to kaempferol; **2** using flavonol synthase (*Mc*FLS). (**B**) Reaction schematic of the conversion of dechloroacutumine; **3** to acutumine; **4** using dechloroacutumine halogenase (*Mc*DAH). (**C**) Combined liquid chromatography–mass spectrometry (LC-MS) extracted ion chromatograms (EICs) of 287.05594 *m/z*; **1** = [M-H]^−^ and 285.04050 *m/z*; **2** = [M-H]^−^. EICs show the in vitro activity of *Mc*FLS that desaturate **1** to **2** in a 2OG-dependent manner. *Mc*DAH produces trace amounts of **2** in the reaction (fig. S17). (**D**) Combined LC-MS EICs of 364.17526 *m/z*; **3** = [M + H]^+^ and 398.13629 *m/z*; **4** = [M + H]^+^. EICs show the in vitro activity of *Mc*DAH that chlorinate **3** to **4** in a 2OG-dependent manner, while *Mc*FLS exhibits no production of **4**.

As genomic signatures in *M. canadense* suggest that chlorinated alkaloids in plants evolved from flavonol biosynthesis, we next sought to examine possible mutational trajectories and the structural basis underlying the FLS-to-DAH functional transition. We first characterized the biochemical functions of FLS and DAH using purified recombinant proteins against their native substrates in in vitro assays (fig. S15). FLS catalyzes desaturation of dihydroflavonols, dihydrokaempferol, and dihydroquercetin to produce flavonols, kaempferol, and quercetin, respectively (Fig. 3, A and C, and fig. S16). Unexpectedly, a trace amount of kaempferol was also detected when DAH was assayed against dihydrokaempferol, suggesting that it still retains a low level of the ancestral FLS activity (fig. S17). Likewise, we examined whether FLS harbors any reactivity against dechloroacutumine. While DAH shows 2OG-dependent chlorinase activity on dechloroacutumine to produce acutumine, FLS does not exhibit any oxidase or halogenase activity on dechloroacutumine (Fig. 3, B and D). Furthermore, the expression of pseudogenes encoding evolutionary intermediates, DAH-like and DAHy-like, resulted in insoluble protein during the purification procedure, consistent with the observation of pseudogenization.

**Fig. 4.**
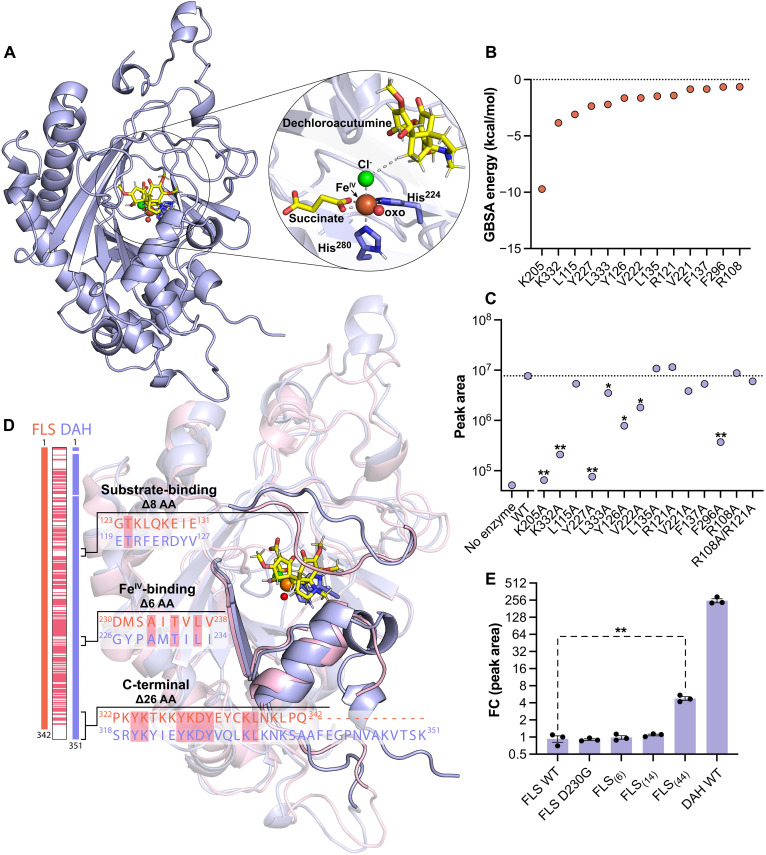
Structural model–guided characterization and molecular evolution of DAH. (**A**) QM/MM–optimized structural model of *Mc*DAH generated with constraints that favor an obtuse oxo-Fe(IV)-H target angle. Predicted active site residues are shown in purple sticks, along with optimized positioning of dechloroacutumine, succinate, Cl anion, and Fe(IV)-oxo species. (**B**) MM-GBSA hydrogen-bonding energy calculations from MD simulations of *Mc*DAH structural model. Fourteen lowest energy residues with their GBSA energy values are shown. (**C**) Catalytic activity of *Mc*DAH alanine mutants. Target mutants were inferred from GBSA energy decomposition analysis and the structural model of *Mc*DAH. LC-HRAM-MS peak areas of acutumine ([M + H]^+^ = 398.13629 *m/z*) are shown for all samples. Dashed line represents the average peak area for acutumine detected using *Mc*DAH WT. All assays were performed in triplicates and the error bars represent SEM. Single asterisk (*) indicates *P* < 0.01 and double asterisk (**) indicates *P* < 0.001 compared to the WT peak area of acutumine. (**D**) Sequence and structural alignment of *Mc*DAH and *Mc*FLS structural models. Both structures have been QM/MM optimized starting from centroids of MD simulations. (**E**) Structural model–guided design of FLS mutants for minimal halogenase activity. Fold-change (shown in log_2_-scale) between the LC-HRAM-MS peak areas of acutumine ([M + H]^+^ = 398.13629 *m/z*) in all samples and that of FLS WT sample (negative control) are shown. All assays were performed in triplicates with normalized enzyme concentration at 5 μM, and the error bars represent SEM. Double asterisk (**) indicates *P* < 0.001 compared to the normalized fold-change peak area of the FLS WT sample.

To examine the structural features contributing to the functional divergence between FLS and DAH, we obtained structural models of DAH and FLS using AlphaFold2 (*30*), followed by docking of dechloroacutumine and dihydrokaempferol into their active sites, respectively (fig. S18). We estimated the positioning of Fe(II), Cl^−^ anion, and 2OG based on a structural alignment with a previously reported crystal structure of the *Arabidopsis thaliana* anthocyanidin synthase (*At*ANS; PDB: 2BRT) (fig. S19) (*31*). From the structural alignment, we noticed that the characteristic D-to-G mutation in DAH creates two open coordination positions, axial or equatorial to His^224^, for the oxo/hydroxo ligand of the Fe(IV)═O/Fe(II)-OH unit to occupy (fig. S20). Thus, we derived both axial-oxo and equatorial-oxo conformational models to represent the reactive state of DAH with Fe(IV)-oxo and succinate (fig. S20). We then ran extended molecular dynamics (MD) simulations of both DAH isomers with constraints favoring either an acute or obtuse oxo-Fe(IV)-H target angle (figs. S20 to S23). Previous examination of available crystallographic and spectroscopic data revealed that halogenases prefer obtuse angles and hydroxylases prefer acute angles (*32*). When analyzing the angle and distance preferences of the spectroscopically guided MD simulations, we noticed that FLS simulations preferred the acute conformation, while DAH preferred the obtuse conformation (figs. S24 and S25). Last, the MD simulations were clustered and the centroid of the clustered simulations was optimized by quantum mechanical, molecular mechanical (QM/MM) simulations (Fig. 4A and fig. S21). Upon inspection of the DAH model in the equatorial-oxo conformation, we found that Thr^231^ and Asn^262^ were located proximal to the Fe(IV)-oxo and speculated about their ability to perform second-sphere interactions that favor halogenation over hydroxylation outcome, similar to Ser^189^ and Asn^219^ residues in bacterial 2ODHs WelO5 (*8*) and BesD (*9*), respectively (fig. S26). However, when in vitro assays were performed using T231A and N262A DAH mutants against dechloroacutumine, we observed no significant change in acutumine production, suggesting that the active site of DAH does not depend on a hydrogen bond donor for the oxo to achieve its halogenation specificity (fig. S26).

Upon further examination of both equatorial-oxo and axial-oxo DAH conformers, we found that Lys^205^ was near the substrate-binding pocket and speculated about its involvement in substrate positioning (fig. S27). When the K205A DAH mutant was generated and tested in vitro, it completely abolished the halogenation activity (Fig. 4C). Subsequently, we performed classical generalized Born energy decomposition analysis (GBSA) on the dominant cluster of the clustered MD simulations and found that K205 contributes −9.7 kcal/mol in energy, suggesting that Lys^205^ likely plays a role in substrate positioning to maintain the obtuse oxo-Fe(IV)-H target angle (Fig. 4B and figs. S27 to S31). We also investigated several active-site–lining residues around the substrate binding pocket, notably Lys^332^, Leu^115^, Tyr^227^, Leu^333^, Arg^121^, Tyr^126^, Val^222^, Leu^135^, Val^221^, Phe^137^, Phe^296^, and Arg^108^, which belong to the top 15 most strongly interacting residues based on GBSA energy (Fig. 4, A and B, and figs. S27 to S31). To test the potential role of these residues in DAH’s catalytic function, we generated alanine mutants for these active-site–lining residues and tested their activity against dechloroacutumine. We found that K332A, Y227A, L333A, Y126A, V222A, and F296A exhibit significant decreases in halogenation activity, whereas R121A, R108A, L135A, L115A, V221A, F137A, and an R108A/R121A double mutant show no particular difference compared to that of wild-type (WT) DAH (Fig. 4C). These findings suggest that residues with high GBSA dechloroacutumine interaction energies are generally important for DAH halogenation activity, likely through key noncovalent interactions that facilitate favorable substrate positioning, as supported by the diminished activity observed in their alanine mutants (fig. S32).

With the structural models of DAH and FLS, we sought to test the capacity to evolve halogenase activity by incorporating structural characteristics from DAH that are pivotal for halogenation into FLS. First, we generated the FLS D230G mutant and tested its oxidase or halogenase activity against dechloroacutumine and dihydrokaempferol. However, the FLS D230G mutant did not show any detectable activity against either dechloroacutumine or dihydrokaempferol (Fig. 4E and fig. S33). As evolutionary analyses revealed the importance of other residues beyond the characteristic D-to-G mutation, we identified three structural features that likely contribute to the functional divergence between DAH and FLS: substrate positioning lid-loop (residues 119 to 127), Fe(II)-coordinating β sheet loop (residues 224 to 234), and C-terminal helical loop near the substrate pocket (residues 318 to 351) (Fig. 2, C and D, and fig. S12). FLS mutants containing DAH sequences in each region were produced and analyzed for halogenase activity via in vitro assay. The FLS_(6)_ mutant, which involves the replacement of the Fe(II)-coordinating β sheet loop with its corresponding DAH sequence, does not exhibit any halogenase activity (Fig. 4, D and E, and fig. S33). Moreover, swapping both the β sheet loop and substrate positioning loop regions, represented as the FLS_(14)_ mutant, showed no significant changes in halogenase activity (Fig. 4, D and E, and fig. S33). Swapping the C-terminal helical loop region, in addition to the β sheet loop and substrate positioning loop regions, resulted in the FLS_(44)_ mutant that exhibits detectable halogenase activity toward dechloroacutumine, although such activity is only 1.9% of the WT DAH activity (Fig. 4, D and E, and fig. S33). Conversely, substituting these three structural regions’ corresponding FLS sequences in DAH resulted in increased production of kaempferol when reacted with dihydrokaempferol (figs. S15 and S32).

### Exploration of evolutionary paths to halogenase from other plant 2ODDs

**Fig. 5.**
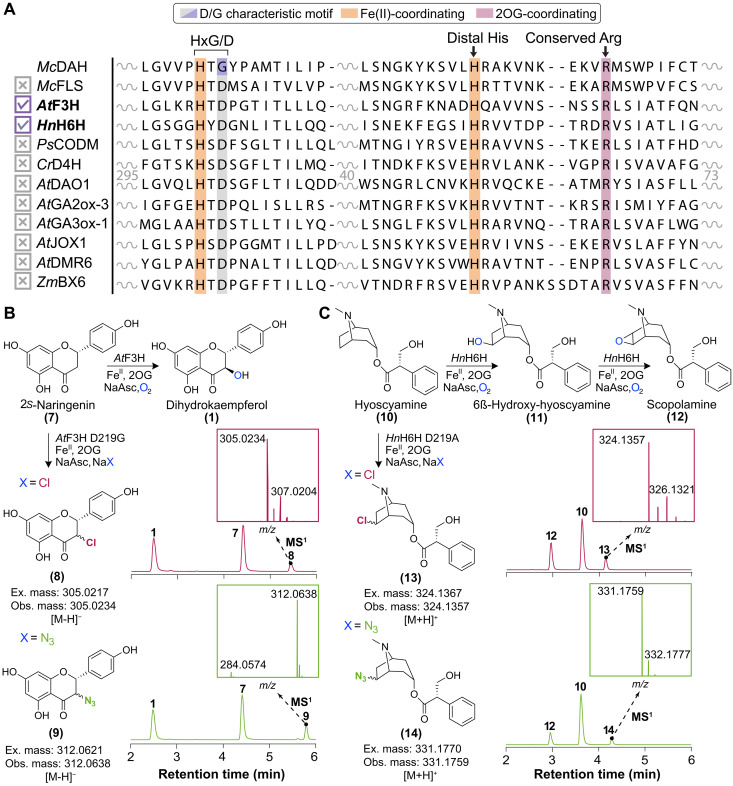
Mechanism-based engineering of plant 2ODDs toward C─H functionalization activities. (**A**) MSA of select plant 2ODDs with DAH and FLS. Active-site histidines are highlighted in orange. Conserved arginine mutation observed in plant 2ODDs is highlighted in red. (**B**) Engineered C─H functionalization of 2*S*-naringenin using *At*F3H D219G variant. EICs showing the in vitro activity of F3H D219G variant that performs alternative anion installations on 2*S*-naringenin, **7** in reaction buffers containing 1 mM NaX, where X = Cl, chlorination of **7** to 9-chloro-naringenin, **8** and X = N_3_, azidation of **7** to 9-azido-naringenin, **9**. The native hydroxylated product, dihydrokaempferol, **1** is also produced as a side product in each EICs. The EICs are scaled to their relative ion intensity. Mass windows used for displaying the EICs: **1**, 287.057 *m/z*; **7**, 271.062 *m/z*; **8**, 305.023 *m/z*; **9**, 312.063 *m/z*. (**C**) Engineered C─H functionalization of hyoscyamine using hyoscyamine-6β-hydroxylase D219A variant. EICs showing the in vitro activity of H6H D219A variant that performs alternative anion installations on **10** in reaction buffers containing 1 mM NaX, where X = Cl, chlorination of **10** to **13** and X = N_3_, azidation of **10** to **14**. The native hydroxylation product **12** is also produced as a side product in each EICs. The EICs are scaled to their relative ion intensity. Mass windows used for displaying the EICs: **12**, 304.155 *m/z*; **10**, 290.175 *m/z*; **13**, 324.137 *m/z*; **14**, 331.177 *m/z*.

Given the challenges in evolving DAH function from FLS, we further explored this rare characteristic mutation in the widely distributed plant 2ODDs. We selected 10 plant 2ODDs that catalyze various oxidation reactions including hydroxylation and *O*-demethylation on metabolites ranging from flavonoids and alkaloids to phytohormones and phytotoxins: *A. thaliana* flavonone-3-hydroxylase (*At*F3H), *Hyoscyamus niger* hyoscyamine-6-β-hydroxylase (*Hn*H6H), *P. somniferum* codeine-*O*-demethylase (*Ps*CODM), *Catharanthus roseus* desacetoxyvindoline-4-hydroxylase (*Cr*D4H), *A. thaliana* dioxygenase-for-auxin-oxidation-1 (*At*DAO1), *A. thaliana* gibberellin-2-oxidase-3 (*At*GA2ox-3), *A. thaliana* gibberellin-3-oxidase-1 (*At*GA3ox-1), *A. thaliana* jasmonate-induced-oxygenase-1 (*At*JOX1), *A. thaliana* downy-mildew-resistant-6 (*At*DMR6), and *Zea mays* DIBOA-glucoside-dioxygenase (*Zm*BX6). Upon examining their MSA with DAH and FLS, we generated the D-to-G or D-to-A mutations at the iron-binding acidic residue for each 2ODD sequence (Fig. 5A). Of the 10 plant 2ODDs attempted in this exercise, only 2 showed alternative reaction outcomes.

First, we investigated *At*F3H that catalyzes the conversion of 2*S*-naringenin to dihydrokaempferol, a key step during flavonoid biosynthesis in land plants. The *At*F3H D219G variant was recombinantly expressed in *Escherichia coli* and assessed for its activity on 2*S*-naringenin under NaCl conditions, with a direct comparison to assay performed using the WT *At*F3H (Fig. 5B). We observed a new product peak that corresponds to the mass/charge ratio (*m/z*) value of an alternative chlorinated product, which also displayed an isotope distribution consistent with chloride incorporation (Fig. 5B). As previous characterization of DAH has extended catalytic activities to install alternative anions, we tested *At*F3H D219G’s ability to derivatize 2*S*-naringenin using alternative anions like N_3_^−^ and Br^−^. In addition to chlorination, we show that *At*F3H D219G is capable of installing azide and bromide when reacted under NaN_3_ or NaBr conditions, respectively (Fig. 5B). Next, we examined *Hn*H6H D219A that catalyzes the hydroxylation of hyoscyamine to scopolamine in tropane alkaloid biosynthesis. Similarly to the *At*F3H D219G case, we observed site-selective installation of chloride and azide on hyoscyamine, as indicated by the new peaks that correspond to the *m/z* and their predicted mass isotope patterns (Fig. 5C).

In both cases, the regio- and stereo-specificity of the anion-installed moieties are not directly measured but can be reasonably postulated to be at the same ─OH group that WT *At*F3H and *Hn*H6H installs onto 2*S*-naringenin and hyoscyamine, respectively. This postulation aligns with the manner in which substrate binding and C─H bond abstraction have been documented in other carrier-independent bacterial 2ODHs and DAH (*4*, *8*). Although *At*F3H D219G and *Hn*H6H D219A exemplify successful cases of D-to-G/A point mutations that facilitate halogenation through opening an anion coordination position in the active site, the mutants still preferred hydroxylation outcome as indicated by higher existing peaks corresponding to their native products (Fig. 5, B and C). Furthermore, eight other selected 2ODD D-to-G/A mutants all did not exhibit any halogenation activity against their native substrates (Fig. 5A). It was noted that all mutants were difficult to express and purify as recombinant proteins in *E. coli* (Fig. 5A). This set of experiments suggest that although the D-to-G/A point mutation is key to the mechanistic switch from a hydroxylase to a halogenase in DAH evolution, it is likely preceded by many other mutations that enabled an continuously viable Darwinian mutational trajectory.

### Estimating the evolutionary timing of DAH emergence in *Menispermum*

**Fig. 6.**
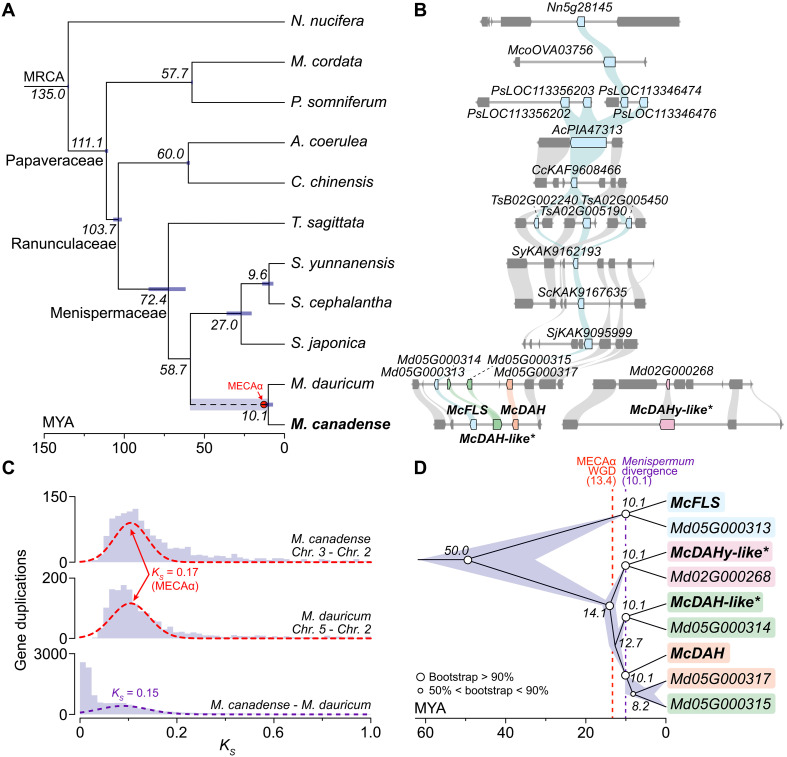
Phylogenomic and syntenic analyses of duplication, neofunctionalization, and pseudogenization events in DAH evolution. (**A**) Phylogenomic divergence of *M. canadense* compared to nine other Ranunculales species with *N. nucifera* as outgroup; *M. cordata*, *P. somniferum*, *Aquilegia coerulea*, *C. chinensis*, *T. sagittata*, *S. yunnanensis*, *S. cephalantha*, *S. japonica*, *M. dauricum*. Mean divergence times in MYA are shown in black; 95% confidence intervals (CI) in purple. A *Menispermum-*specific WGD event (MECAα) is marked by a red circle [13.4 MYA, from (C)]. (**B**) Syntenic analysis of genomic regions containing *FLS/DAH* orthologs across Ranunculales. Cyan ribbons denote *FLS-like* gene synteny; green, orange, and pink ribbons denote *DAH-like*, *DAH*, and *DAHy-like* synteny, respectively. Gene names are labeled; asterisks denote pseudogenes. (**C**) *K_S_* distributions of syntenic blocks between *M. canadense* Chr. 3 and 2 (top), *M. dauricum* Chr. 5 and 2 (middle), and between the two species (bottom). Mixtools analysis shows peaks at *K_S_* = 0.17 (MECAα WGD) and *K_S_* = 0.15 (species divergence). (**D**) Bayesian-inferred phylograms of *DAH* and *FLS* gene families from *M. canadense* and *M. dauricum*, calibrated with divergence times from (A) and *K_S_*-based estimates (*T = K_S_*/2*r*, *r* = 6.5 × 10^−9^ mutations per site per year commonly used for eudicots). Purple shows individual runs; consensus tree is black. Asterisks denote pseudogenes.

To further infer the timing of DAH emergence in relation to the divergence of the *Menispermum* genus, we conducted expanded phylogenomic and synteny analyses across selected Ranunculales species using the *Nelumbo nucifera* genome as an outgroup. Syntenic blocks containing *FLS*, *DAH*, *DAH-like*, and *DAHy-like* were identified and compared in Papaveraceae (*M. cordata* and *P. somniferum*), Ranunculaceae (*A. coerulea* and *C. chinensis*), and Menispermaceae (*Tinospora sagittata*, *Stephania yunnanensis*, *Stephania cephalantha*, *S. japonica*, and *Menispermum dauricum*) (Fig. 6, A and B). Synteny analysis across Ranunculales species reveals that *DAH, DAH-like, and DAHy-like* orthologs are only found in *M. dauricum*, whereas orthologs of *FLS* are conserved across the syntenic blocks (Fig. 6B and fig. S34). To refine the timing of MECAα in the context of DAH locus evolution, we recalculated the *K_S_* of syntenic gene pairs specifically between chromosomes 3 and 2 of the *M. canadense* genome assembly and chromosomes 5 and 2 of the recently published *M. dauricum* genome assembly (*33*), as these regions encompass the WGD event most relevant to the DAH gene cluster (Fig. 6C and fig. S34). The divergence time was estimated using the formula T = *K_S_*/2*r*, where *r* represents a substitution rate of 6.5 × 10^−9^ mutations per site per year for eudicots. On this basis, we projected a mean divergence time of 13.4 MYA (12.0 to 6.09 MYA, 95% confidence interval; CI) for these chromosomal duplications. This MECAα duplication event occurred before the divergence of *M. canadense* from *M. dauricum*, 10.1 MYA (12.1 to 3.39 MYA, 95% CI), and thus suggests that DAH likely existed before the radiation of the *Menispermum* genus (Fig. 6, C and D). Building on these phylogenomic dating nsights, we constructed a Bayesian-inferred phylogenetic tree of *FLS*, *DAH*, *DAH-like*, and *DAHy-like* orthologous genes in *M. canadense* and *M. dauricum* (Fig. 6D). This phylogeny, which traces the birth of *DAH*, indicates that *DAHy-like* diverged from its progenitor *FLS* approximately 50 MYA within the *Menispermum* genus (Fig. 6D). We estimate that the common ancestor of *DAH* and its two intermediate genes existed before the MECAα duplication event (~14.1 MYA) and that *DAH* likely diverged from *DAH*-like around 12.7 MYA—before the split between *M. canadense* and *M. dauricum* (Fig. 6D). Complementing this result, a maximum-likelihood phylogenetic tree of FLS, DAH, DAH-like, and DAHy-like orthologs across 11 Ranunculales species reveals that DAH, DAH-like, and DAHy-like from *M. canadense* and *M. dauricum* are not nested within their respective FLS clades (fig. S35). This phylogenetic pattern further supports the inference that *DAH* underwent neofunctionalization before the speciation event (Fig. 6D and fig. S35).

## DISCUSSION

**Fig. 7.**
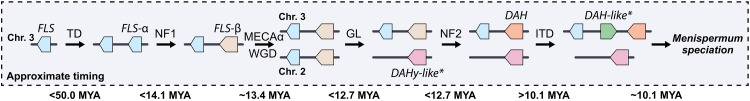
Proposed model of *DAH* emergence from a *FLS* progenitor in *M. canadense*. Estimated times of each evolutionary event are inferred from Bayesian phylogenomic analyses. Wheat-colored boxes indicate intermediates not captured in the genome. Asterisks denote pseudogenes. Abbreviations: TD, tandem duplication; NF1, neofunctionalization event to yield putative ancestral *FLS-*β (not captured in the genome); MECAα WGD, *Menispermum-*specific whole-genome duplication; GL, gene loss; NF2, neofunctionalization event via D-to-G catalytic switch mutation; ITD, inverted tandem duplication.

The emergence of DAH in *M. canadense* is the only appearance of a halogenase in specialized metabolism across all land plant species reported to date. Our analyses, on the basis of the chromosomal-level genome assembly of *M. canadense*, reveal a series of tandem duplications (TDs) and WGD events that illuminate the evolutionary trajectory from FLS to DAH (Fig. 7). We suspect that the TD of *FLS* initially resulted in two tandem copies of *FLS* on *M. canadense* chromosome 3 sometime within the past 50 MYA, where the TD copy is referred to as *FLS-*α (Fig. 7). *FLS-*α likely acquired inserted sequences encoding the C-terminal extension observed in DAH, which is presumed to be critical for the substrate switch from dihydroflavonol to dechloroacutumine. This evolutionary intermediate is represented as *FLS-*β, although whether it harbored DAHy activity remains unknown (Fig. 7). Subsequently, the *Menispermum*-specific WGD event (MECAα) was observed in *M. canadense* by the presence of chromosome 2, a duplicated copy of chromosome 3. The resulting *FLS* on chromosome 2 likely underwent gene loss following WGD, which is a commonly observed evolutionary process in many angiosperms to favor single-copy conservation of critical metabolic flux-controlling genes (Fig. 7) (*33*). At this point, our data are insufficient to conclude whether either *FLS-*β on chromosomes 3 or 2 encoded DAHy activity in evolutionary history. However, it is clear that *FLS-*β on chromosome 2 eventually underwent pseudogenization to yield *DAHy-like*, which shares 88.5% amino acid sequence identity with DAH while still retaining its hydroxylase sequence motif (Fig. 2, B and C, and Fig. 7). We postulate that *FLS-*β on chromosome 3 acquired the functional D-to-G mutation that yielded a functional copy of *DAH* (Fig. 7). Given that the exon-intron structure of *DAH-like* is drastically different from other paralogous genes, we hypothesize that it originated from an inverted TD event of *DAH* on chromosome 3, which introduced a truncation in its second exon (Fig. 2, C and D, and Fig. 7).

Perhaps the most remarkable feature of the proposed evolutionary model is the retention of the intermediate genes *DAHy-like* and *DAH-like* in the *M. canadense* genome as it provides insights into several hypotheses regarding the evolution of DAH. First, the observation of a naturally occurring allele of *DAHy-like* that contains nonsense mutations at the codon of Lys200 (AAA to TAG), resulting in a premature stop codon, is indicative of nonsense-mediated decay (*34*). This evolutionary purging of the intermediate gene is further supported by its low expression and the sparse mapping of RNA-seq reads. A similar process of pseudogenization is observed in *DAH-like*, as the internal truncation in its exon 2 likely disrupts proper protein folding and leads to loss of catalytic activity, consistent with the difficulties encountered during its recombinant expression and purification from *E. coli*. When examined in a comparative genomic context, synteny analysis reveals that these genomic intermediates are also present in the *M. dauricum* genome. However, the evolutionary processes—such as pseudogenization, gene loss, and neofunctionalization—may have followed different trajectories compared to *M. canadense*, as evidenced by the presence of two *DAH-like* orthologs in *M. dauricum* and the dynamic nature of gene duplication, mutation patterns, alignment variability, and differential rates of pseudogene purging across plant genomes (Fig. 6B and figs. S34 and S36). These lineage-specific differences likely arose after the speciation event (~10.1 MYA) (Fig. 7). Moreover, these relics of evolutionary intermediates also provide genomic evidence suggesting the relative order of mutational events responsible for the catalytic function switch from flavonoid to alkaloid biosynthesis. Because of the high conservation of the substrate-positioning loop, Fe(II)-coordinating β sheet loop, and C-terminal loop regions in *DAHy-like*, *DAH*, and *DAH-like*, we postulate that latent mutations were present in *DAH* before the D-to-G mutation was introduced. We speculate that these mutations enabled the expansion of substrate preference from dihydroflavonol to dechloroacutumine, as demonstrated by DAH’s retention of ancestral FLS activity toward dihydrokaempferol (fig. S17).

The importance of latent mutations in facilitating ultimate mechanism-switching mutations is also underscored by our biochemical investigation of the FLS_(44)_ mutant and select D-to-G/A mutants of select plant 2ODDs. These protein engineering experiments reaffirm the notion that achieving catalytic function conversion between ancestral and derived activities often requires more than simply mutating mechanism-dictating residues. This was previously demonstrated by a study of 311 catalytic variants of WT serum paraoxonase (PON1), generated through random mutagenesis, which found that all variants with altered substrate specificity harbored latent mutations at residues unrelated to direct catalytic functionality (*35*). A similar observation was also made in the halogenase engineering of the bacterial 2ODD MBT76 from *Streptomyces* sp., where a DNA shuffling library of the MBT76 D144G variant and a native lysine halogenase, BesD, identified 13 additional distal residues essential for the full functional switch (*13*). However, identifying the corresponding residues in FLS proved challenging because of the substantial structural divergence between bacterial and plant 2ODDs (fig. S26D). Although this laboratory-directed evolution exercise of halogenase began with the D-to-G mechanism-switching mutant and subsequently improved desirable activity through additional mutations, our work on the natural evolution of DAH suggests an opposite order of mutational events, where the D-to-G mutation occurred at a much later stage of the evolutionary trajectory leading to the emergence of DAH. Moreover, the difficulty in achieving even minimal halogenase activity in the FLS_(44)_ mutant suggests that the evolutionary landscape between ancestral 2ODDs and derived 2ODHs is highly rugged, with deep fitness valleys (*36*), likely limiting the natural evolution of more 2ODHs from the abundant plant 2ODDs.

Previous mechanistic studies of 2ODHs have investigated the significance of specific residues and substrate positioning angles that increase the favorability of chlorination relative to hydroxylation. Our structural modeling of DAH and subsequent GBSA analysis revealed key residues involved in strong noncovalent interactions with the substrate such as Lys^205^, Tyr^227^, and Lys^332^ that were further validated with alanine scanning mutagenesis. Through restrained MD simulations, we also found that DAH favors the obtuse oxo-Fe(IV)-H target angle over acute angles as observed for other halogenases, further explaining its preference for halogenation over hydroxylation (figs. S24 and S25) (*32*, *37*, *38*). Future efforts in elucidating the crystal structure and catalytic mechanism of DAH will further resolve its reactivity preference as similarly showcased in bacterial 2ODH, BesD (*39*, *40*).

Late-stage, regio- and stereo-selective C─H functionalization of natural product scaffolds represents a valuable “on-demand” reaction but remains challenging with traditional organic synthesis methods. Plant natural products are rarely halogenated, yet a substantial number of small molecule drugs contain a halogen, as halogen atoms play pivotal roles in modulating their pharmacological potency, pharmacokinetic stability, and physicochemical properties (*41*, *42*). Understanding the catalytic and evolutionary mechanisms of halogenating enzymes like DAH and BesD will provide insights for designing enzymes capable of performing these on-demand reactions, thereby expanding the functional scope of plant natural products as drug candidates. With the advent of large language models (LLMs) for biocatalyst design, such as ProteinMPNN (*43*) and proseLM (*44*), future computationally assisted enzyme engineering will harness a wide array of plant 2OGDs to enable the installation of C─H substituents for medicinal compound derivatization. These approaches will likely also illuminate viable evolutionary trajectories that navigate the narrow paths on a rugged evolutionary landscape. The natural evolutionary trajectory from FLS to DAH as preserved in Menispermaceae, will serve as a valuable “ground-truth” resource for LLMs, guiding the design and engineering of more plant 2ODDs into 2ODHs.

## MATERIALS AND METHODS

### Sample collection, processing, and sequencing

*M. canadense* plants were purchased from Toadshade Wildflower Farm (Frenchtown, NJ, USA) and grown in a greenhouse at the Whitehead Institute for Biomedical Research (Cambridge, MA, USA). *M. canadense* leaf tissues were harvested and flash-frozen in liquid nitrogen after approximately 6 weeks of growth upon breaking dormancy. High-molecular genomic DNA was extracted using the NucleoBond TakaraBio HMW DNA Extraction kit, quality-controlled by Femtopulse (Agilent Technologies). This DNA was used for PacBio library preparation and SMRT sequencing (Sequel II) for HiFi read generation at the Genomics Core Facility at the Icahn School of Medicine, Mount Sinai, along with PhaseGenomics Hi-C library preparation and Illumina sequencing. We collected 48.57 Gb of SMRT HiFi sequencing data (~50× coverage) from PacBio Sequel II platform (Pacific Biosciences) used for initial assembly. A total of 270.18 Gb NGS data (~303× coverage) were generated using Illumina NovaSeq 6000 (Illumina) for quality assessment and mapping onto the final genome assembly. Moreover, 162.63 Gb Hi-C data (~168× coverage) were generated using Illumina NovaSeq 6000 (Illumina) for scaffolding the initial assembly onto chromosomal scaffolds. Fastp (v 0.21.0) (*45*) was used to assess the quality of sequencing reads.

### Genome size estimation by cell-cytometry and k-mer analysis

Plant homogenates for analysis of *M. canadense* nuclei were prepared by excising the *M. canadense* leaf sample submerged in nuclei isolation buffer [45 mM MgCl_2_, 30 mM sodium citrate, and 20 mM MOPS (pH 7.0)] using a razor blade on a petri dish. The resulting homogenates were filtered through a 50-μm disposable filter and loaded onto the flow cytometer (BD Accuri C6 Cytometer) to assess the relative fluorescence intensity of nuclei in suspension and estimate the genome size (in picograms of DNA). The resulting flow cytometry measurement for *M. canadense* nuclei was compared to the control sample, *Solanum lycopersicum* (tomato) nuclei to arrive at the estimation of ~925 Mb for *M. canadense* genome size. Moreover, the genome size of *M. canadense* was computationally estimated using a *k*-mer (k = 19) frequency–based approach with the Illumina paired-end short reads. The software jellyfish (v 2.3.0) (*24*, *46*) was used to count the *k*-mers and visualized using GenomeScope (v2.0) (*47*), which was also used for estimating the genome size, resulting in a predicted genome size of ~958 Mb.

### Genome assembly, quality assessment, and annotation

To generate the initial contig-level assembly of *M. canadense*, Hifiasm (v0.15.2) (*48*) was used with HiFi reads in Hi-C mode and default settings. The initial contig sequence was used as the assembly for scaffolding with Hi-C reads using juicer (v1.6) (*49*). 3D-DNA (v190716) (*50*) was used to generate Hi-C contact maps with options --editor-repeat-coverage 5 and --splitter-coarse-stringency 30, and the scaffolds were assembled into chromosomes using Juicebox (v1.11.08) (*51*). Both the initial contig-level assembly and postscaffolding chromosomal-level assembly statistics were determined using quast (5.2.0) (*52*).

The gene completeness of the assembly were assessed with BUSCO (v5.1.3) (*16*) using hmmsearch (v3.1) (*53*), metaeuk (v4.a0f584d) (*54*), and embryophyta_odb10 database. Quality assessment of the proteome of *M. canadense* was performed using the OMArk web server (*17*) (https://omark.omabrowser.org; OMAmer database: OMA / All.Jul2023 / LUCA | OMAmer version: 2.0.3 | OMArk version: 0.3.0) with default parameters. The *k*-mer (*k* = 20)–based genome assessment using merqury (v1.3) (*18*) was performed with the postscaffolded chromosomal-level assembly of *M. canadense* including unscaffolded contigs and 48.57 Gb of SMRT HiFi sequencing data. For counting RNA-seq reads mapped to the 26 pseudo-chromosomes, bwa-mem (v2.2.1) and samtools (v1.16) with options view -c and -c -F 260 found 1,815,513,564 primary aligned reads of 1,838,251,865 total reads. RNA-seq reads used for mapping are from our previous work deposited in NCBI SRA (accessions SRR10947794-SRR10947801). Kallisto (v0.48.0) was used to quantify RNA-seq data.

The resulting 26 chromosomes of *M. canadense* were annotated using the Funannotate pipeline (v.1.8.7) (*55*). First, the chromosomal-level genome assembly was soft-masked using tantan, with repeats and transposable elements soft-masked before gene model prediction using PASA (v.2.4.1) with *M. canadense* RNA-seq reads, de novo assembled transcripts, and protein homology evidence as input. The gene models and protein homology evidence were then used to train Augustus (v.3.3.3), GeneMark-ES (v.4.61), SNAP (v.2006-07-28), and Glimmerhmm (v.3.0.4) ab initio gene predictors and predicted genes passed to Evidence modeler (v.1.1.1) with various weights for integration. tRNAscan-SE (v.2.0.7) was used to predict nonoverlapping tRNAs. Transcript evidence from our previous de novo transcriptome assembly (*4*) using Trinity (v.2.8.5) was leveraged to correct, improve, and update the predicted gene models, in addition to refining the 5′- and 3′-untranslated regions in the final step with Funannotate (v.1.8.7). Moreover, the sequence and annotation of DAH paralogous genes that are highlighted in this study were corrected upon amplification of their genomic locus using polymerase chain reaction and validated with their Sanger sequencing results. The corrected sequences are reported in table S10.

### Phylogenomic analysis

To examine the phylogenetic relationships of *M. canadense* with other related species, we selected 12 additional species for WGD events (tables S6 and S7). The protein sequences of each species were collapsed using the 90% identity threshold with cd-hit (v4.5.4) (*56*). The homologous groups among all 13 species were identified using the OrthoFinder (v2.5.4) (*57*) with settings -S diamond -M msa -A muscle -T raxml-ng -I 1.5. Following OrthoFinder analysis, the amino acid sequences of single-copy orthologous genes from 13 species were aligned using MAFFT (v7.402) (*58*) with L-INS-i method and options –maxiterate 1000 –leavegappyregion. Then, the protein alignments were reverse-transcribed into their coding sequences using pal2nal (v14) (*59*) and trimmed using trimAl (v1.2rev59) (*60*). Phylogenetic trees of each single-copy genes were constructed using RAxML (v8.2.11) (*60*, *61*) according to the model of GTRGAMMA with 1000 bootstrap replicates and *A. trichopoda* as the outgroup species (options -# 1000 -o Atri, -x 12345 -p 25258 -f a -T 2). Following gene tree constructions, a consensus species tree was inferred using ASTRAL-III (*62*) with 1000 bootstraps. To estimate species divergence times, the Bayesian relaxed molecular clock method in MCMCtree (v4.9j) (*20*) was used with the F84 model and the divergence times of *M. canadense*–*S. japonica* (~35.4 to 115.2 MYA), *M. cordata*–*P. somniferum* (~44 to 82 MYA), *A. coerulea*–*C. chinensis* (~25.7 to 79.6 MYA), Ranunculales plants (~103 to 118 MYA), and monocots-dicots (~130 to 240 MYA) as estimated by a previous study (*63*) and TimeTree (*64*). CAFE (v5.0) (*65*) was used in the Base model and single lambda to infer gene family expansion and contraction in the species tree. For the Ranunculales-specific species tree, orthogroups among 11 species were generated resulting in 268 single-copy orthologous sequences. For the Ranunculales-specific species tree, orthogroups were identified across 11 species, resulting in 268 single-copy orthologous sequences. Phylogenetic analyses were conducted using the same approach as for the broader species tree, with the exception of divergence time calibrations. These calibrations were based on estimated divergence times from the previous tree: *Stephania-Menispermum* (~58.7 MYA), *A. coerulea–C. chinensis* (~60 MYA), Ranunculaceae-Menispermaceae (~102 MYA), Papaveraceae-Ranunculaceae (~111 MYA), and *N. nucifera–*Ranunculales (~135 MYA).

### Phylogenetic tree construction and positive selection analysis

An aBSREL was performed on the gene-tree containing FLS orthologous genes from select angiosperm species as highlighted in Fig. 2F and their nucleotide MSA using the aBSREL method (*66*) within the HyPhy program (v2.3.11) (*67*). The input MSA contained 14 sequences with 381 sites (codons). One branch of the gene phylogeny leading to the clade containing *DAH*, *DAH-like*, and *DAHy-like* was formally tested for diversifying selection. The aBSREL analysis found evidence of episodic diversifying selection on this node in the phylogeny with significance at *P* value of 0.016 after the Holm-Bonferroni correction for multiple hypothesis testing. The intermediate files and results of this analysis, including the nucleotide MSA, General Time Reversible (GTR)-based gene-tree, and aBSREL-produced adaptive rate class model gene tree are available in the Supplementary Materials. The corresponding positive selection aBSREL result was mapped onto the protein-based maximum-likelihood phylogenetic tree constructed in Fig. 2F. Protein sequences were aligned using the MUSCLE (*68*) algorithm in MEGAX (*69*). Evolutionary histories were inferred by using the maximum-likelihood method on the basis of the Jones, Taylor, and Thornton (JTT) matrix–based model. Bootstrap statistics were calculated using 200 replicates. All phylogenetic analyses were conducted in MEGAX (*69*). All alignment files can be found in the Supplementary Materials.

### Synteny analysis and assessment for WGD

To perform macro- and microsynteny analyses between plant genomes explored in this study, BLASTP (v. 2.15.0+) was used to calculate pairwise similarities (*e* value <1 × 10^5^) between CDS DNA of the plant species, and MCScanX (*70*) was used with default parameters to identify synthetic gene pairs. MCScanX was further used to visualize the syntenic blocks and assess syntenic depths between plant genomes. Moreover, the DupPipe pipeline from EvoPipes (*22*) was used to calculate the *K*_S_ values. The distribution of *K*_S_ values were analyzed using Mixtools (*24*) with *k* = 2 and 200 bootstraps at an epsilon value of 0.001 for most analyses. To examine the divergence between plant species, the OrthoPipe pipeline from EvoPipes (*22*) was used to calculate the *K*_S_ values between two genomes in comparison. The distribution of these *K*_S_ values was analyzed using Mixtools with *K*_S_ max value increased to 3 and the epsilon value changed to 0.01.

### Protein expression and purification

All genes encoding for relevant *Mc*DAH and *Mc*FLS enzymes in this study were codon-optimized for heterologous overexpression in *E. coli* and purchased as a synthetic gene (Integrated DNA Technologies). Then, they were cloned into the pHis8-4b expression vector containing an *N*-terminal 8xHis tag followed by a tobacco etch virus (TEV) cleavage site (table S8). The sequence-verified constructs were transformed into *E. coli* BL21 (DE3) for recombinant protein expression (tables S9 and S10). A 1-liter culture of terrific broth (TB) medium containing kanamycin (50 μg/ml) was inoculated with 30 ml of an overnight starter culture and allowed to grow with shaking at 200 rpm at 37°C to an optical density at 600 nm of 0.6 to 0.8. Then, protein expression was induced by addition of 0.5 mM isopropyl β-d-1-thiogalactopyranoside followed by cold shock of the medium and subsequent growth with shaking at 200 rpm (18°C for 18 hours).

Cultures were harvested by centrifugation, and the resulting cell paste (~10 g/liter) was resuspended in lysis buffer [100 mM tris (pH 8.0), 200 mM NaCl, 20 mM imidazole, 10% (v/v) glycerol, and 1 mM dithiothreitol] containing lysozyme (1 mg/ml) and 1 mM phenylmethylsulfonyl fluoride. The cells were lysed by sonication at 60% amplitude: 30-s on, 30-s off for 10 min using the flat tip for one-half–inch (1.27 cm) diameter disruptor horn (Branson Ultrasonics Corporation Sonifier SFX550 Cell). The resulting crude protein lysate was clarified by centrifugation (19,000*g*, 45 min) before QIAGEN nickel–nitrilotriacetic acid (Ni-NTA) gravity flow chromatographic purification. After loading the clarified lysate, the Ni-NTA resin was washed with 20 column volumes of lysis buffer and eluted with 1 column volume of elution buffer [100 mM tris (pH 8.0), 200 mM NaCl, 250 mM imidazole, 10% (v/v) glycerol, and 1 mM dithiothreitol]. Then, 1 mg of His-tagged TEV protease was added to the eluted protein, followed by dialysis at 4°C for 16 hours in dialysis buffer [25 mM tris (pH 8.0), 200 mM NaCl, 5% (v/v) glycerol, 5 mM EDTA, and 0.5 mM dithiothreitol]. After dialysis, protein solution was passed through Ni-NTA resin to remove uncleaved protein and His-tagged TEV. The recombinant proteins were further purified by gel filtration on an ÄKTA Pure fast protein liquid chromatography (LC) system (GE Healthcare Life Sciences). The principal peaks were collected, verified by SDS–polyacrylamide gel electrophoresis, and dialyzed into a storage buffer [25 mM tris (pH 8.0) and 5% (v/v) glycerol]. Last, proteins were concentrated to appropriate concentrations using Amicon Ultra-15 Centrifugal Filters (Millipore).

For recombinant expression of 2ODD mutants—*At*F3H D219A, *Ps*CODM D237A, *Cr*D4H H270A, *At*DAO1 H178A, *At*GA2ox-3 H204A, *At*GA3ox-1 H234A, *At*JOX1 H275A, *At*DMR6 H214A, and *Zm*BX6 H245A—codon-optimized gene fragments were purchased as synthetic genes and then cloned into the pHis8-4b vector. For *At*F3H, the D219G mutant was also generated and cloned into the pHis8-4b vector. For recombinant expression of *Hn*H6H D219A, codon-optimized gene fragment was cloned into the plasmid pBA0221-0141 (*71*) containing a C-terminal 8xHis following a TEV cleavage site (table S8). After obtaining each of these plasmid constructs, the same protocol as above was initially carried out for all 2ODD mutants. However, most 2ODD mutants—*At*F3H D219A, *Ps*CODM D237A, *Cr*D4H H270A, *At*DAO1 H178A, *At*GA2ox-3 H204A, *At*GA3ox-1 H234A, *At*JOX1 H275A, *At*DMR6 H214A, and *Zm*BX6 H245A—were either found in the insoluble fraction during the protein purification process or exhibit no detectable enzyme activity. For BL21 strains expressing *At*F3H D219G and *Hn*H6H D219A, 12-liter culture of TB medium was used to increase the yield of these relatively unstable mutants.

For recombinant expression of *Mc*DAH alanine mutants, site-directed mutagenesis was performed according to the protocol described in the QuickChange II Site-Directed Mutagenesis Kit (Agilent Technologies) using plasmid pHis8-4b::*Mc*DAH as the template and the primer sequences in table S9. The resulting mutant plasmid constructs were verified by sequencing. Recombinant mutant protein production and purification were carried out following the same procedure as described above.

### In vitro enzyme assays

Each enzyme assay for McDAH-WT and relevant mutants reported in this study with (−)-dechloroacutumine was carried out in 50 mM tris buffer (pH 8.0), on a 20-μl reaction containing the following components: enzyme (5 μM), (−)-dechloroacutumine (1 mM), 2OG (500 μM), NaCl (1 mM), sodium ascorbate (5 mM), and (NH_4_)_2_Fe(So_4_)_2_ (2 mM). In a typical assay, the components were added in the following order: (i) tris, (ii) NaCl, (iii) enzyme, (iv) sodium ascorbate, (v) 2OG, (vi) (−)-dechloroacutumine, and (vii) (NH_4_)_2_Fe(So_4_)_2_. The samples were incubated under aerobic conditions at room temperature for 20 mins. The assays were quenched by adding methanol to 50% final concentration and centrifugation to spin down enzymes and debris. The supernatants were filtered using 0.2-μm filter vials (Thomson Instrument Company) before injection (2 μl) into the Liquid Chromatography-High Resolution Accurate Mass Mass Spectrometry (LC-HRAM-MS) system for analysis. Relevant enzyme assays for activity against dihydroflavonols were performed similarly, but without the addition of (ii) and substitution of (vi) with 1 mM dihydrokaempferol or 1 mM dihydroquercetin, and then quenched by adding acetonitrile with 0.1% formic acid to 50% final concentration. All 2ODD mutant assays were performed similarly using NaCl or NaN_3_ as the salt in the reaction for chlorination or azidation reactions, respectively. For *At*F3H D219G assays, 2*S*-naringenin was used as the substrate, whereas hyoscyamine was used as the substrate for *Hn*H6H D219A assays.

### *LC-HRAM-MS* analysis

LC was conducted on a Vanquish Flex Binary UHPLC system (Thermo Fisher Scientific) using water with 0.1% formic acid as solvent A and acetonitrile with 0.1% formic acid as solvent B. Reverse phase separation of analytes was performed on a Kinetex C18 column, 150 mm by 3 mm, 2.6-μm particle size (Phenomenex). The column oven was held at 35°C. Most injections were eluted with 5% B for 0.5 min, a gradient of 5 to 95% B for 14.5 min, 95% B for 2 min, and 5% B for 3.0 min, with a flow rate of 0.5 ml/min. Most MS analyses were performed on a high-resolution Orbitrap Exploris 120 benchtop mass spectrometer (Thermo Fisher Scientific) operated in positive ionization mode with full scan range of 100 to 450 *m*/*z* and top four data-dependent tandem MS (MS/MS) scans. The orbitrap resolution is 120,000 with radio frequency (RF) lens of 70% and static spray voltage of 3500 V. For detecting dihydroflavonols and flavonols from relevant enzyme assays, injections were eluted with 5% B for 0.5 min, a gradient of 5 to 95% B for 14.5 min, 95% B for 2 min, and 5% B for 3.0 min, with a flow rate of 0.5 ml/min. Moreover, MS analysis was operated in negative ionization mode with full scan range of 100 to 450 *m/z* and top four data-dependent MS/MS scans using static spray voltage of 2500 V. For detecting products from *At*F3H D219G assay, injections were eluted with 20% B for 0.5 min, a gradient of 20 to 50% B for 6.5 min, 50 to 80% B for 0.5 min, 80% B for 0.5 min, and 20% B for 2 min with a flow rate of 0.5 ml/min using a Kinetex C18 column, 50 mm by 3 mm, 2.6-μm particle size (Phenomenex). MS analysis was operated in negative ionization mode with full scan range of 150 to 400 *m/z* and top four data-dependent MS/MS scans using static spray voltage of 2500 V. For detecting products from *Hn*H6H D219A assay, injections were eluted with 5% B for 0.5 min, a gradient of 5 to 30% B for 6.5 min, 30 to 75% B for 0.5 min, 75% B for 0.5 min, and 5% B for 2 min with a flow rate of 0.5 ml/min using the Kinetex C18 column, 50 mm by 3 mm, 2.6-μm particle size. MS analysis was operated in positive ionization mode with full scan range of 15 to 450 *m/z* and top four data-dependent MS/MS scans using static spray voltage of 3500 V. Raw LC-MS data were collected and analyzed using Chromeleon 7.2.10 ES, TSQ Tune 3.1.279.9, and XCalibur 4.5 (Thermo Fisher Scientific).

### Protein structure and preparation

AlphaFold2 was used to generate folded structures for DAH and FLS using the default parameters of the AlphaFold Singularity container for version 2.0.0 (*30*). All subsequent structural models discussed in this study are derived from AlphaFold2 folded structures, and we demonstrate that AlphaFold3 folded structures have no structural differences (fig. S37). Protonation states were assigned using the H++ webserver with a pH of 7.0 and an internal dielectric of 10 while retaining all other system defaults (tables S11 and S12) (*72*–*74*). Core active site residues were reviewed and assigned such that histidines were neutral and the metal-coordinating carboxylate in FLS was negatively charged. Molecular docking runs were performed for enzyme-substrate pairs FLS and dihydrokaempferol and DAH and dechloroacutumine using AutoDock Vina 1.1.2 (fig. S19) (*75*, *76*). We found that dihydrokaempferol had a binding energy of −8.2 kcal/mol, and dechloroacutumine had a binding energy of −7.5 kcal/mol. The lowest energy substrate conformations of dihydrokaempferol and dechloroacutumine were selected as the initial substrate binding poses for FLS and DAH, respectively. Structures of the initial binding poses are provided in the Supplementary Materials as a .zip file. On the basis of the dihydrokaempferol and dechloroacutumine docked complexes, we generated models for both FLS and DAH with either 2OG or succinate and oxo bound. We also generated DAH models with chlorine and the oxo in both the equatorial and axial positions. The ligands 2OG, succinate, Fe, Cl, and oxo were modeled manually into their corresponding structures. The resulting DAH and FLS holoenzymes had an atom count and a net charge of 5536 and -8 for FLS with 2OG, 5535 and -8 for FLS with succinate, 5697 and -9 for DAH with 2OG, 5696 and -9 for DAH with succinate and an equatorial-oxo, and 5696 and -9 for DAH with succinate and an axial-oxo.

Using the tleap utility in the AMBER software suite, topology and coordinate files for the final structures were generated for the AMBER ff14SB force field (*77*). The ligands and substrates were parameterized using the generalized AMBER force field and restrained electrostatic potential charges (*78*). These charges were calculated with Gaussian16 at the HF/6-31G* level of theory (*79*). The core active site parameters were obtained using AMBER’s Metal Center Parameter Builder (MCPB) (*80*). The MCPB.py v3.0 script was used to compute charges with the ChgModB method at the UB3LYP/LACVP* (*81*, *82*) level of theory for iron-coordinated residues. The Seminario method (*83*) was used to derive additional force field parameters, with details available (figs. S20 and S22). Subsequently, each protein system was solvated with a 15-Å TIP3P water box with periodic boundary conditions and neutralized with Na^+^ counterions (*84*). The resulting final atom counts for the various systems were: 64,767 for FLS with 2OG, 64,766 for FLS with succinate, 72,247 for DAH with 2OG, 72,246 for DAH with succinate and an equatorial-oxo, and 72,246 for DAH with succinate and an axial-oxo. The topology and inpcrd files for all MD simulations are provided in the Supplementary Materials as a .zip file.

### Classical MD simulations and analysis

We performed MD simulations for DAH and FLS using AMBER18 and the GPU-accelerated particle mesh Ewald (PME) (*85*) MD (PMEMD) code (*86*, *87*). The equilibration protocol involved three steps: (i) 1000 steps of hydrogen atom minimization, 2000 steps of sidechain minimization with a fixed backbone, and 2000 steps of protein minimization with the core active site restrained; (ii) controlled heating with constant number of particles, constant volume, and constant temperature (NVT) from 0 to 300 K over 10 ps using the Langevin thermostat and a collision frequency of 5.0 ps^−1^; and (iii) a 1-ns simulation with constant number of particles, constant pressure, and constant temperature (NPT) with the Berendsen barostat and a 2-ps relaxation time. Following equilibration, we collected 250 ns of production dynamics for each enzyme-substrate complex, using 2-fs time steps, the SHAKE algorithm to fix hydrogen-heavy atom distances (*88*), and electrostatics were treated with the PME method with a real-space cutoff of 10 Å (*85*). Restrained MD simulations were also carried out for 250-ns production runs for each system with flat-bottom harmonic restraints applied to the oxo, iron, and HAT target angle and the iron-HAT target distance to increase sampling of halogenase and hydroxylase expected angles (table S13). For all systems, at least four 250-ns replicates were performed.

We also performed 250-ns production runs for restrained MD simulations in each system, using harmonic restraints to enhance the sampling of target angles and distances typically observed for halogenases and hydroxylases (tables S14 and S15) (*32*, *38*, *89*, *90*). Such experimentally informed MD simulations have been used in guiding similar computational studies of nonheme iron halogenases and hydroxylases (*32*, *37*, *91*). The harmonic restraints are based on experimental hyperfine sublevel correlation spectroscopy data, which extract relative spatial information (e.g., distances and angles) about the metallocofactor and nearby ^2^H labels on the substrate (*38*, *92*, *93*). To select representative frames for analysis, we clustered trajectories with the DBSCAN algorithm in CPPTRAJ (*94*) using the substrates as masks and following a previously described method (tables S14 and S15) (*91*). Following clustering, the centroid of the largest cluster was used for QM/MM optimization (figs. S21 and S23). We used MMPBSA.py in AMBER18 (*91*, *95*, *96*) for interaction analysis of the selected snapshots using the generalized Born (*37*, *91*) approximation and the OBC1 model. This method computes the contributions to pairwise residue interactions, electrostatics, and van der Waals’ binding (*97*, *98*). We used 1000 snapshots from the end of the simulations, spaced 50 ps apart for GBSA analysis. Classical geometric hydrogen bonding analysis was conducted with the CPPTRAJ utility, applying a hydrogen bonding distance cutoff of 3.2 Å based on previously quantified hydrogen bonding strengths (*98*). For restrained simulations, we used harmonic restraints of 100 kcal/(mol · Å^2^) as previously described (*32*, *37*).

### QM/MM simulations

We ran QM/MM geometry optimizations starting from the centroids of the largest clusters of the MD production runs for all FLS and DAH isomers. Spherical droplets with a radius of 35 to 42 Å, centered around the protein’s center of mass, were next created using PyMOL (*99*) from the periodic box extracted from the MD simulations. Counterions were added using tleap to neutralize the droplets. The QM/MM simulations were conducted using a developer version of TeraChem v1.9 (*100*–*102*) for the QM component and AMBER18 for the MM component (*86*, *87*). In the QM/MM simulations, no atoms were held fixed. A weak restraining spherical cap (force constant of 1.5 kcal/mol^.^Å^2^) was applied to maintain the MM water droplet’s shape and prevent movement during optimization. Unrestricted density functional theory with the range-separated hybrid ωPBEh (ω = 0.2 Bohr^−1^) (*103*) and a basis set consisting of the LANL2DZ effective core potential (*104*) was used for Fe, while 6-31G* was used for all other atoms in the QM region (*81*, *82*). FLS and DAH were modeled in the high-spin state (i.e., quintet multiplicity, 2*S* + 1 = 5). The QM regions of DAH and FLS included their respective substrates, the Fe center, and all ligands and residues coordinating Fe (tables S16 to S19). The only residue from the second coordination sphere included in the QM region was Lys^205^ in DAH and its hydroxylase corollary Lys^209^ in FLS, which is catalytically essential. The QM regions incorporated the following atom counts and total charges: 116 and 0 for FLS with 2OG, 115 and 0 for FLS with succinate, 123 and 0 for DAH with 2OG, 122 and 0 for DAH with succinate and an equatorial-oxo, and 122 and 0 for DAH with succinate and an axial-oxo.

## Supplementary Materials

### This PDF file includes:

Figs. S1 to S37 Tables S1 to S19 References
