# Supplementary material for: Tracing the stepwise Darwinian evolution of a plant halogenase
Source: Sci Adv. 2025 Aug 13;11(33):eadv6898. doi: 10.1126/sciadv.adv6898 (PMC12346261; doi:10.1126/sciadv.adv6898)
Supplement: Supplementary file 1 — Figs. S1 to S37 Tables S1 to S19 References [file sciadv.adv6898_sm.pdf]

Science Advances
AAAS

Supplementary Materials for

Tracing the stepwise Darwinian evolution of a plant halogenase

Colin Y. Kim *et al.*

Corresponding author: Jing-Ke Weng, jingke.weng@northeastern.edu

Sci. Adv. **11**, eadv6898 (2025)
DOI: 10.1126/sciadv.adv6898

This PDF file includes:

Figs. S1 to S37
Tables S1 to S19
References

a

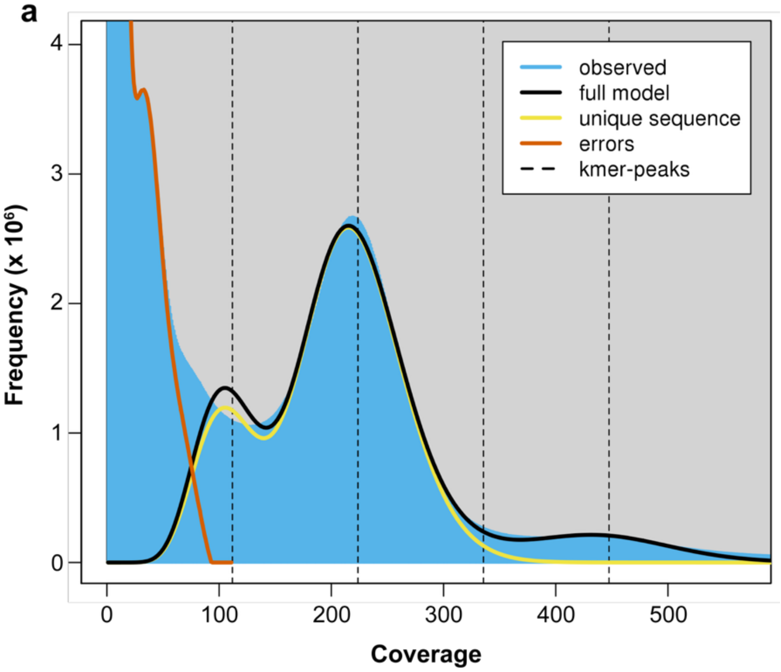

b

Property	Minimum	Maximum
Homozygous	99.19%	99.25%
Heterozygous	0.75%	0.81%
Haploid Length	952,797,334 bp	957,637,840 bp
Repeat Length	635,882,029 bp	639,112,508 bp
Unique Length	316,915,304 bp	318,525,332 bp
Model Fit	38.73%	94.64%
Read Error Rate	0.29%	0.29%

Fig. S1.

Genome size estimation by *k*-mer analysis (*k* = 19). (**a**) Linear plot of model fit output from GenomeScope (*47*). (**b**) Estimations and results collected from GenomeScope analysis.

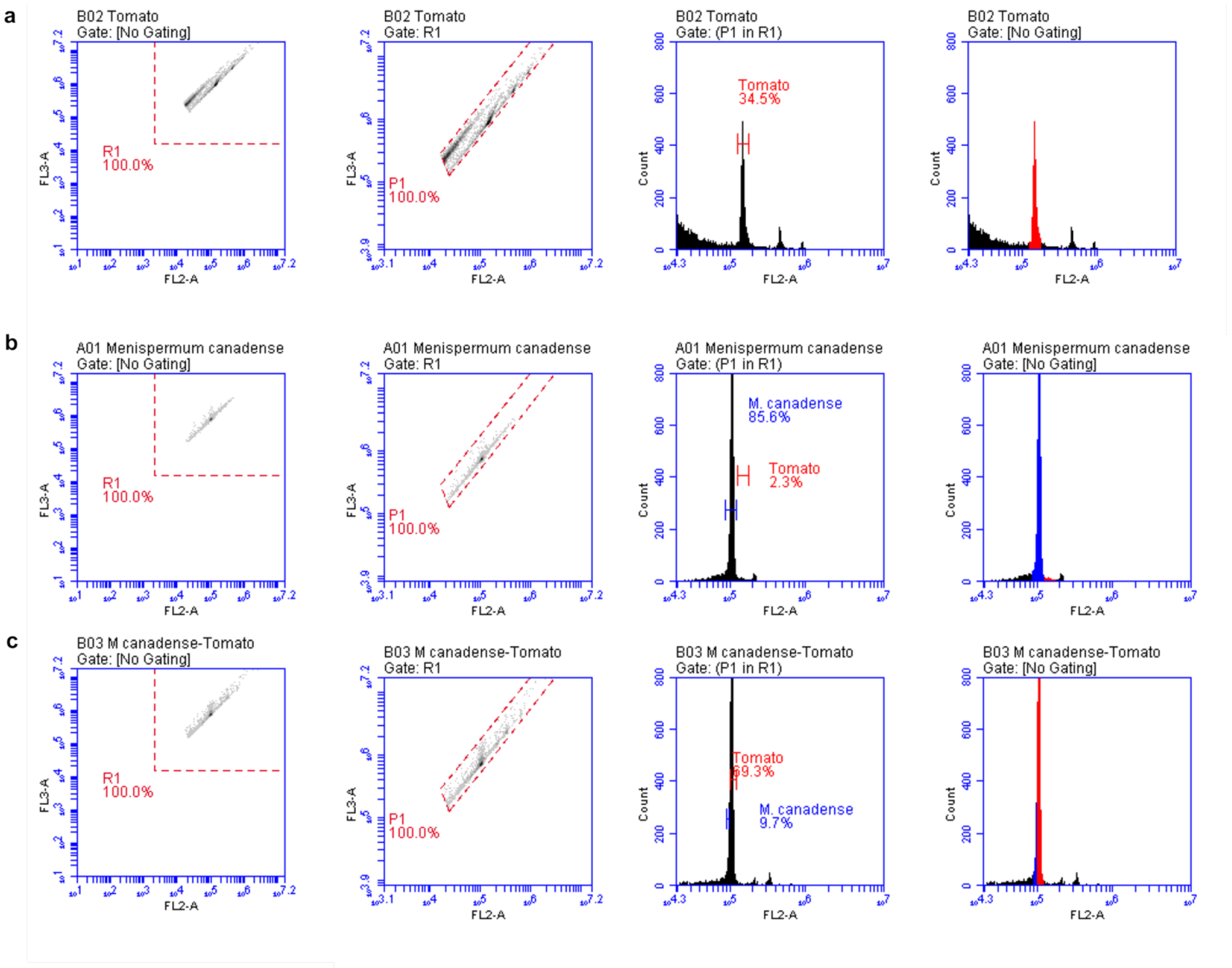

Fig. S2.

Genome size estimation by cell flow-cytometry (BD Accuri™ C6 Cytometer). Flow cytometry measurements for (**a**) *Solanum lycopersicum* (tomato), (**b**) *M. canadense*, and (**c**) *M. canadense* and tomato. The estimated genome size of tomato is 2.05 Gb (2n DNA content)(*106*).

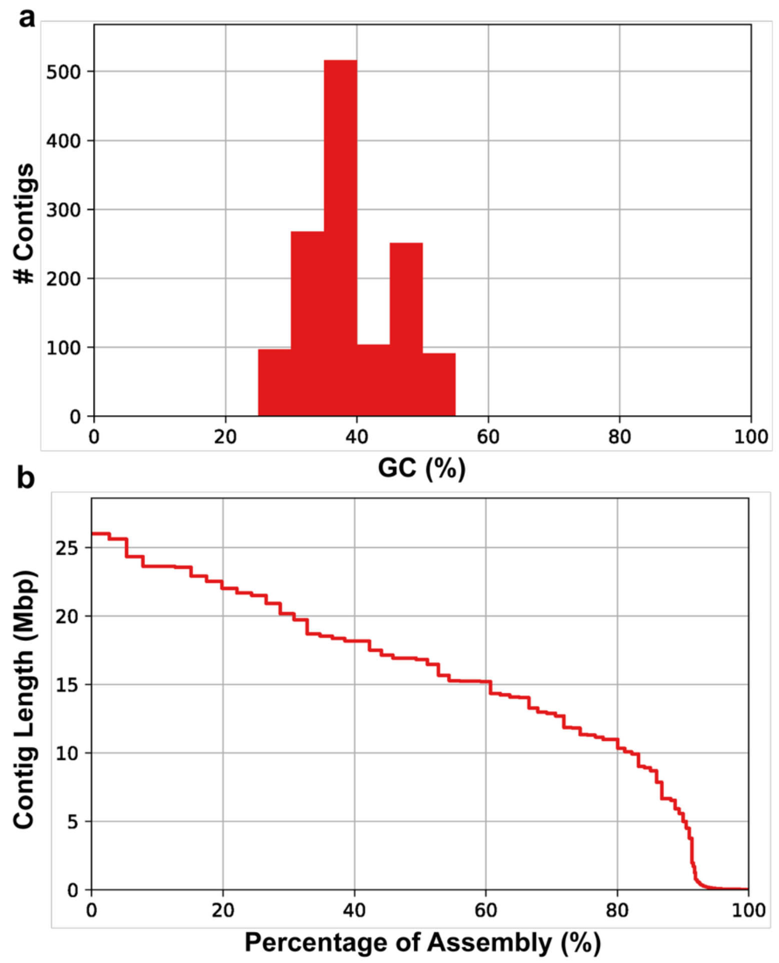

c

Property	Primary Assembly
Number of Contigs	1327
Total Length	969,900,284 bp
N50	16,822,813 bp
N90	4,999,881 bp
auN	15,601,294 bp
L50	24
L90	58
GC(%)	35.10%

Fig. S3.
Genome assembly statistics for initial contig-level assembly. (**a**) GC% of initial assembly (**b**) Contig length (N_x) of genome assembly (**c**) Genome assembly statistics.

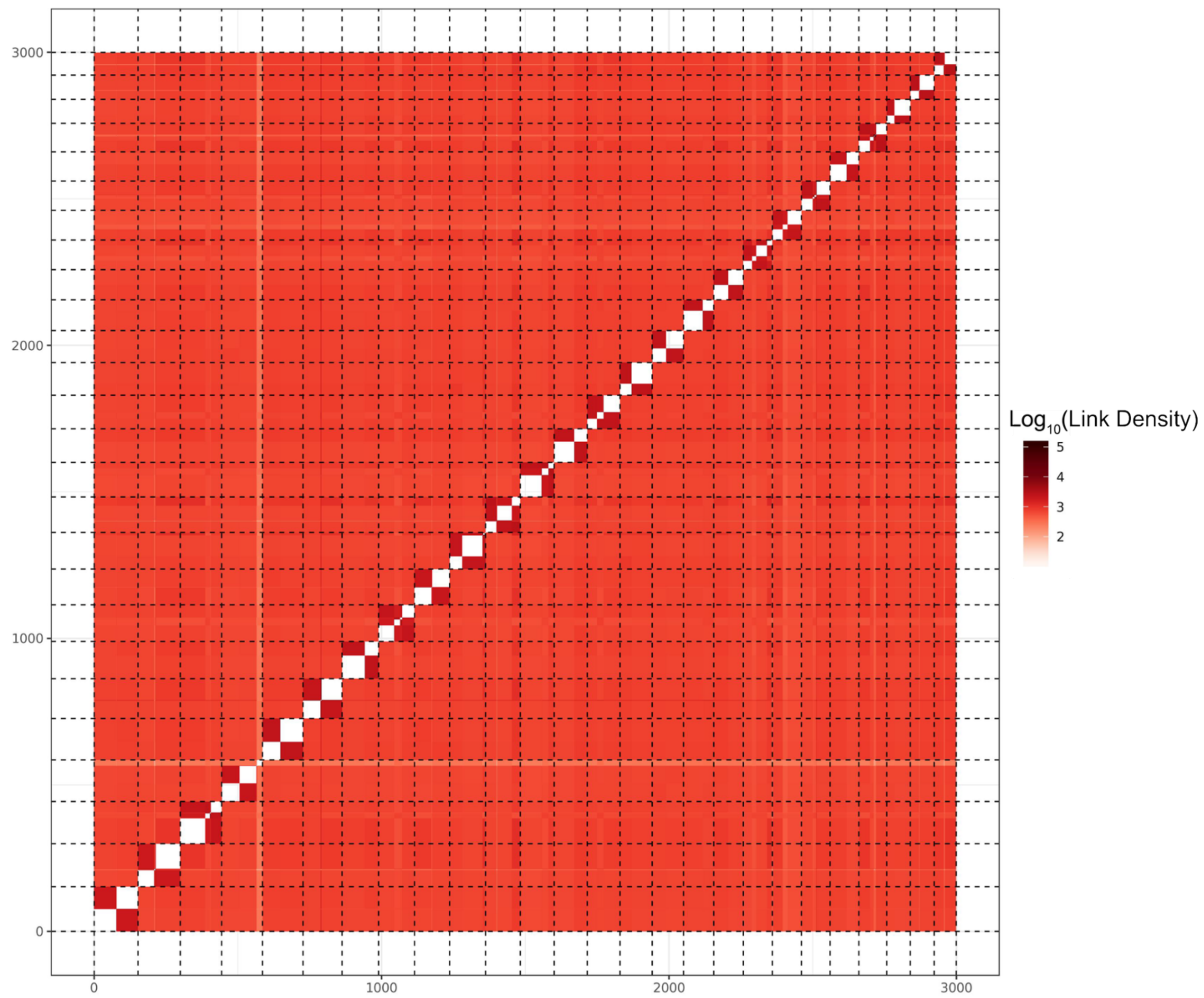

Fig. S4.

Scaffolding of *M. canadense* genome using Hi-C information. Contact map showcasing the $\log_{10}$ of link density between *M. canadense* genome to itself with chunk size of 301,424 bp.

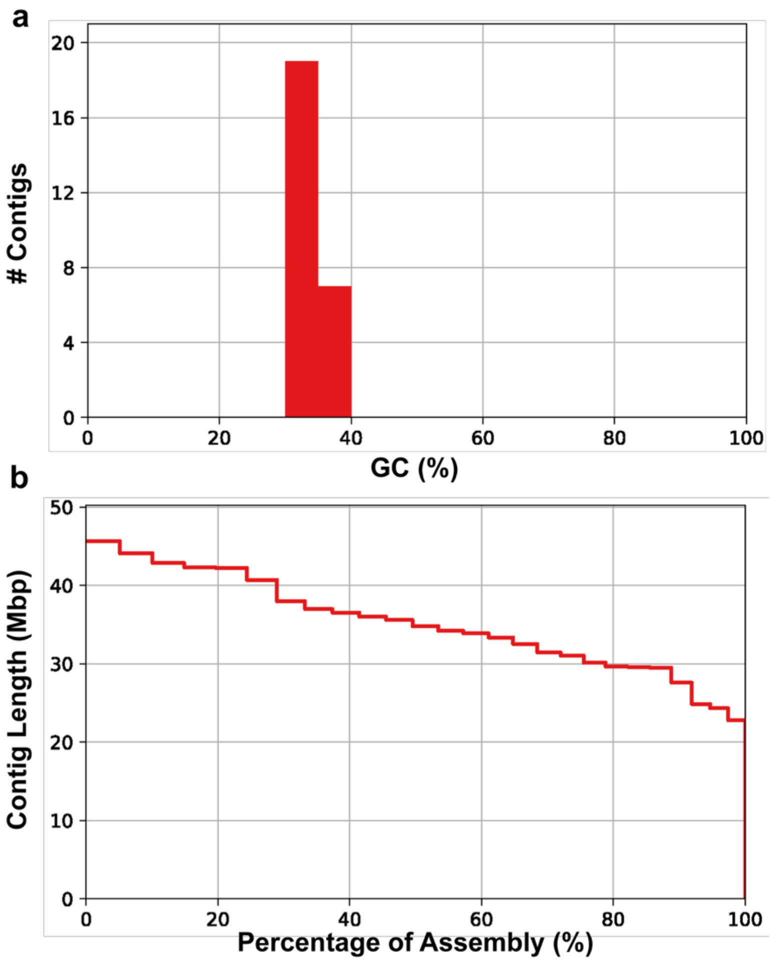

c

Property	Scaffolded Assembly
Number of Contigs	26
Total Length	890,548,923 bp
N50	34,818,747 bp
N90	27,578,300 bp
auN	35,340,600 bp
L50	12
L90	23
GC(%)	34.92%

Fig. S5.

Genome assembly statistics for post-scaffolded chromosomal-level assembly of *M. canadense*. (**a**) GC% of scaffolded assembly (**b**) Contig length (N_x) of genome assembly (**c**) Genome assembly statistics.

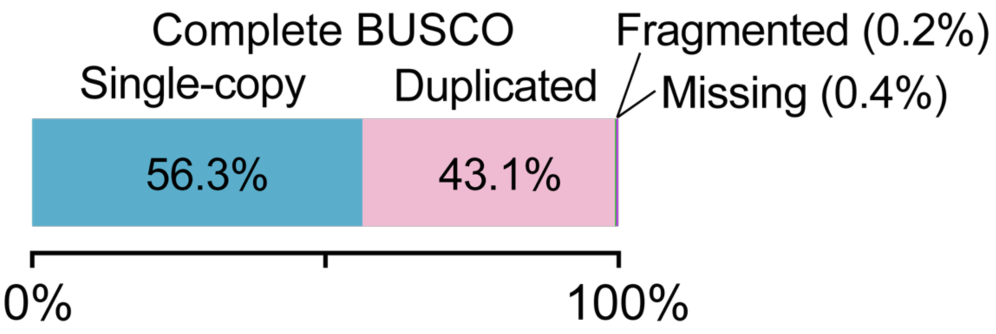

Total BUSCO Groups Searched	1614
Complete BUSCOs	1604 (99.4%)
Complete & Single-Copy BUSCOs	908 (56.3%)
Complete & Duplicated BUSCOs	696 (43.1%)
Fragmented BUSCOs	3 (0.2%)
Missing BUSCOs	7 (0.4%)

Fig. S6.
Benchmarking Universal Single-Copy Orthologs (BUSCO) assessment of *M. canadense* chromosomal-level assembly. The lineage dataset used for BUSCO assessment consists of 1614 genes in 50 embryophyta genomes (embryophyta_odb_10). 43.1% of the completed BUSCO genes exist as duplicates, which is a similar observation found in the *P. somniferum* genome (62%) that went under a relatively recent WGD (*107*).

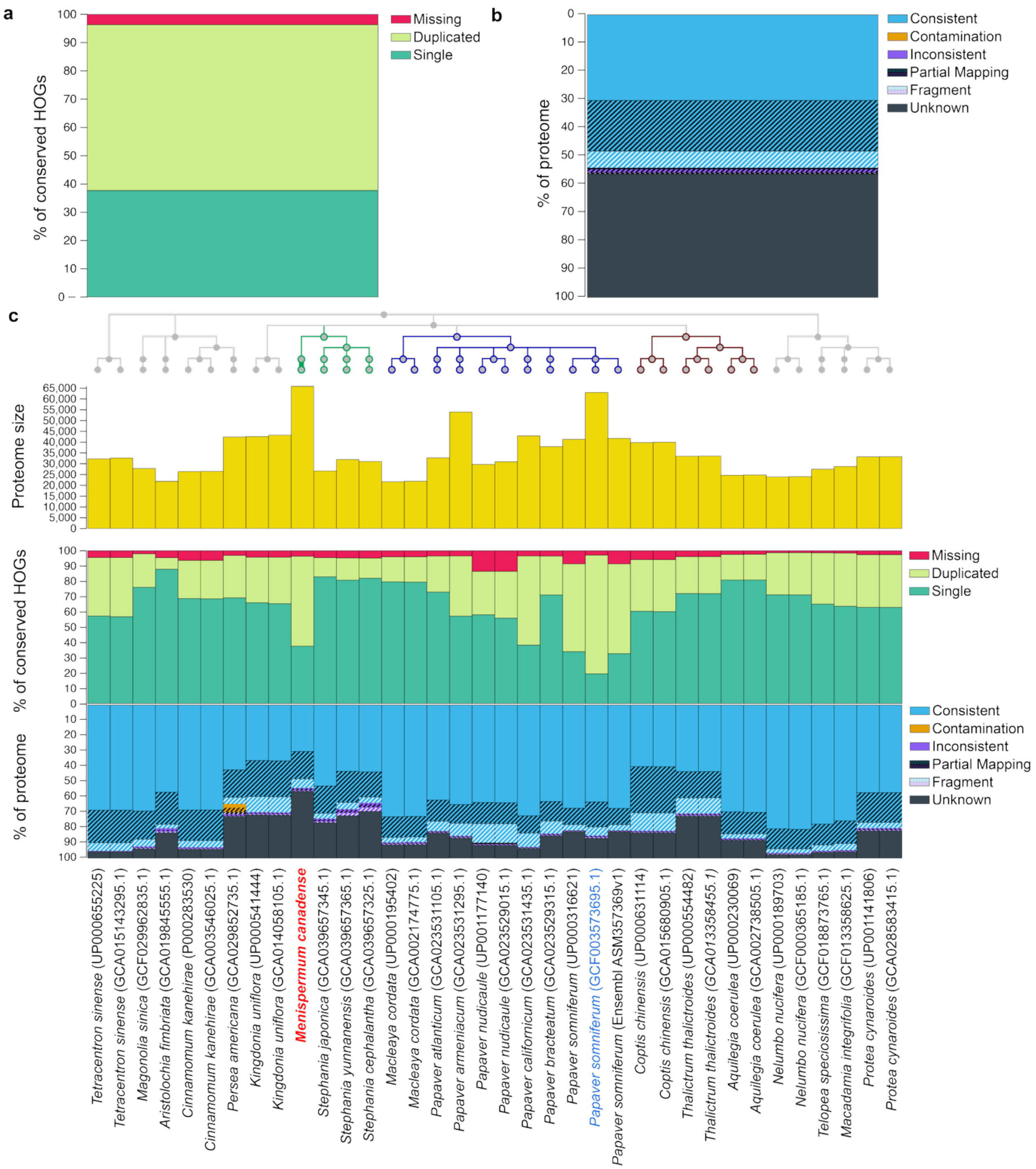

Fig S7.

OMArk comparison of *M. canadense* proteome to the expected gene repertoire of its common ancestor. (**a**) Completeness statistic showing the percent of conserved Hierarchical Orthologous Groups (HOGs) that are represented as single (light green) or duplicated genes (teal), or entirely missing (red) in *M. canadense* proteome. (**b**) Whole proteome assessment of gene model consistency with known homologs present in comparison to gene contents of all extant species of the same lineage to *M. canadense*. Proportion of genes whose closest gene families are from the selected lineage

(consistent; blue), from another lineage as contamination (contamination; orange), from another lineage as noise (inconsistent; purple), and with no closest homologs found (unknown; grey). Partial mapping refers to genes that have less than 80% of the sequence with shared k-mer content from its closest gene family. Fragments indicate genes with a length less than half the median gene content of its closest gene family. There is 58.67% duplication (4768 out of 8127) of HOGs in *M. canadense* proteome, similar to its duplicated BUSCOs in Supplementary Fig. 6. (**c**) Comparison of *M. canadense* proteome statistics with other publicly available Mesangiospermae (core angiosperm) species. *M. canadense* and *P. somniferum* proteomes have relatively high duplicated HOGs. Green branches: Menispermaceae, blue branches: Papaveraceae, brown: Ranunculaceae.

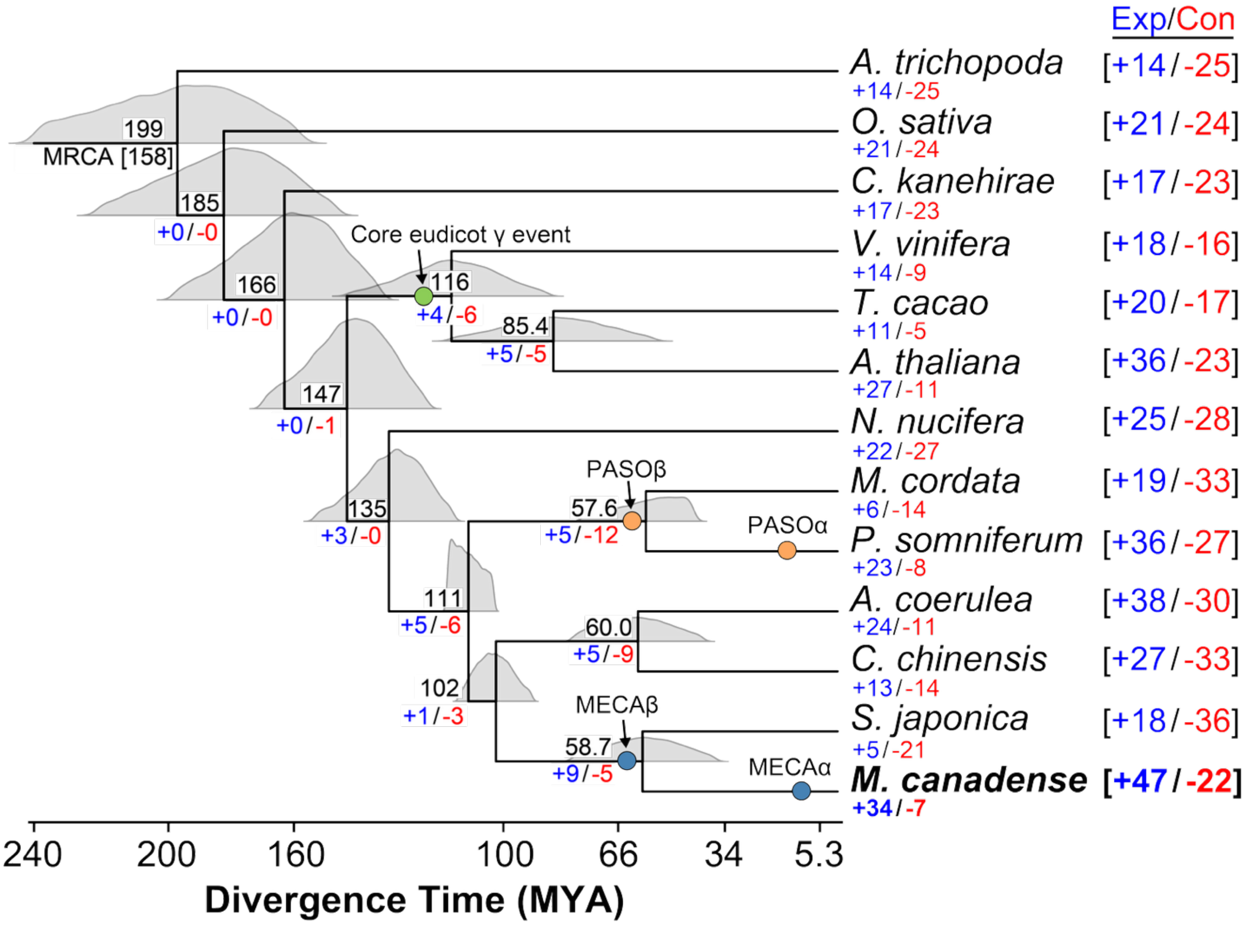

Fig. S8.

2ODD-specific gene family expansion and contraction. Phylogenomic divergence time of *M. canadense* compared to 12 other angiosperm species as shown in Fig. 1c. Black number at the top of each tree node indicates the mean divergence time million years ago (MYA). The blue and red numbers at the bottom of each tree node indicate expansion and contraction, respectively, of 158 gene families with functional annotations to 2ODD. Green circle indicates the relative timing of the core eudicot γ event, whereas orange circles correspond to two WGD events present in *P. somniferum*. Blue circles indicate the relative timing of two predicted WGD events in *M. canadense*.

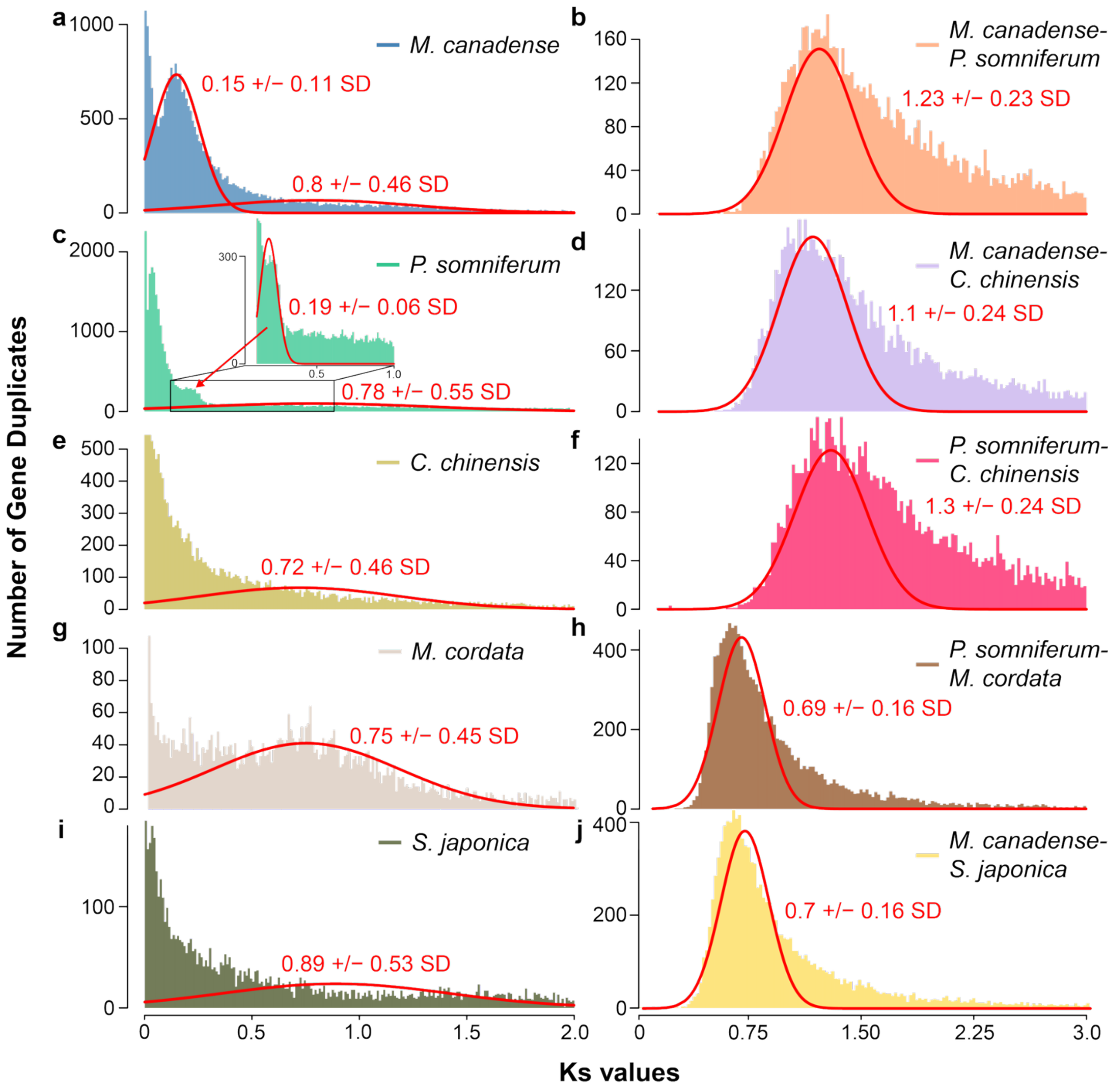

Fig. S9.

Mixtools analyses on distribution of the RBH paralogous gene pair K_S values for *M. canadense*, *P. somniferum*, *C. chinensis*, *M. cordata*, and *S. japonica*. K_S distribution peaks determined by Mixtools analyses using 1000 bootstraps for (**a**) *M. canadense* paralogs, (**b**) *M. canadense* and *P. somniferum* orthologs (**c**) *P. somniferum* paralogs, (**d**) *M. canadense* and *C. chinensis* orthologs, (**e**) *C. chinensis* paralogs, (**f**) *P. somniferum* and *C. chinensis* orthologs, (**g**) *M. cordata* paralogs, (**h**) *P. somniferum* and *M. cordata* orthologs, (**i**) *S. japonica* paralogs, and (**j**) *M. canadense* and *S. japonica* orthologs.

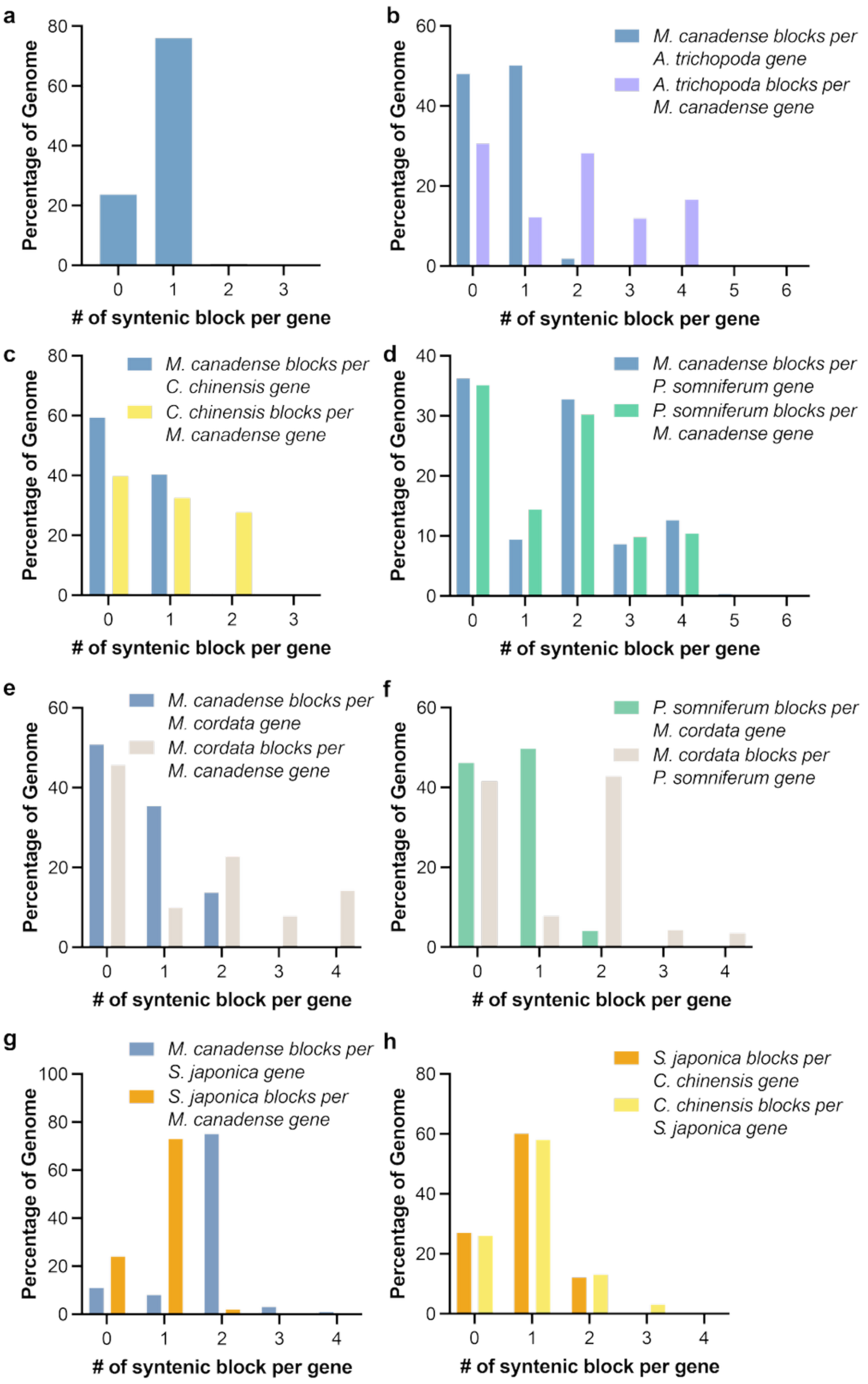

Fig. S10.

The syntenic depth between *M. canadense* and select plant genomes. (**a**) *M. canadense* vs *M. canadense* syntenic depths show 1:1 pattern. (**b**) *M. canadense* vs *A. trichopoda* syntenic depths show 4:1 pattern. (**c**) *M. canadense* vs *C. chinensis* syntenic depths show 2:1 pattern. (**d**) *M. canadense* vs *P. somniferum* syntenic depths show 4:4 pattern. (**e**) *M. canadense* vs *M. cordata* syntenic depths show 4:2 pattern. (**f**) *P. somniferum* vs *M. cordata* syntenic depths show 2:1 pattern. (**g**) *M. canadense* vs *S. japonica* syntenic depths show 2:1 pattern. (**h**) *S. japonica* vs *C. chinensis* syntenic depths show 2:2 pattern.

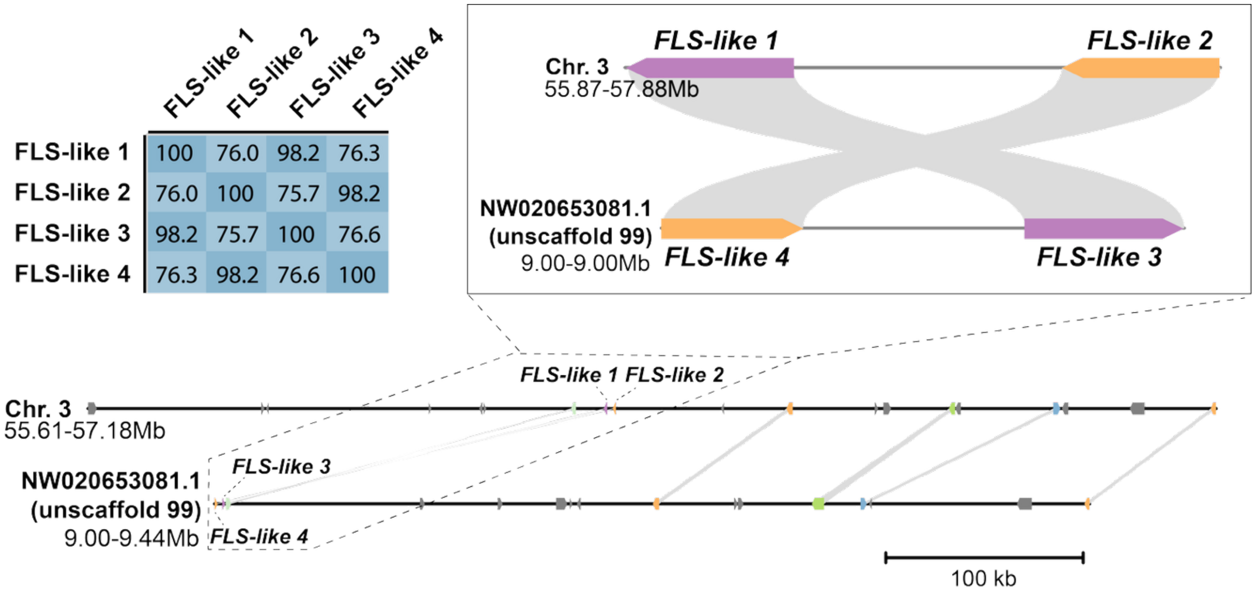

Fig. S11.

Microsynteny analysis of *FLS-like* gene locus in *P. somniferum*. WGD region in chromosome 3 and unscaffold 99 in *P. somniferum* genome containing paralogous tandem duplicated *FLS-like* genes. Amino-acid sequence identity matrix of *FLS-like 1*, *FLS-like 2*, *FLS-like 3*, and *FLS-like 4*, calculated by local pairwise alignments using EMBOSS supermatcher (*45*). The corresponding gene name for each are as *FLS-like 1*; *LOC113346476*, *FLS-like 2*; *LOC113346474*, *FLS-like 3*; *LOC113356203*, and *FLS-like 4*; *LOC113356202* from *P. somniferum* genome (NCBI, GCF003573695).

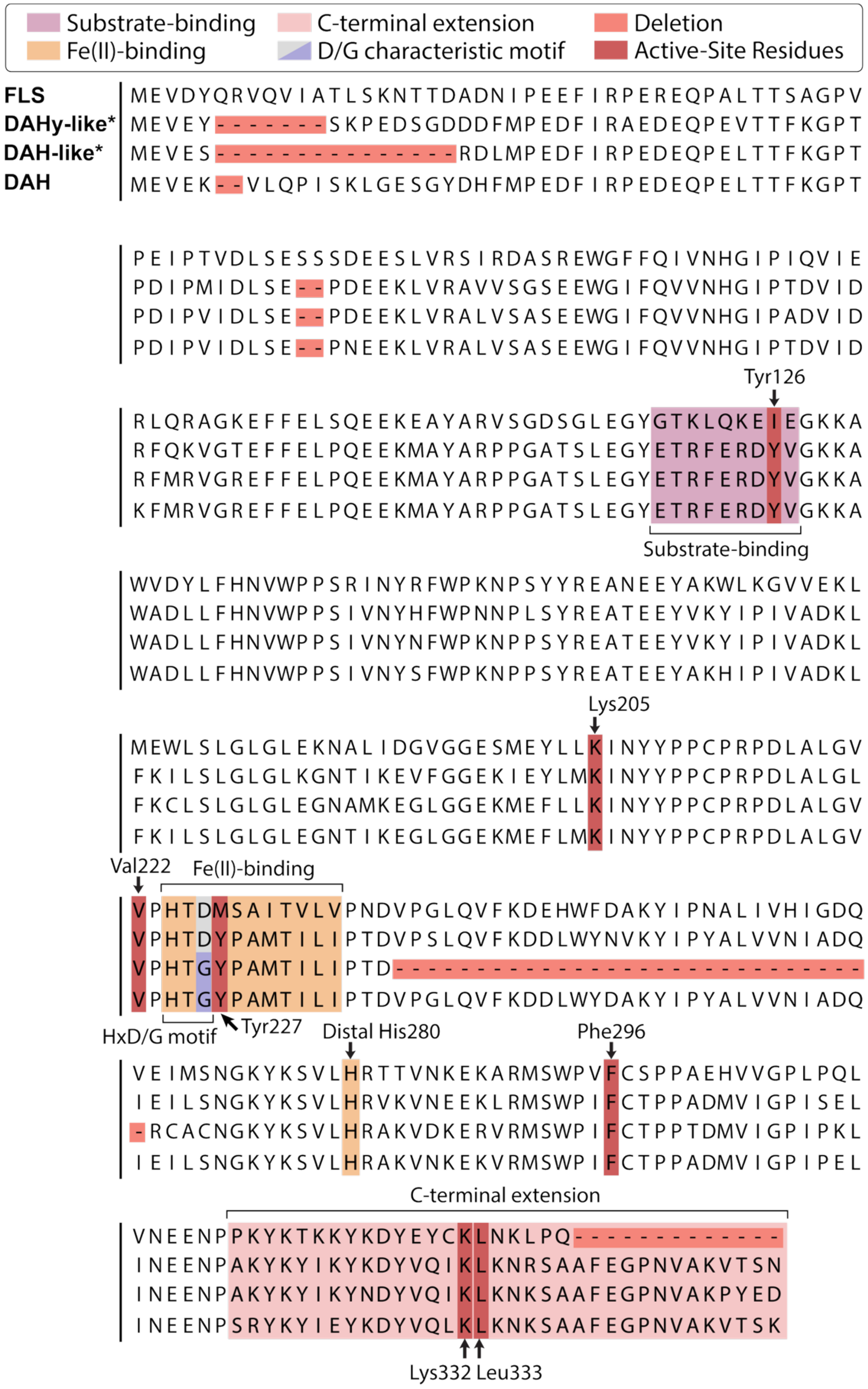

Fig. S12.

Multiple sequence alignment of FLS and DAH paralogs found in chromosomes 2 and 3. Relevant residues and motifs mentioned in this manuscript are highlighted according to the color legend and labels.

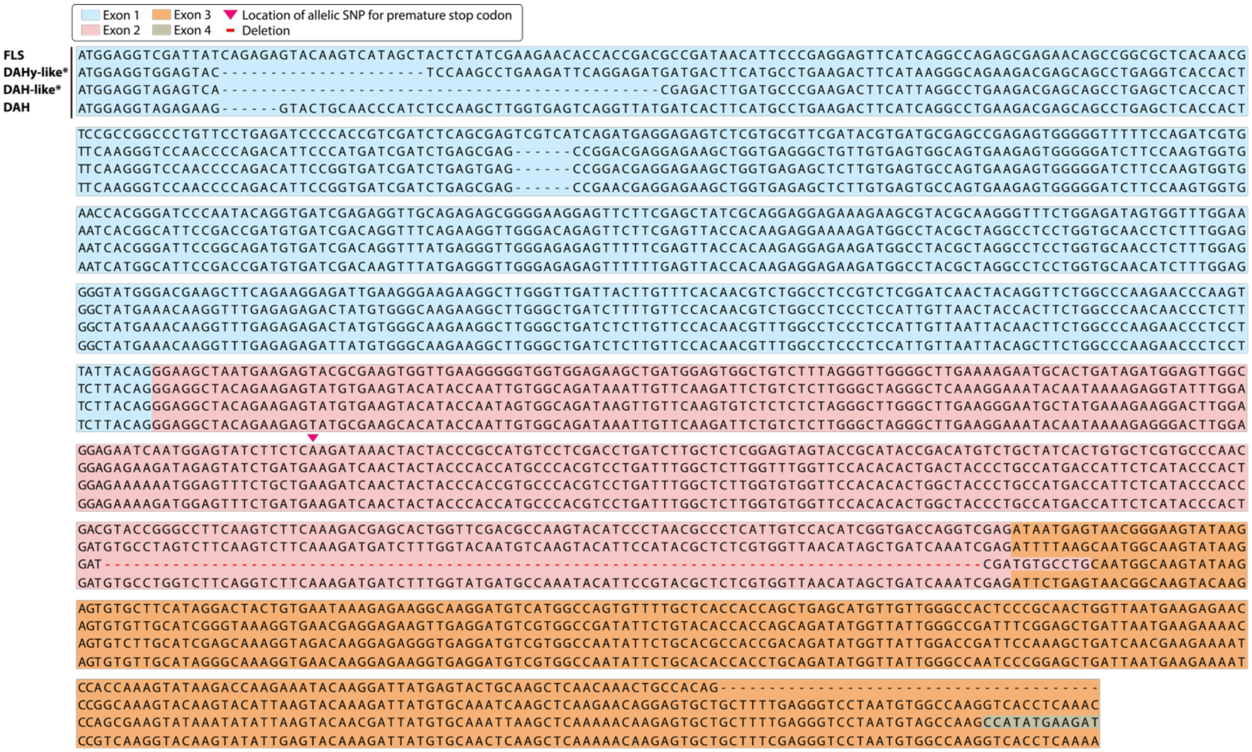

Fig. S13.
Multiple sequence codon alignment of *FLS* and *DAH* paralogous genes found in chromosomes 2 and 3. Relevant residues and motifs mentioned in this manuscript are highlighted according to the color legend and labels.

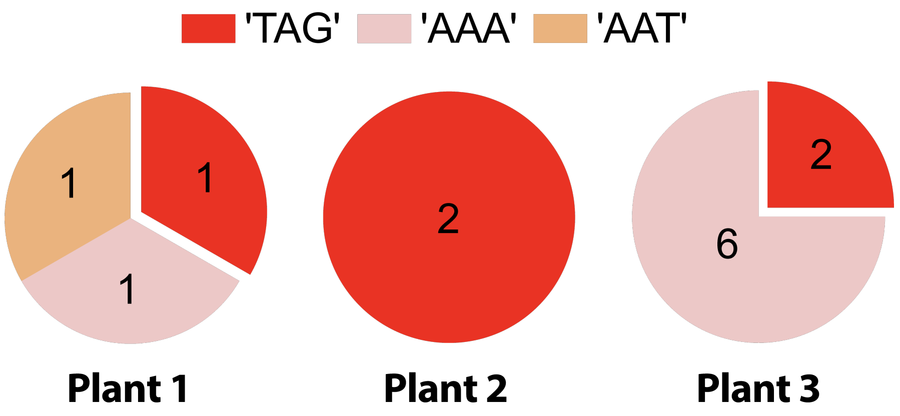

Fig. S14.
Total count of RNA-seq mapped reads in *DAHy-like* Lys200 codon. Quantification of mapped sequences for independent *M. canadense* plant RNA-sequencing samples.

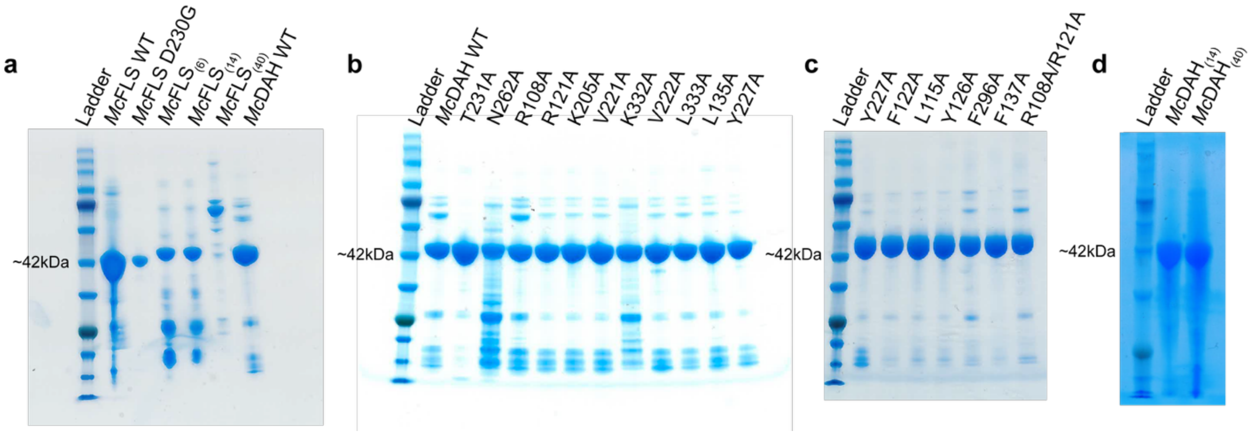

Fig. S15.
SDS-PAGE of recombinant proteins discussed in this study. (**a**) FLS-to-DAH swap mutant proteins. (**b** and **c**) DAH site-directed alanine mutants. (**d**) DAH-to-FLS swap mutant proteins.

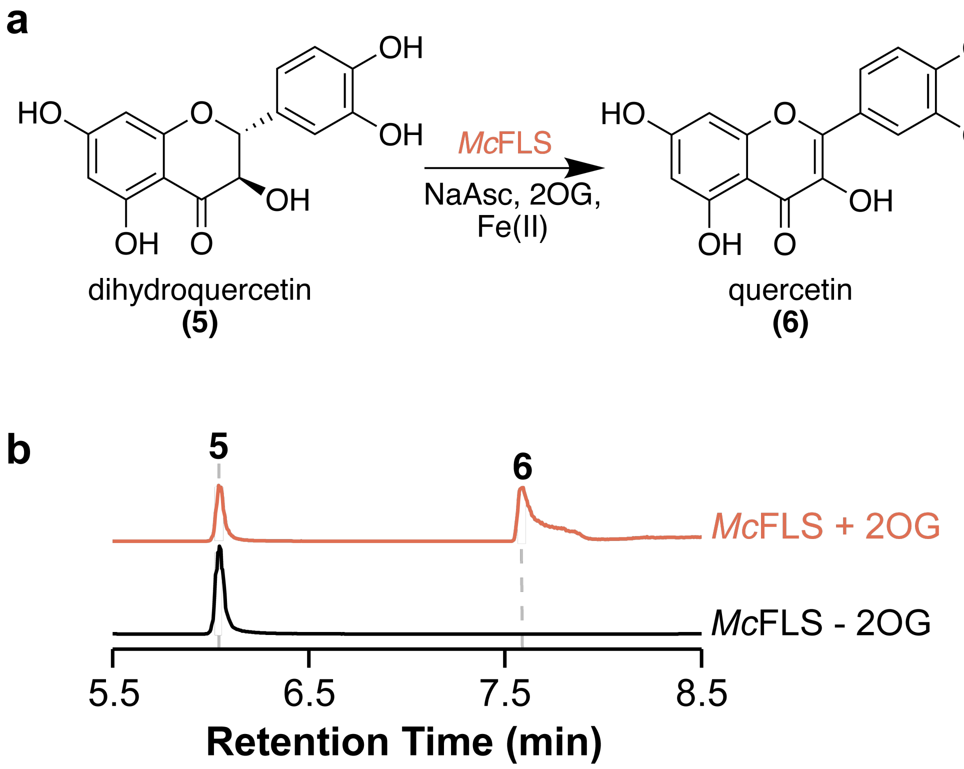

Fig. S16.
***In vitro* biochemical assay of FLS against dihydroquercetin.** (**a**) Reaction schematic of the conversion of dihydroquercetin; **5** to quercetin; **6** using flavonol synthase (*Mc*FLS). (**b**) Combined LC-MS extracted ion chromatograms (EICs) of 303.05081 *m/z*; **5** = $[M-H]^-$ and 301.03528 *m/z;* **6** = $[M-H]^-$. XICs show the in vitro activity of *Mc*FLS that desaturate **5** to **6** in a 2OG-dependent manner.

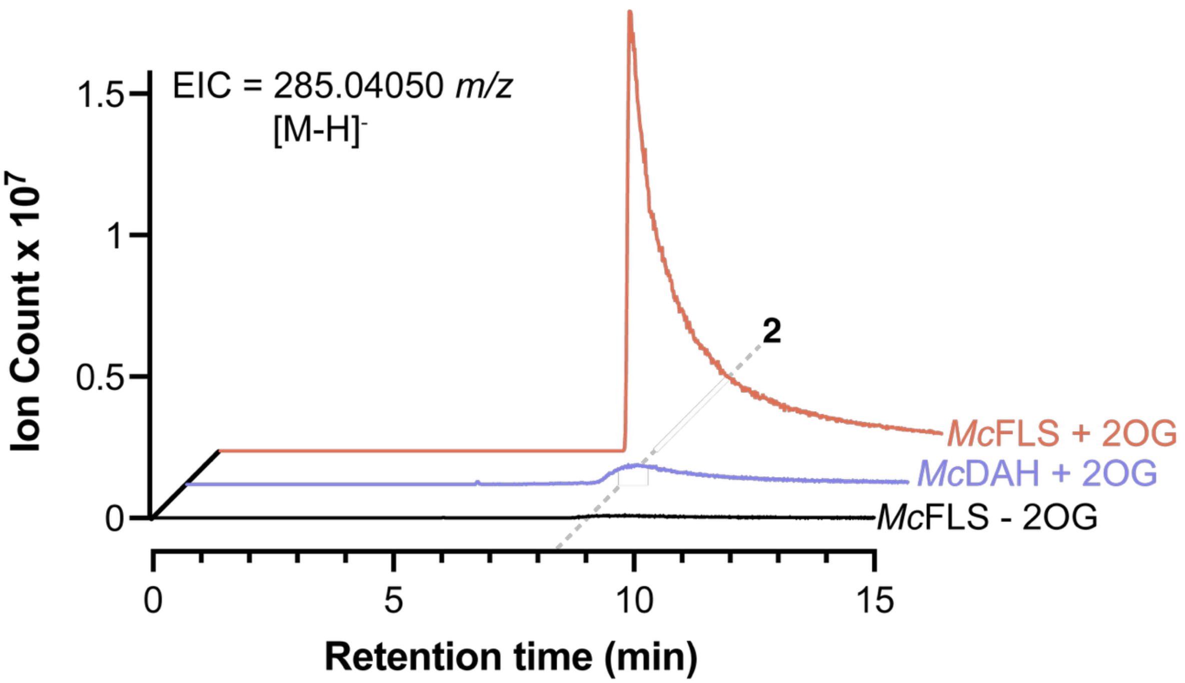

Fig. S17.

Production of trace amounts of kaempferol in *Mc*DAH assay against dihydrokaempferol. LC-HRAM-MS extracted ion chromatogram (EIC) of 285.04050 *m/z*; **2** = $[M\text{-}H]^-$. *Mc*DAH produces trace amounts of **2** in the reaction as indicated by a small peak in *Mc*DAH + 2OG sample trace compared to *Mc*FLS - 2OG sample.

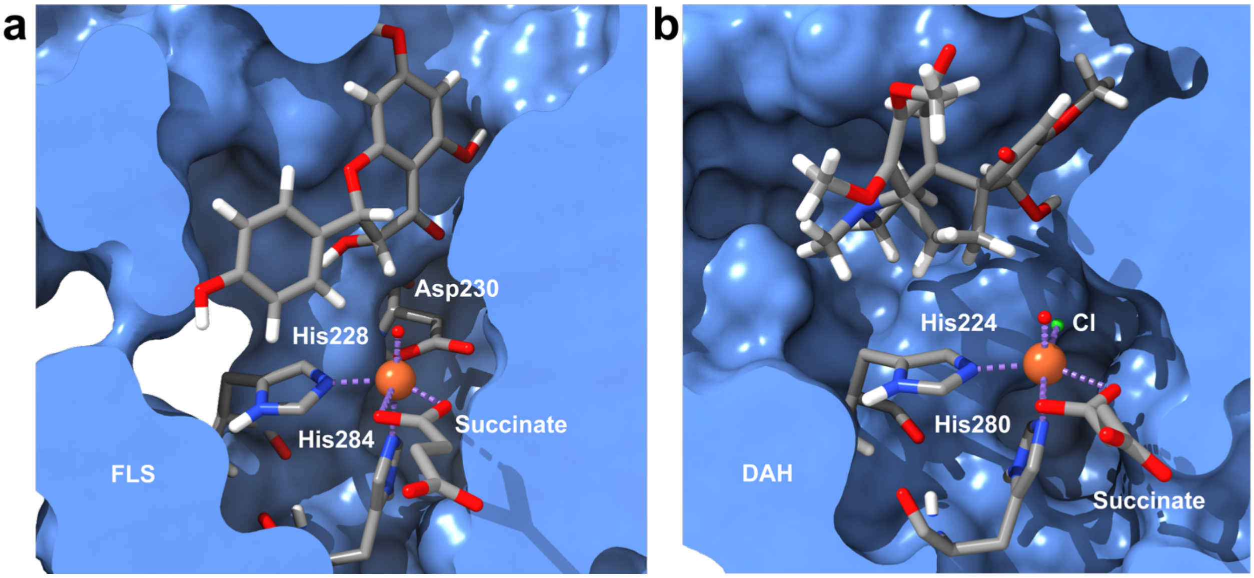

Fig. S18.

Docked AlphaFold structures of the enzyme-substrate complexes using AutoDock Vina. (**a**) Surface representation of the proposed FLS active site with dihydrokaempferol in its docked conformation. (**b**) Surface representation of the proposed DAH active site with dechloroacutumine in its docked conformation. The modeled succinate and oxo bound structure is included to illustrate the substrate position relative to the active site. The protein surface is shown in light blue, carbons in gray, nitrogens in blue, oxygens in red, hydrogens in white, and iron in orange. Dative bonds are shown as purple dashed lines.

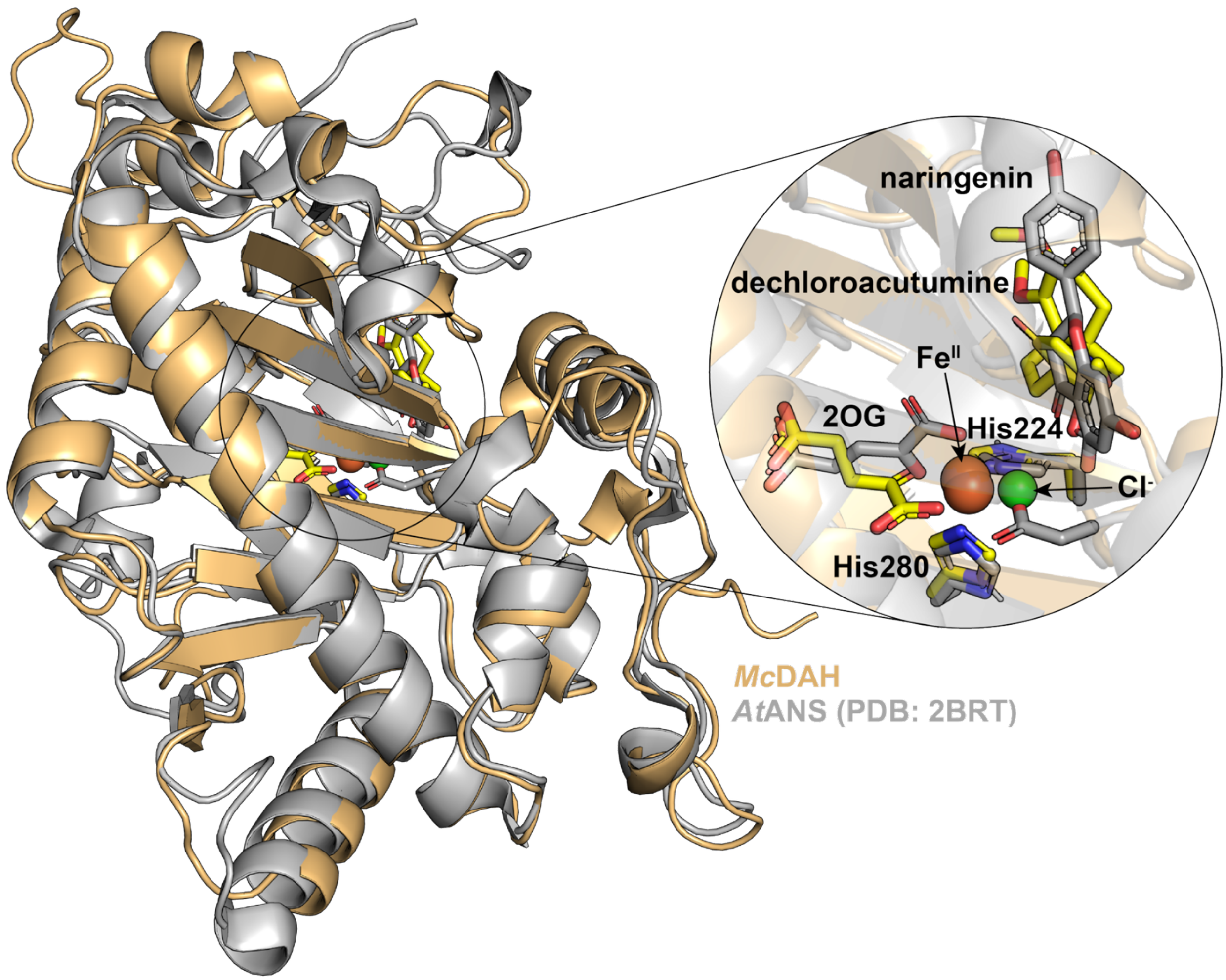

Fig. S19.

Structural alignment of *Mc*DAH AlphaFold structure and *At*ANS. The *McDAH* structure highlighted in gold is aligned to *At*ANS highlighted in gray (PDB: 2BRT). Naringenin, 2OG and Fe(II)-coordinating histidine triad with aspartate from *At*ANS structure are colored in gray, whereas the docked dechloroacutumine and Fe(II)-coordinating histidines from *Mc*DAH are colored in yellow. Fe(II) (orange) and Cl^- anion (green) positions are derived based on this structural alignment.

a

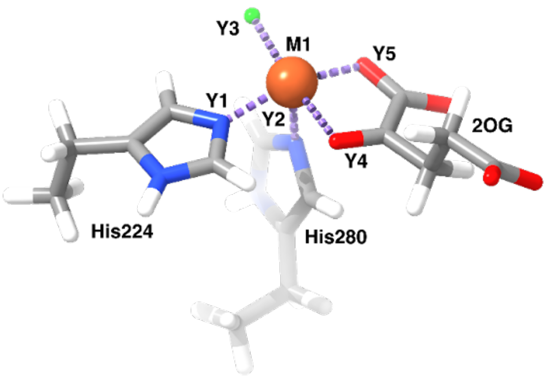

b

Bond	Force constant (kcal/(mol × Å^2))	Equilibrium bond length (Å)
M1-Y3	49.8	2.4006
M1-Y4	28.5	2.2379
M1-Y5	69.7	2.0042
Y1-M1	39.3	2.2036
Y2-M1	47.4	2.1643
**Angle**	**Force constant (kcal/(mol × rad^2))**	**Equilibrium angle (°)**
Y1-M1-Y2	23.72	95.19
Y1-M1-Y3	28.2	94.33
Y1-M1-Y4	50.83	78.29
Y1-M1-Y5	52.59	146.12
Y2-M1-Y3	37.77	101
Y2-M1-Y4	41.96	88.32
Y2-M1-Y5	32.46	104.55
Y4-M1-Y3	45.05	168.68
Y5-M1-Y3	36.2	108.24
Y5-M1-Y4	68.17	75.12

c

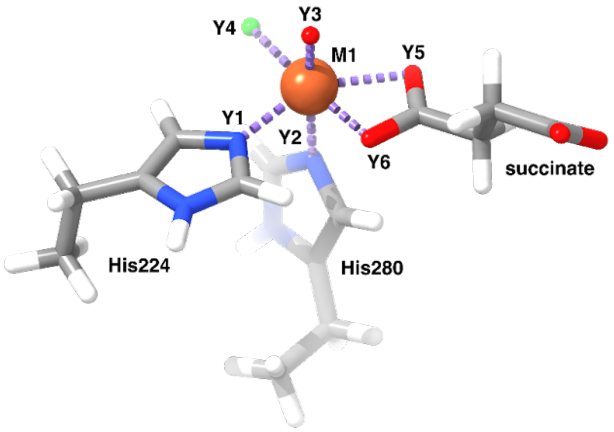

d

Bond	Force constant (kcal/(mol × Å^2))	Equilibrium bond length (Å)
M1-Y3	436	1.6256
M1-Y4	58.8	2.3497
M1-Y5	0	2.5278
M1-Y6	86.1	1.9567
Y1-M1	42.2	2.1899
Y2-M1	42	2.1171
**Angle**	**Force constant (kcal/(mol × rad^2))**	**Equilibrium angle (°)**
Y1-M1-Y2	71.98	88.53
Y1-M1-Y3	50.93	96.35
Y1-M1-Y4	37.21	102.65
Y1-M1-Y5	74.6	140.01
Y1-M1-Y6	73.71	84.64
Y2-M1-Y3	53.57	175.05
Y2-M1-Y4	74.93	87.16
Y2-M1-Y5	54.29	78.31
Y2-M1-Y6	97.49	86.02
Y4-M1-Y3	57.87	92.59
Y5-M1-Y3	51.53	97.31
Y5-M1-Y4	53.92	114.04
Y6-M1-Y3	67.92	93.57
Y6-M1-Y4	82.95	169.88
Y6-M1-Y5	110.82	57.16

e

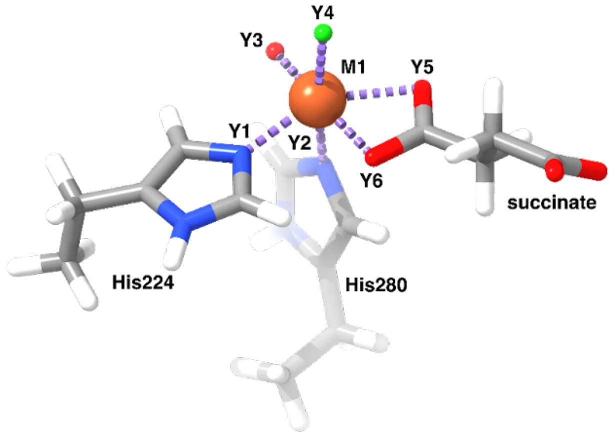

f

Bond	Force constant (kcal/(mol × Å^2))	Equilibrium bond length (Å)
M1-Y3	431.7	1.6352
M1-Y4	61.7	2.3234
M1-Y5	35.7	2.1484
M1-Y6	68.4	2.0215
Y1-M1	36.2	2.2004
Y2-M1	52.7	2.1632
**Angle**	**Force constant (kcal/(mol × rad^2))**	**Equilibrium angle (°)**
Y1-M1-Y2	35.65	86.57
Y1-M1-Y3	61.43	99.68
Y1-M1-Y4	33.49	90.94
Y1-M1-Y5	90.98	147.97
Y1-M1-Y6	95.18	85.89
Y2-M1-Y3	55.75	88.16
Y2-M1-Y4	70.72	176.13
Y2-M1-Y5	33.26	83.8
Y2-M1-Y6	72.05	84.96
Y4-M1-Y3	51.97	95.18
Y5-M1-Y3	83.6	110.43
Y5-M1-Y4	35.08	96.83
Y6-M1-Y3	87.12	170.88
Y6-M1-Y4	68.91	91.92
Y6-M1-Y5	142.76	62.9

Fig. S20.

The core active site of *Mc*DAH structural models from AMBER's Metal Center Parameter Builder (MCPB). DAH with (**a**) 2OG, (**c**) succinate and axial-oxo, or (**e**)

succinate and equatorial-oxo in the MCPB small model with atom assignments labeled on the active site for coordinating atoms. Dative bonds are illustrated with purple dashed lines. Atoms are colored as follows: carbon in gray, nitrogen in blue, oxygen in red, hydrogen in white, and iron in orange. Parameters generated from DAH with (**b**) 2OG, (**d**) succinate and axial-oxo, or (**e**) succinate and equatorial-oxo force field parameters assigned using the metal center parameter builder (MCPB.py) from AmberTools22. Only the equilibrium bond lengths, angles, and their force constants that are directly bonded to the metal center are reported. Parameters were generated using the Seminario method.

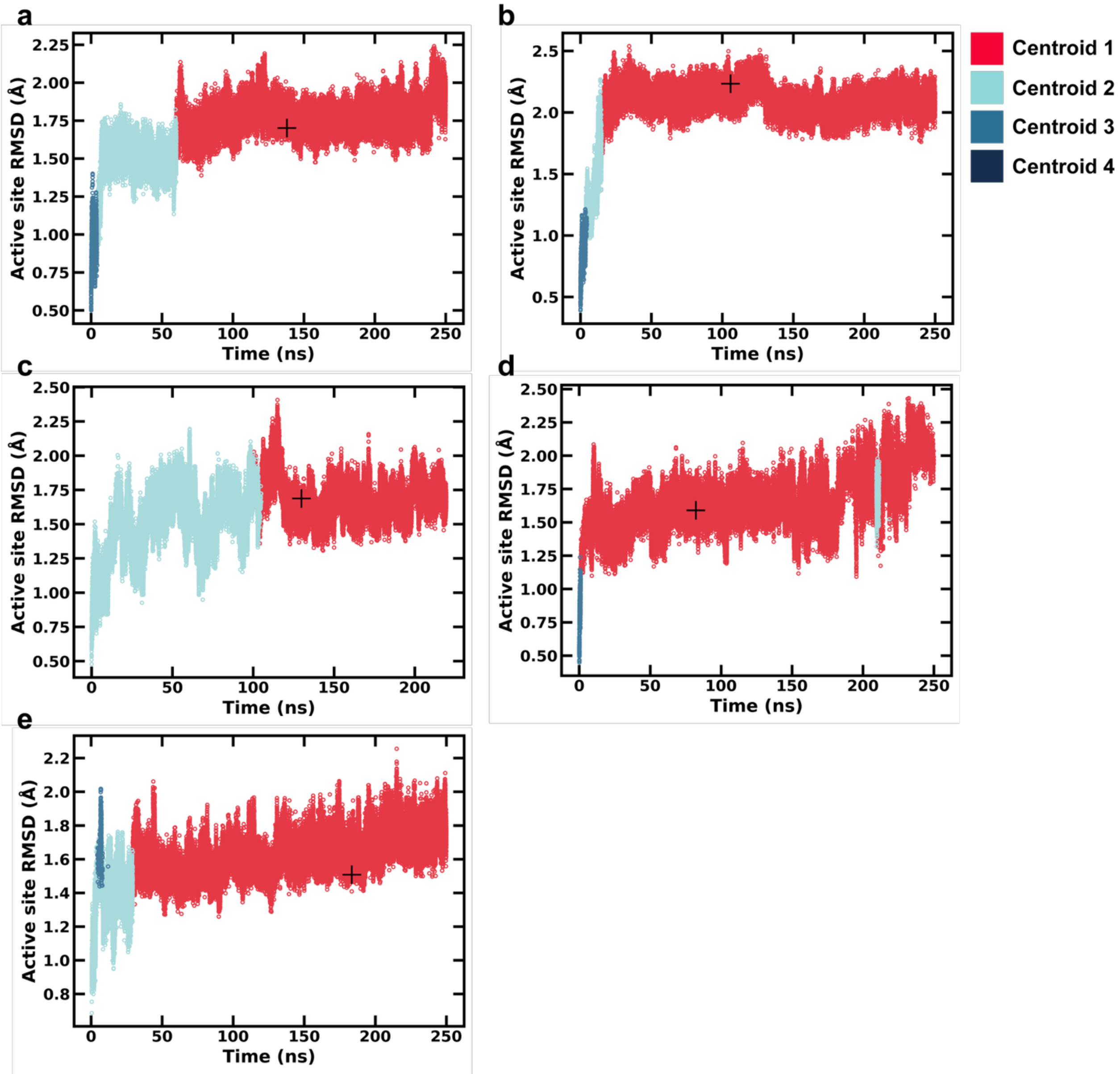

Fig. S21.

Clustered restrained MD simulations for DAH. The results of 250 ns MD simulations represented as time (ns) vs root mean squared deviation (Å) to the first frame. The simulations were clustered using CPPTraj and the DBSCAN method. (**a**) DAH 2OG simulation. Default clustering parameters were used with minpoints = 25 and = 0.75. (**b**) DAH succinate equatorial-oxo unrestrained simulation. Default clustering parameters were used with minpoints = 25 and = 0.76. (**c**) DAH succinate equatorial-oxo obtuse simulation. Default clustering parameters were used with minpoints = 25 and = 0.64. (**d**) DAH succinate axial-oxo unrestrained simulation. Default clustering parameters were used with minpoints = 25 and = 0.68. (**e**) DAH succinate axial-oxo obtuse simulation. Default clustering parameters were used with minpoints = 25 and = 0.66. The centroid of the largest cluster is marked with a crosshair.

a

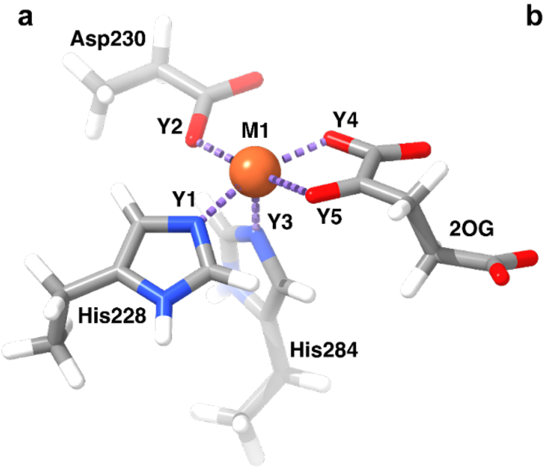

b

Bond	Force constant (kcal/(mol × Å²))	Equilibrium bond length (Å)
M1-Y4	81.3	1.9842
M1-Y5	27.6	2.2314
Y1-M1	32.5	2.2506
Y2-M1	18.1	2.1937
Y3-M1	31.7	2.2397
**Angle**	**Force constant (kcal/(mol × rad²))**	**Equilibrium angle (°)**
Y1-M1-Y2	34.5	89.3
Y1-M1-Y3	36.96	88.84
Y1-M1-Y4	48.7	157.02
Y1-M1-Y5	48.09	80.6
Y2-M1-Y3	55.49	89.22
Y2-M1-Y4	52.71	113.41
Y2-M1-Y5	70.67	168.8
Y3-M1-Y4	25.93	94.65
Y3-M1-Y5	35.2	95.37
Y5-M1-Y4	73.57	76.46

c

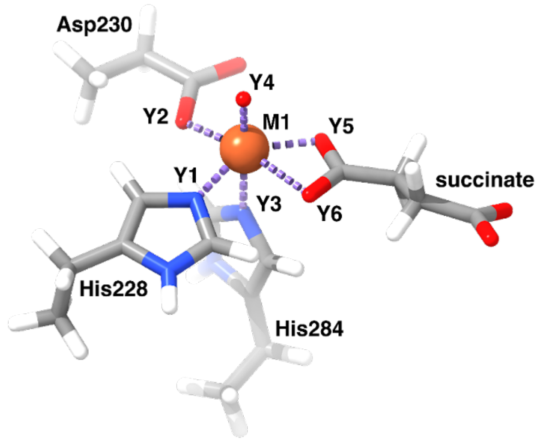

d

Bond	Force constant (kcal/(mol × Å²))	Equilibrium bond length (Å)
M1-Y4	438.8	1.6277
M1-Y5	0	2.55
M1-Y6	90.4	1.9394
Y1-M1	40.8	2.1916
Y2-M1	107	1.9007
Y3-M1	45.1	2.1366
**Angle**	**Force constant (kcal/(mol × rad²))**	**Equilibrium angle (°)**
Y1-M1-Y2	39.48	99.78
Y1-M1-Y3	56.29	86.96
Y1-M1-Y4	47.59	97.48
Y1-M1-Y5	80.64	140.66
Y1-M1-Y6	85.35	84.78
Y2-M1-Y3	84.77	83.15
Y2-M1-Y4	69.79	95.38
Y2-M1-Y5	58.28	116.23
Y2-M1-Y6	106.45	168.34
Y3-M1-Y4	58.71	175.51
Y3-M1-Y5	55.7	82.39
Y3-M1-Y6	88.15	86.4
Y5-M1-Y4	53.13	94.51
Y6-M1-Y4	67.64	94.65
Y6-M1-Y5	117.3	56.92

Fig. S22.

The core active site of *Mc*FLS structural models from AMBER's Metal Center Parameter Builder (MCPB). FLS with (**a**) 2OG or (**c**) succinate in the MCPB small model with atom assignments labeled on the active site for coordinating atoms. Dative bonds are illustrated with purple dashed lines. Atoms are colored as follows: carbon in gray, nitrogen in blue, oxygen in red, hydrogen in white, and iron in orange. Parameters generated from FLS with (**b**) 2OG or (**d**) succinate force field parameters assigned using the metal center parameter builder (MCPB.py) from AmberTools22. Only the equilibrium bond lengths, angles, and their force constants that are directly bonded to the metal center are reported. Parameters were generated using the Seminario method.

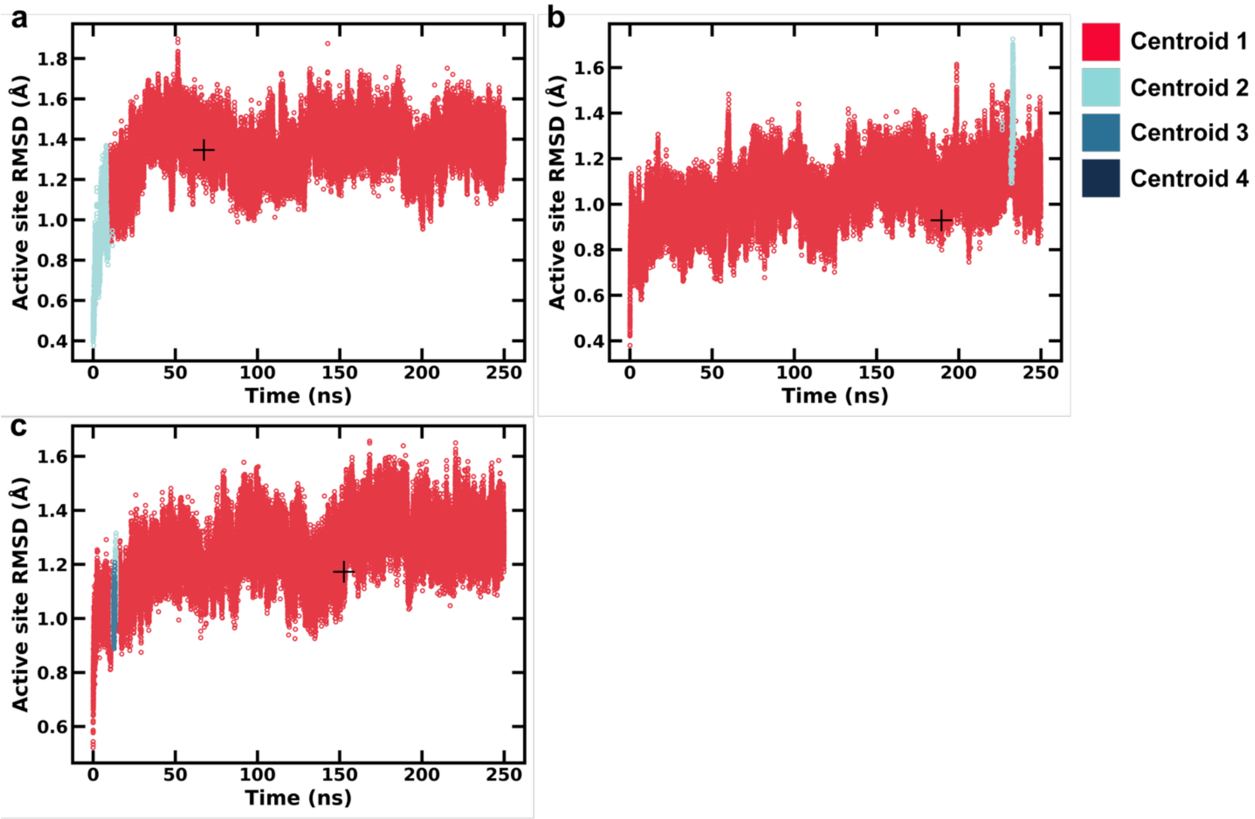

Fig. S23.

Clustered restrained MD simulations for FLS. The results of 250 ns MD simulations represented as time (ns) vs root mean squared deviation (Å) to the first frame. The simulations were clustered using CPPTraj and the DBSCAN method. (**a**) FLS 2OG simulation. Default clustering parameters were used with minpoints = 25 and = 0.62. (**b**) FLS succinate unrestrained simulation. Default clustering parameters were used with minpoints = 25 and = 0.66. (**c**) FLS succinate acute simulation. Default clustering parameters were used with minpoints = 25 and = 0.70. The centroid of the largest cluster is marked with a crosshair.

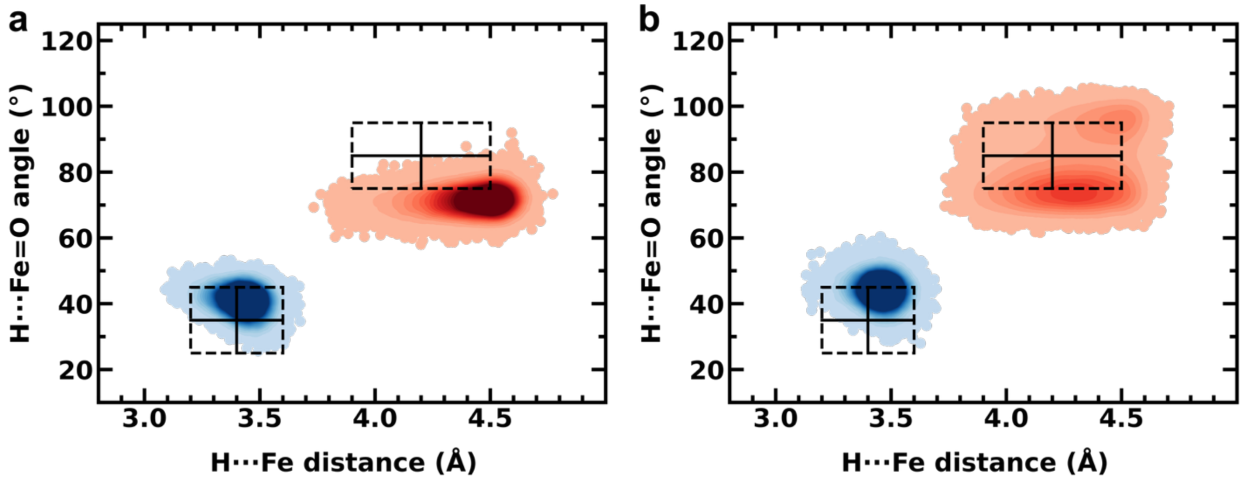

Fig. S24.
Comparison of angle and distance preferences for DAH using spectroscopically guided MD simulations. 250 ns of MD simulations were performed with the oxo in either the (**a**) axial position or (**b**) the equatorial position. The MD simulations were ran with either hydroxylase or halogenase restraints applied with restraints of 100 kcal/(mol · rad^2) for the H···Fe=O angle (°) and 100 kcal/(mol · Å^2) for the H···Fe distance (Å). The H···Fe=O angle and the H···Fe distance were measured with CPPTraj. Experimental HYSCORE data (*38*) was used for hydroxylase-inspired restraints (blue KDE) or halogenase-inspired restraints (red KDE). The experimental target angles for the hydroxylases (OH) and halogenases (Cl) are indicated with a dotted box and crosshairs.

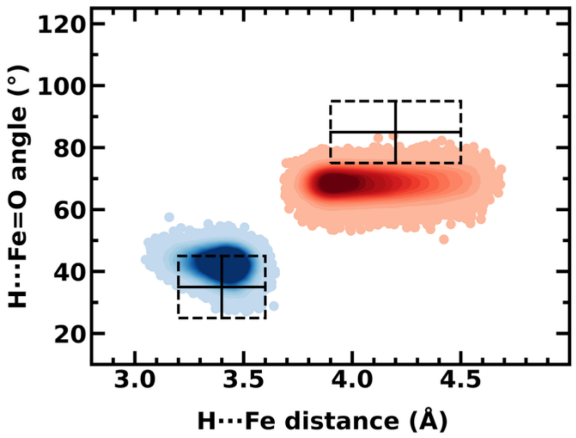

Fig. S25.
Comparison of angle and distance preferences for FLS using spectroscopically guided MD simulations. 250 ns of MD simulations were performed with either hydroxylase or halogenase restraints applied with restraints of 100 kcal/(mol · rad^2) for the H···Fe=O angle (°) and 100 kcal/(mol · Å^2) for the H···Fe distance (Å). The H···Fe=O angle and the H···Fe distance were measured with CPPTraj. Experimental HYSCORE data (*108*) was used for hydroxylase-inspired restraints (blue KDE) or halogenase-inspired restraints (red KDE). The experimental target angles for the hydroxylases (OH) and halogenases (Cl) are indicated with a dotted box and crosshairs.

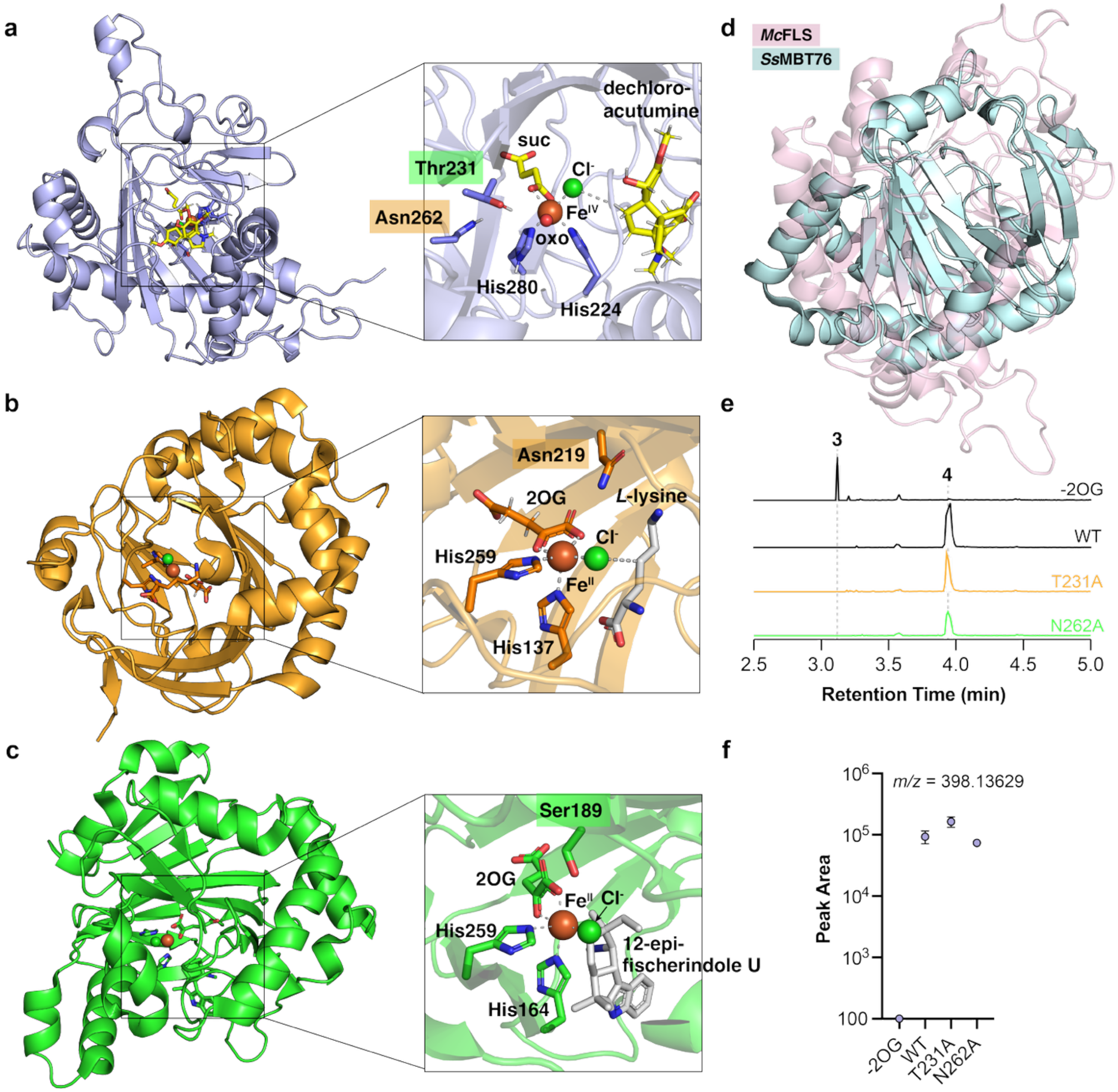

Fig. S26.

Structural and functional comparison between plant and bacterial 2ODHs. Structures of (**a**) plant 2ODH, *Mc*DAH (with the oxo in the equatorial position) compared with bacterial 2ODHs (**b**) *Sc*BesD and (**c**) *Hw*WelO5. Catalytically essential residues in *Sc*BesD (Asn219) and *Hw*WelO5 (Ser189) and their counterpart residues in *Mc*DAH are highlighted. (Abbreviation; suc: succinate, 2OG: 2-oxoglutarate) (**d**) Structural comparison between the QM-MM-optimized structural model of *Mc*FLS generated using constraints that favor an acute oxo-Fe(IV)-H target angle (pink) with the subunit B of *Ss*MBT76 hydroxylase (cyan; PDB: 7JSD). The RMSD of the structural alignment is 23.166. (**e**) Extracted ion chromatograms (EICs) of acutumine, 398.13629 *m/z* ; $[M+H]^+$ of **4** for *Mc*DAH WT enzyme assay performed without and with 2-oxoglutarate (black), *Mc*DAH T231A mutant assay (orange), and *Mc*DAH N262A (green). (**f**) Integrated peak area of **4** from the chromatograms shown in **e**. All assays were performed in triplicates and the error bars represent standard error of the mean.

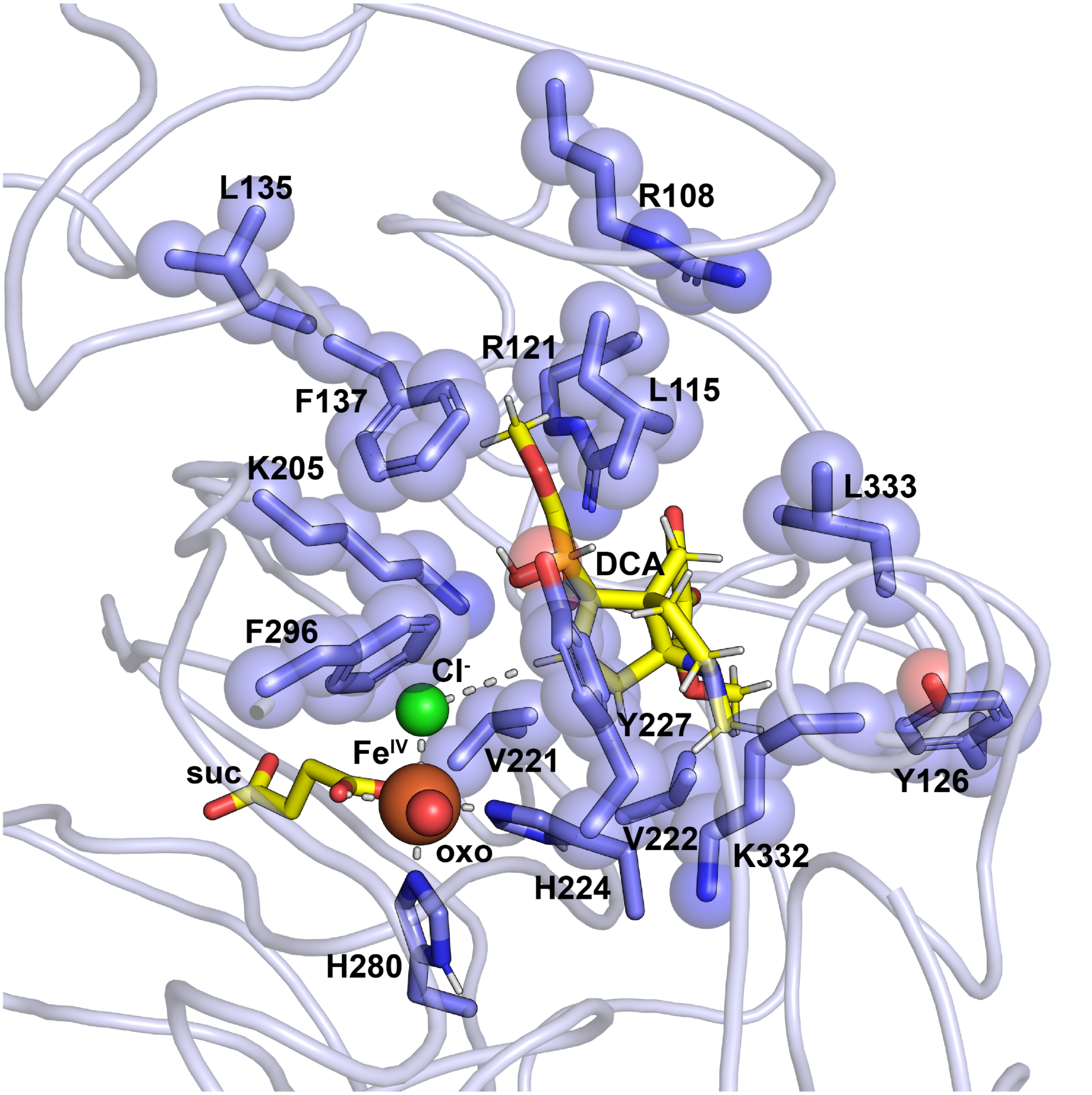

Fig. S27.
Active-site-lining residues around the substrate binding pocket in *Mc*DAH. Relevant residues chosen in this study for alanine scanning portrayed in equatorial-oxo conformation of *Mc*DAH model. (Abbreviation; suc: succinate, DCA: dechloroacutumine)

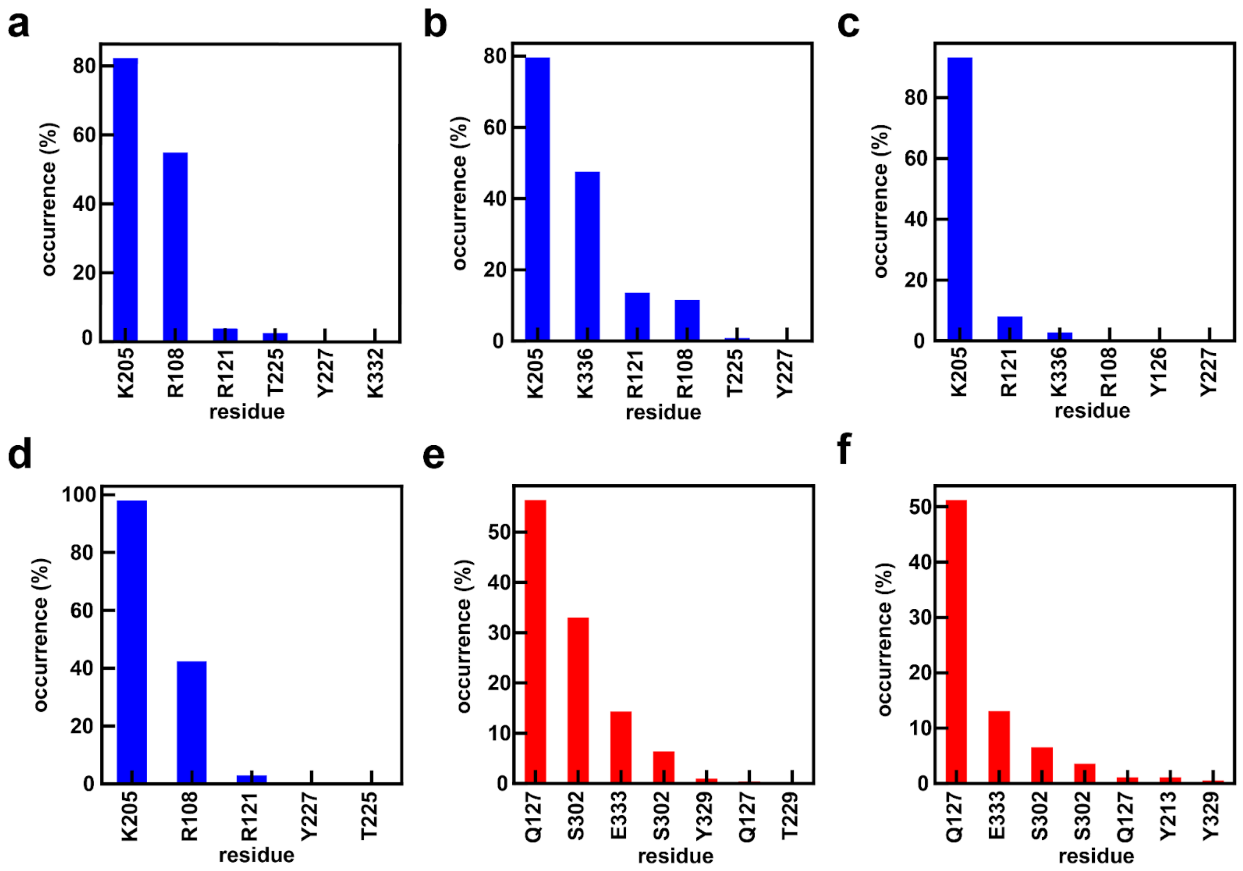

Fig. S28.

Comparison of the hydrogen bonding occurrence (%) summed over any atoms belonging to either interaction partner between DAH residues and dechloroacutumine (blue bars) in the following configurations: (**a**) axial-oxo unrestrained with succinate, (**b**) axial-oxo restrained obtuse with succinate, (**c**) equatorial-oxo unrestrained with succinate, and (**d**) equatorial-oxo restrained obtuse with succinate. Bars in each plot are ordered by decreasing frequency of hydrogen bonding occurrence. Comparison of hydrogen bonding occurrence (%) between FLS residues and dihydrokaempferol (red plots) for the following configurations: (**e**) unrestrained with succinate and (**f**) restrained acute with succinate. For the restrained simulations, harmonic restraints of 100 kcal/(mol·rad^2) were employed for the angle between the oxo, the iron, and the hydrogen atom target. Harmonic restraints of 100 kcal/(mol·Å^2) were used for the distance between iron and the hydrogen atom target. Hydrogen bonding occurrence is obtained based on the default CPPTraj geometric criteria and a modified distance cutoff of 3.2 Å.

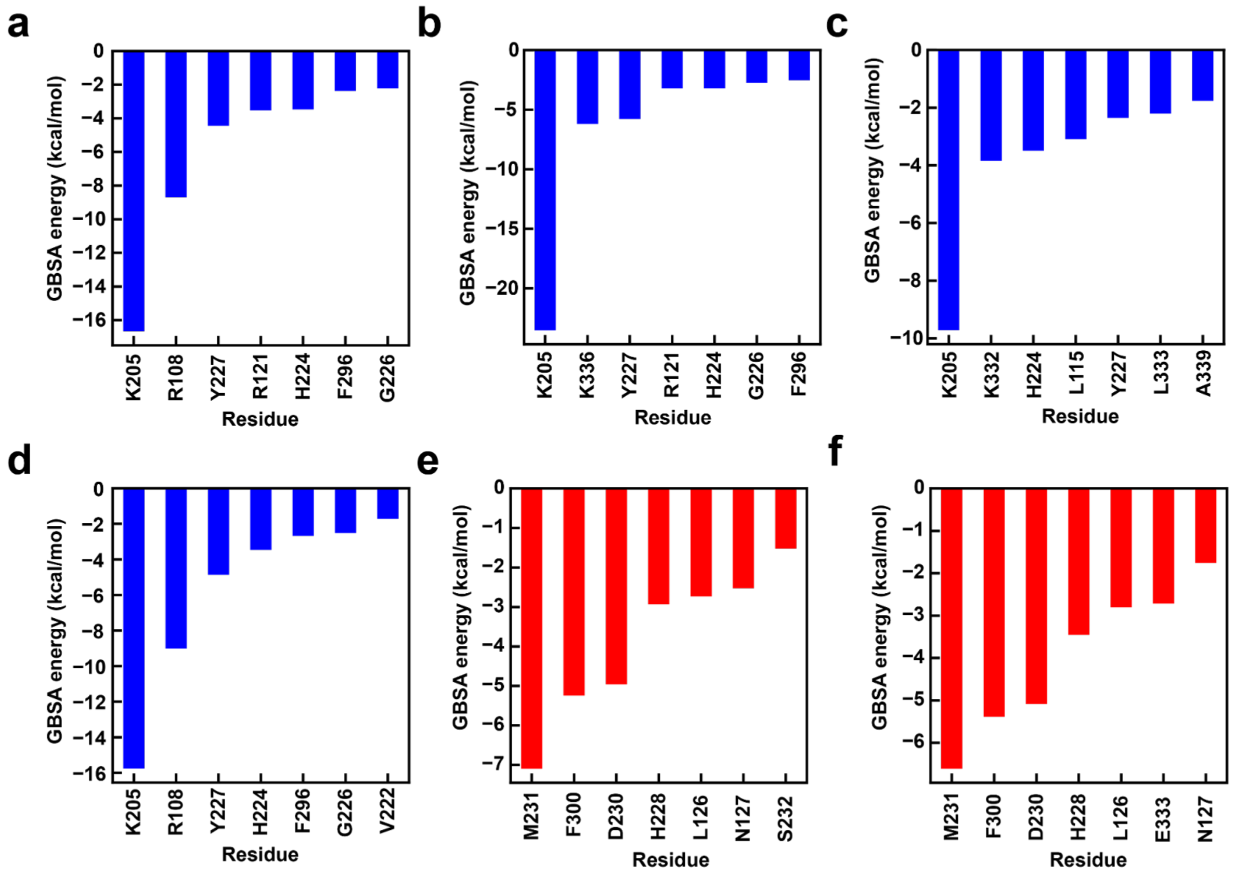

Fig. S29.

Comparison of the Generalized Born surface area (GBSA) analysis in kcal/mol. Contributions to the non-covalent interactions are shown as the energetic sum of the interaction components, vDW, electrostatic, polar, and non-polar, between DAH residues and dechloroacutumine (blue bars) in the following configurations: (**a**) axial-oxo unrestrained with succinate, (**b**) axial-oxo restrained obtuse with succinate, (**c**) equatorial-oxo unrestrained with succinate, (**d**) equatorial-oxo restrained obtuse with succinate. Comparison of GBSA of the classical interactions between FLS residues and dihydrokaempferol (red bars) for the following configurations: (**e**) unrestrained with succinate and (**f**) restrained acute with succinate. GBSA was performed on 1000 snapshots from the primary cluster of DBSCAN-clustered restrained MD simulations. Snapshots were taken 50 ps frames apart as described previously.(*92*) No entropy correction was applied, and the Generalized Born model with the Onufriev, Bashford, and Case (OBC) effective radii set 1 (igb=2) was employed (*109*).

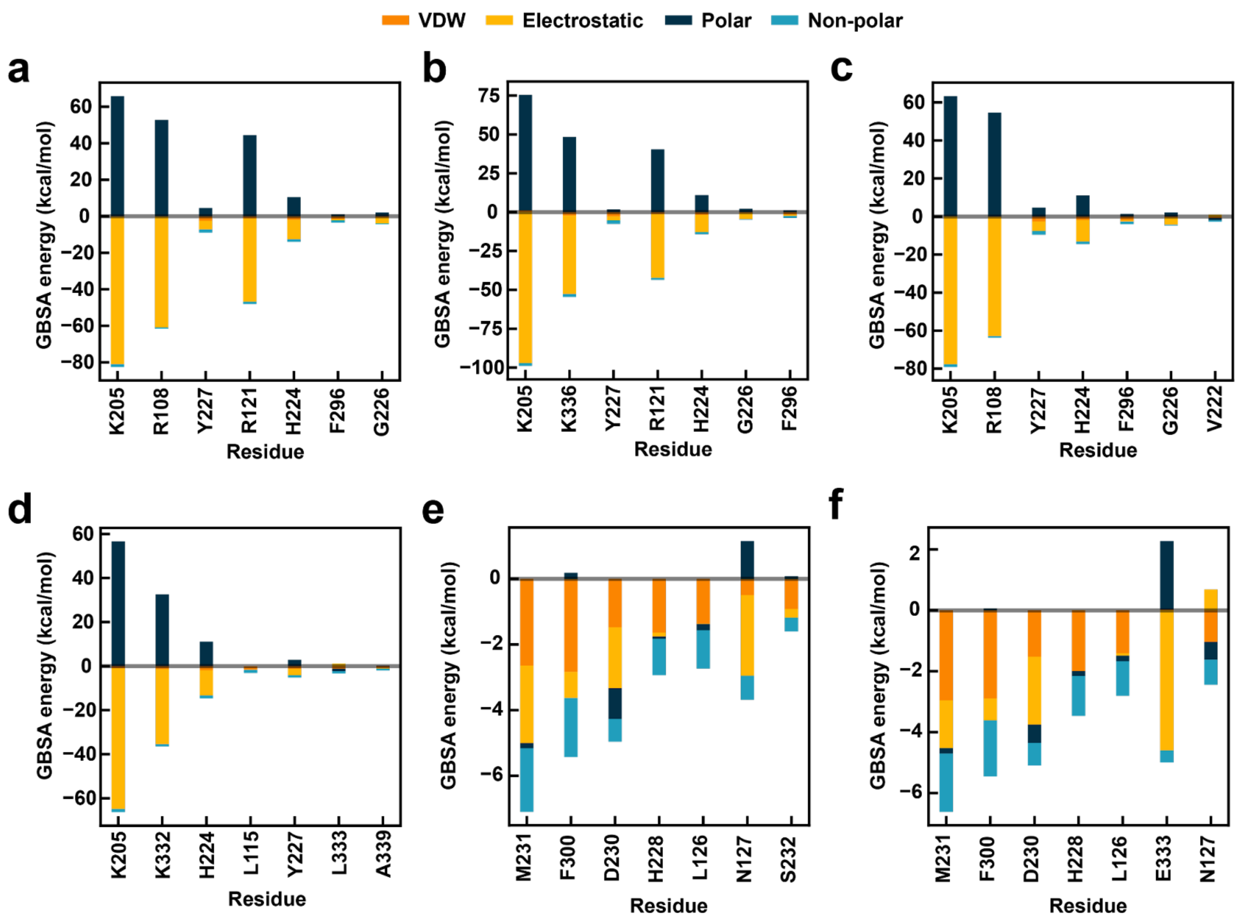

Fig. S30.

Comparison of the GBSA interaction energies (in kcal/mol). Contributions to the non-covalent interactions are broken up by VDW, electrostatic, polar, and non-polar interaction components for the classical interactions between DAH residues and dechloroacutamine in the following configurations: (**a**) axial-oxo unrestrained with succinate, (**b**) axial-oxo restrained obtuse with succinate, (**c**) equatorial-oxo unrestrained with succinate, (**d**) equatorial-oxo restrained obtuse with succinate. Comparison of the GBSA of the classical interactions between FLS residues and dihydrokaempferol for the following configurations: (**e**) unrestrained with succinate and (**f**) restrained acute with succinate. GBSA was performed on 1000 snapshots from the primary cluster of DBSCAN-clustered restrained MD simulations. Snapshots were taken 50 ps frames apart as described previously.(*92*) No entropy correction was applied, and the Generalized Born model with the Onufriev, Bashford, and Case (OBC) effective radii set 1 (igb=2) was employed (*109*).

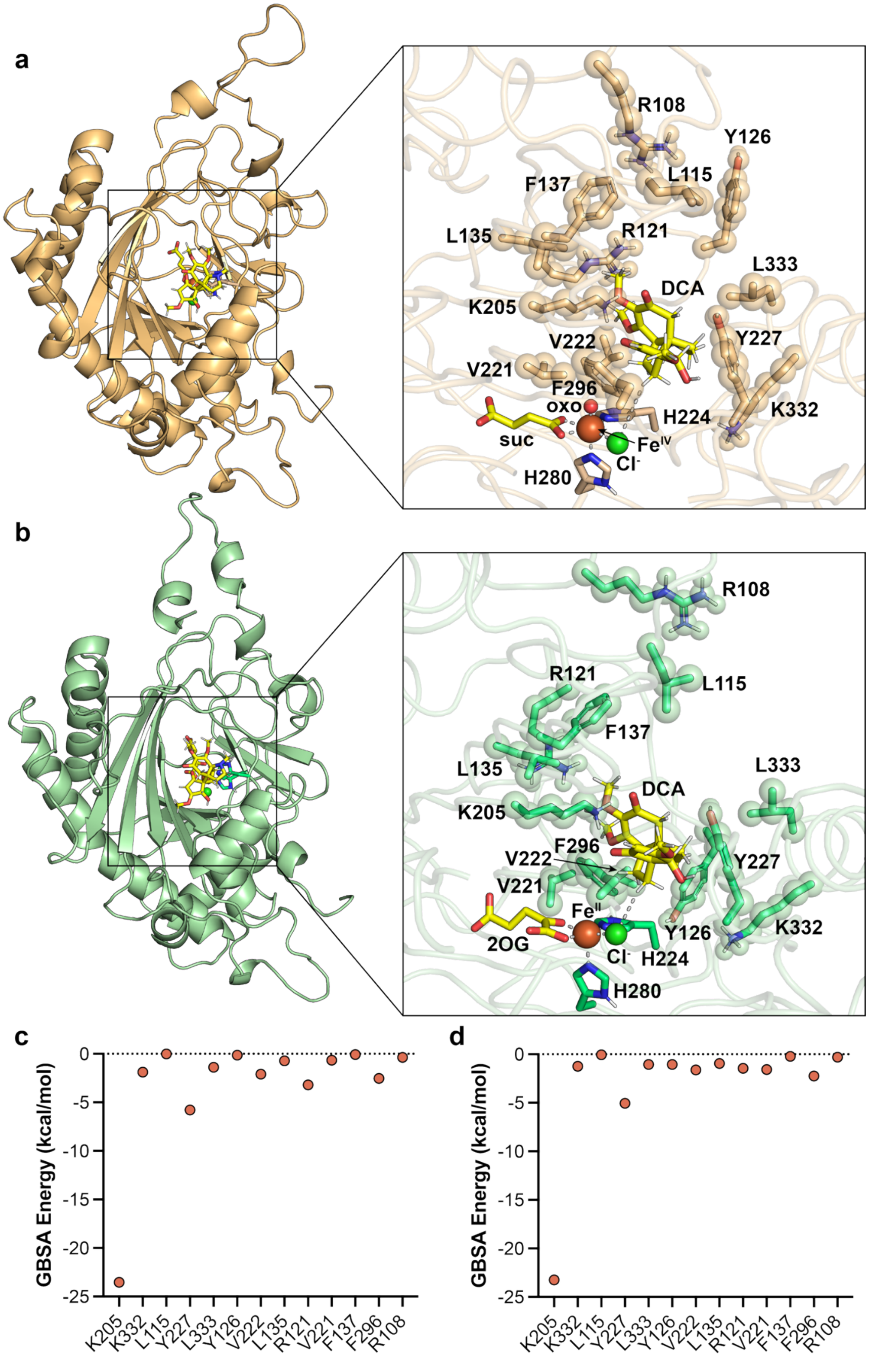

Fig. S31.

MM-GBSA hydrogen-bonding energy calculations from MD simulations of *Mc*DAH structural models. (**a**) *Mc*DAH structural model with the oxo in the axial position portraying relevant residues chosen in this study for alanine scanning. (**b**) 2OG-bound

*Mc*DAH structural model portraying relevant residues chosen in this study for alanine scanning. (In both panels **a** and **b**– Abbreviation; suc: succinate, DCA: dechloroacutumine) (**c**) MM-GBSA hydrogen-bonding energy calculation for *Mc*DAH structural model with the oxo in the axial position. (**d**) MM-GBSA hydrogen-bonding energy calculation for *Mc*DAH structural model with 2OG bound. In both panels **c** and **d**, the x-axis is ordered by low-to-high GBSA energy calculation based on the *Mc*DAH structural model with the oxo in the equatorial position.

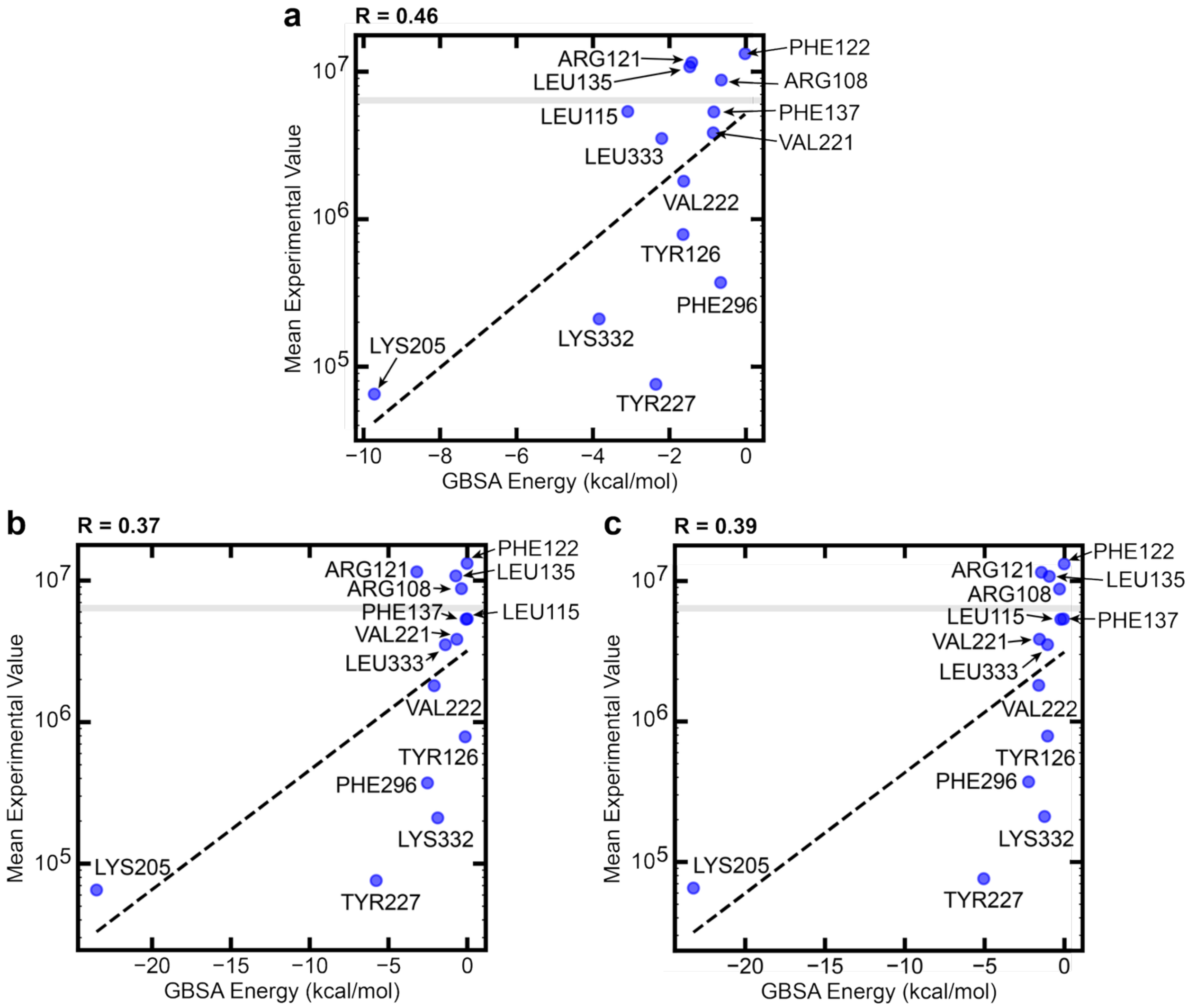

Fig. S32.

Parity plots comparing the MM-GBSA hydrogen-bonding energy calculations to *Mc*DAH alanine mutant assay data. (**a**) Comparison of the *Mc*DAH structural model with the oxo in the equatorial position with the corresponding alanine mutant experimental data. (**b**) Comparison of the *Mc*DAH structural model with the oxo in the axial position with the corresponding alanine mutant experimental data. (**c**) Comparison of the 2OG-bound *Mc*DAH structural model with the corresponding alanine mutant experimental data. For all panels, the y-axis is shown as $\log_{10}$-scale of LC-HRAM-MS peak areas of acutumine ($[M+H]^+$ = 398.13629 *m/z*). The R-value represents Pearson's correlation coefficient comparing the x-axis and y-axis values. Grey lines in each panel indicate the mean peak area of acutumine measured for WT *Mc*DAH.

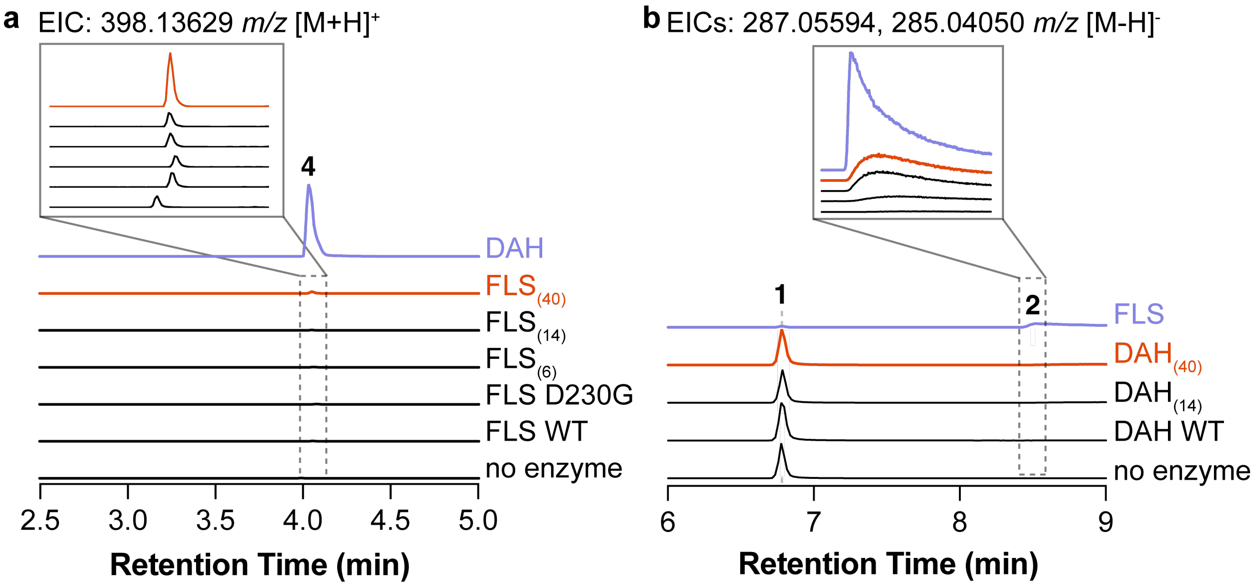

Fig. S33.

Qualitative LC-HRAM-MS chromatogram of *Mc*FLS mutants for minimal *Mc*DAH activity. (**a**) Extracted ion chromatograms (EICs) of acutumine, 398.13629 *m/z* ; $[M+H]^+$ of **4**. Retention time shift is observed in no enzyme sample due to changes in the chromatography buffer and as it was run independently from other samples present in this figure. Trace amounts of acutumine is detected in the control samples due to its presence from dechloroacutumine substrate used in the assays. All assays were performed with 5 µM enzyme under 20 minute reaction conditions using 2-oxoglutarate. (**b**) Combined extracted ion chromatograms (EICs) of dihydrokaempferol, 287.05594 *m/z*; $[M\text{-}H]^-$ of **1** and kaempferol, 285.04050 *m/z*; $[M\text{-}H]^-$ of **2**. $DAH_{(14)}$ enzyme has the β-sheet loop and substrate positioning loop regions swapped with their sequence alignment counterparts of *Mc*FLS. $DAH_{(44)}$ enzyme has the β-sheet loop, substrate positioning loop, and C-terminal helical loop regions swapped with their counterparts of *Mc*FLS sequence. All assays were performed with 5 µM enzyme under 20 minute reaction conditions using 2-oxoglutarate.

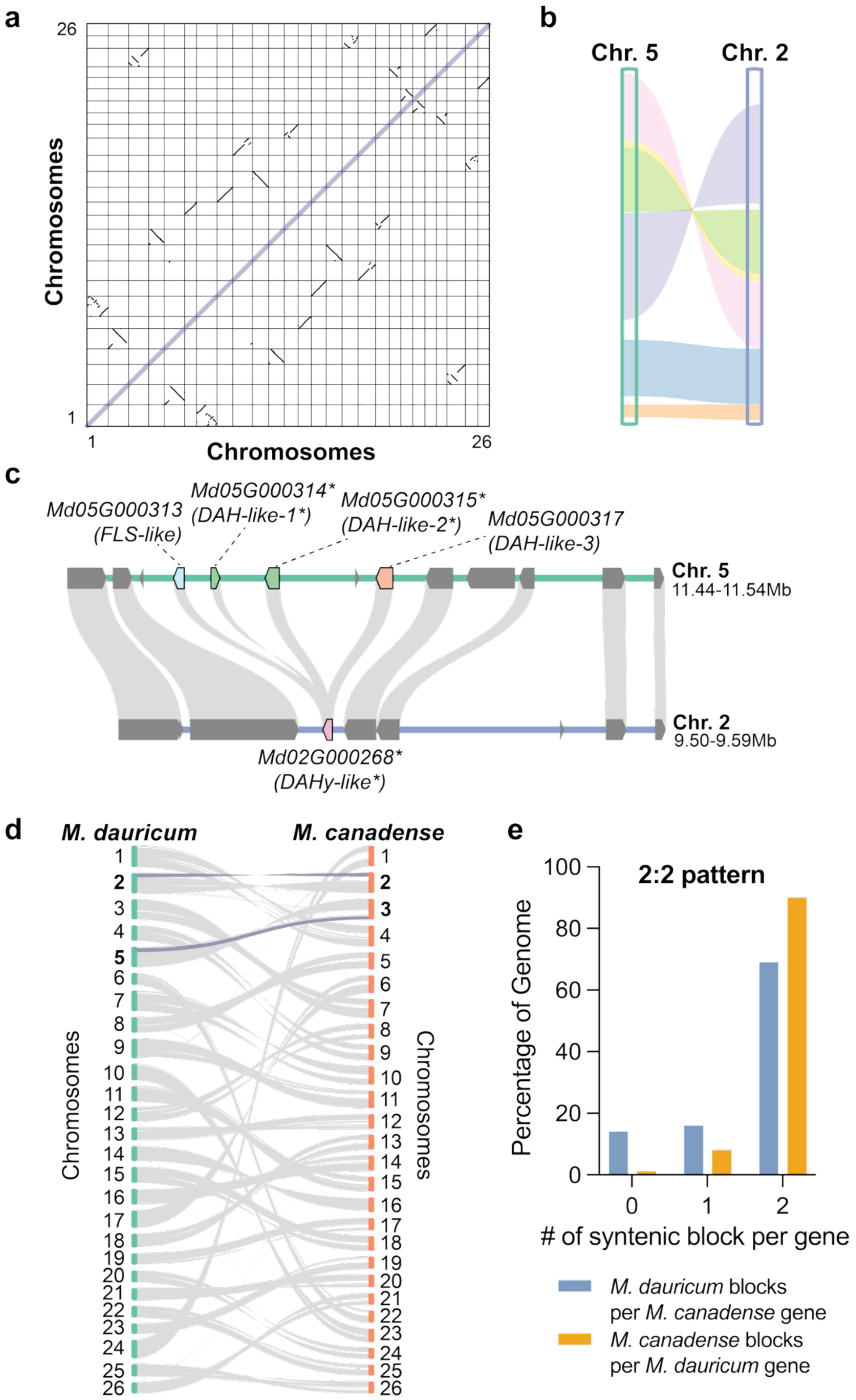

Fig. S34.

Comparative genomic analysis between *M. dauricum* and *M. canadense*. (**a**) Dot plot visualization of intragenomic syntenic regions within the *M. dauricum* genome

showcasing WGD. A total of 10,875 genomic pairs are shown with a C-score cutoff of 0.99. (**b**) Chromosomal-level synteny between *DAH-like*-containing chromosome 5 and *DAHy-like*-containing chromosome 2 of the *M. dauricum* genome. (**c**) Microsynteny of the WGD region on chromosome 5 and 2 containing *FLS-like*, *DAH-like*, and its paralogous genes. Asterisk denotes pseudogenes. (**d**) Syntenic comparison between *M. dauricum* and *M. canadense* genomes across their 26 chromosomes. Purple ribbons indicate syntenic regions that contain paralogous genes of *DAH*. (**e**) The syntenic depth between *M. dauricum* and *M. canadense*, showing 2:2 pattern.

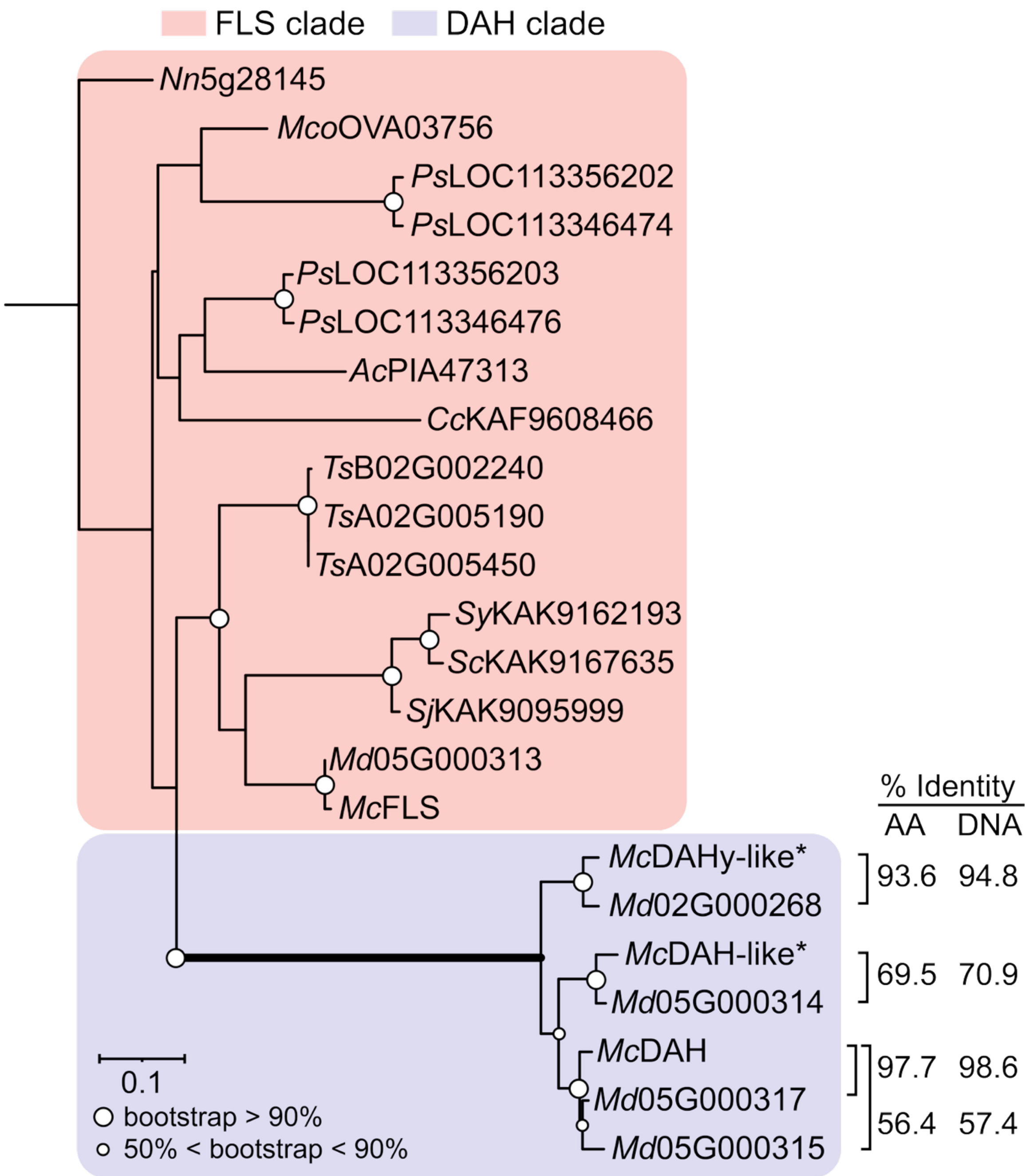

Fig. S35.

Maximum-likelihood phylogenetic tree illustrating the evolution of DAH in the order Ranunculales. The tree includes orthologs of DAH, DAH-like, DAHy-like and FLS from select Ranunculales species represented in Fig. 6a. Bootstrap statistics (200 replicates) are indicated at the tree nodes. The scale bar indicates evolutionary distance in substitutions per amino acid. Asterisks denote non-functional proteins. Sequence alignments of DAH ortholog pairs from *M. canadense* and *M. dauricum* were performed using MUSCLE (amino acid level) and CLUSTALW (codon-based DNA). Percent identity between aligned residues and bases is indicated.

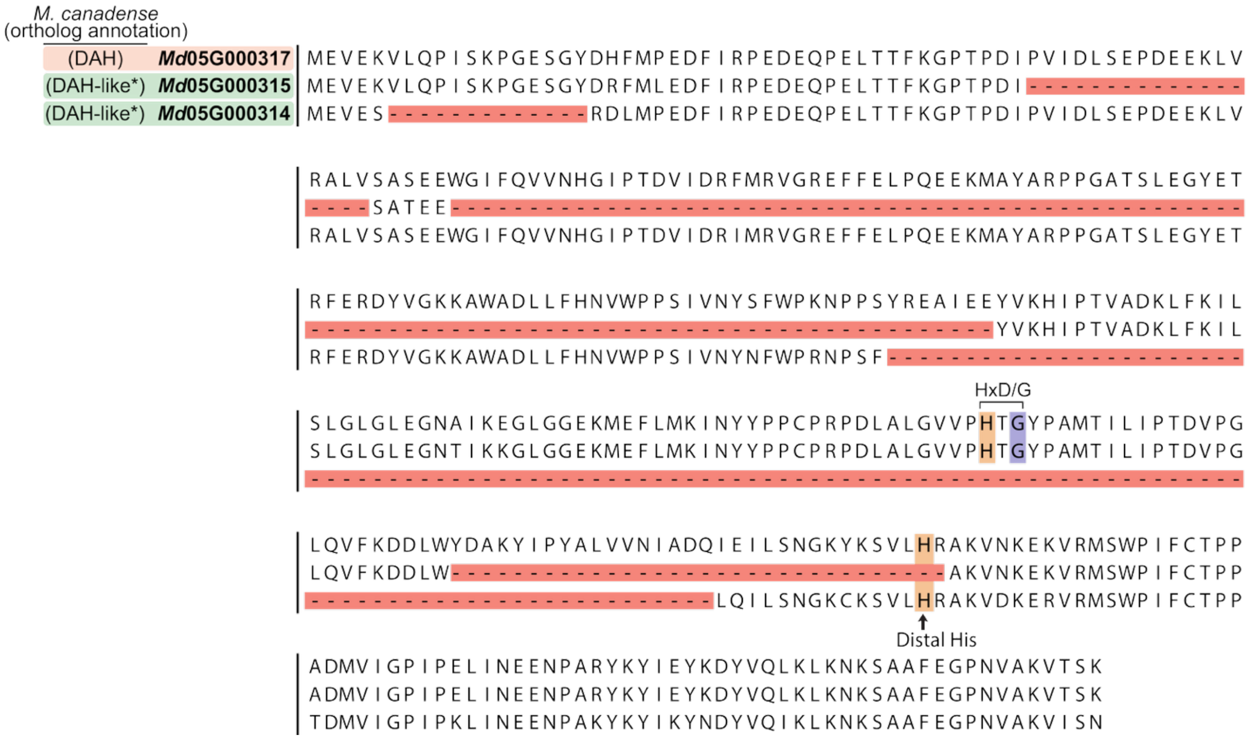

Fig. S36.

Multiple sequence alignment of DAH and DAH-like orthologs in *M. dauricum*. MUSCLE protein sequence alignment between *Md*05G000317 (DAH), *Md*05G000315 (DAH-like), and Md05G000314 (DAH-like) found in the *M. dauricum* genome. Relevant residues and motifs mentioned in this manuscript are highlighted.

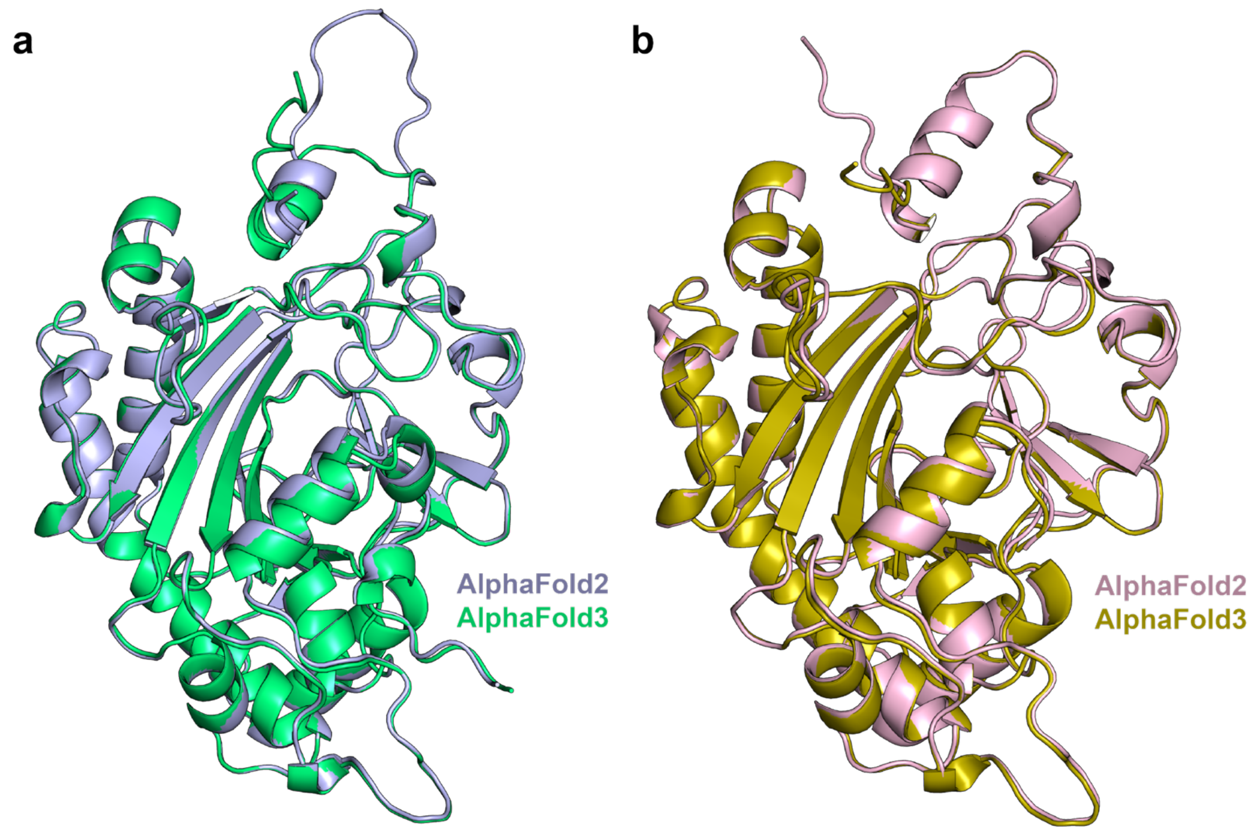

Fig. S37.
Structural comparison of AlphaFold2 and AlphaFold3 structural models. (**a**) DAH structural models from AlphaFold2 (light blue) and AlphaFold3 (green). Structural alignment resulted in an RMSD value of 0.295 for 2301 atoms.(**b**) FLS structural models from AlphaFold2 (pink) and AlphaFold3 (dark yellow). Structural alignment resulted in an RMSD value of 0.318 for 2266 atoms.

Table S1.
Summary of sequencing reads used for assembling and polishing the *Menispermum canadense* genome.

	Data Type	Raw Reads	Raw Bases (G)	Clean Reads	Clean Bases (G)	Q20 (%)	Q30 (%)	GC content (%)
**Long Read (Primary Assembly)**	PacBio Sequel II (HIFI CCS)	3.079435 M	48.571952	3.079435 M	48.571952	98.06	95.41	35.42
**Short Read (QC)**	Illumina NovaSeq (Paired End 150bp : 150bp)	1.801201 G	270.180090	1.785581 G	266.380866	97.64	92.95	35.12
**Hi-C (Scaffolding)**	Illumina NovaSeq (Paired End 150bp : 150bp)	1.084218 G	162.632754	1.034976 G	154.857483	95.81	90.30	35.86

Table S2.
Length and scaffolds for chromosome-level assembly of the *Menispermum canadense* genome.

Pseudo-chromosome	# Contigs	Size (bp)
Chr1	1	45,646,067
Chr2	1	44,085,362
Chr3	1	42,833,333
Chr4	1	42,297,172
Chr5	1	42,242,317
Chr6	1	40,690,050
Chr7	1	37,974,986
Chr8	1	36,980,421
Chr9	1	36,537,451
Chr10	1	36,006,012
Chr11	1	35,609,015
Chr12	1	34,818,747
Chr13	1	34,191,516
Chr14	1	33,879,814
Chr15	1	33,347,802
Chr16	1	32,511,916
Chr17	1	31,451,445
Chr18	1	31,054,424
Chr19	1	30,121,998
Chr20	1	29,630,568
Chr21	1	29,598,887
Chr22	1	29,497,318
Chr23	1	27,578,300
Chr24	1	24,848,486
Chr25	1	24,327,722
Chr26	1	22,787,794
unmapped	1249	79,359,561

Table S3.
Sequence reads mapped onto the *Menispermum canadense* genome using bwa-mem (v0.7.17-r1188)(*110*) (Illumina) and minimap (v2.24-r1122)(*111*) (Transcripts).

Property	Number
Primary aligned reads (Illumina)	1,800,058,803
Total number of reads (Illumina)	1,820,675,006
Primary aligned reads (Transcripts)	733,893
Total number of reads (Transcripts)	734,023

Table S4.
***Menispermum canadense* genome annotation statistics.**

Property	Number
Total number of protein-coding genes	65,843
Total number of exons	284,345
Average exon length (bp)	173.15
Average protein length (aa)	290.9
Average gene length (bp)	3088.44

Table S5.
Statistics from genome quality assessment by Merqury(*18*).

Property	Number
k-mer used	20
Solid *k*-mers in assembly	403,045,961
Total solid *k*-mers in the read set	479,987,586
Unique *k*-mers in assembly	20,962
k-mers in both assembly and read set	969,874,501
Assembly consensus quality value (QV)	59.6631
Error rate	1.08067×10^{-6}

Table S6.
Source of plant genomes for phylogenomic and comparative genomics analyses.

Plant Taxon	Short/Common name	Reference
Aquilegia coerulea	Rocky Mountain Columbine	NCBI (GCA002738505)
Arabidopsis thaliana	*Arabidopsis*	Araport11
Amborella trichopoda	*Amborella*	NCBI (GCA000471905)
Coptis chinensis	Chinese Goldthread	NCBI (GCA015680905)
Cinnamomum kanehirae	Stout Camphor Tree	NCBI (GCA003546025)
Menispermum canadense	Common Moonseed	This manuscript
Macleaya cordata	Plume Poppy	NCBI (GCA002174775)
Menispermum dauricum	Asian Moonseed	https://doi.org/10.6084/m9.figshare.23509239 (*112*)
Nelumbo nucifera	Sacred Lotus	nelumbo.cngb.org
Oryza sativa	Rice	rice.uga.edu
Papaver somniferum	Opium Poppy	NCBI (GCA003573695 for phylogenetics and GCF003573695 for synteny)
Stephania cephalantha	Money Turtle Vine	NCBI (GCA039657365)
Stephania japonica	Snake Vine	NCBI (GCA039657345)
Stephania yunnanensis	Chinese Stephania (Shanwugui)	NCBI (GCA039657365)
Theobroma cacao	Cacao	cocoa-genome-hub.southgreen.fr
Tinospora sagittata	Radix Tinosporae (Jin Guo Lan)	NCBI (GCA035771175)
Vitis vinifera	Grape	genoscope.cns.fr/externe/GenomeBrowser/Vitis/

Table S7.
Prediction of orthologous groups by OrthoFinder (*19*).

Plant Taxon	Total curated genes	Number of genes in orthogroups	Number of unassigned genes	Number of orthogroups containing species	Percentage of orthogroups containing species	Number of species-specific orthogroups	Number of genes in species-specific orthogroups
Aquilegia coerulea	24102	218897	2205	12719	44.7%	303	977
Arabidopsis thaliana	*28258*	26225	2033	12764	44.9%	976	4340
Amborella trichopoda	25477	21296	4181	12968	45.6%	795	3852
Coptis chinensis	34241	32667	1574	13947	49.0%	1148	5206
Cinnamomum kanehirae	24115	22763	1352	12292	43.2%	326	1472
Menispermum canadense	55164	47394	7770	15361	54.0%	1752	15244
Macleaya cordata	20525	19724	801	12504	43.9%	105	332
Nelumbo nucifera	28619	24084	4535	13065	45.9%	648	1991
Oryza sativa	51870	43687	8183	14141	49.7%	2525	18052
Papaver somniferum	37269	32491	4778	14071	49.5%	1786	6893
Stephania japonica	25157	22357	2800	12984	45.6%	599	3600
Theobroma cacao	21590	21132	458	12620	44.4%	247	1022
Vitis vinifera	23743	20878	2865	12575	44.2%	380	1140

Plant Taxon	Total curated genes	Number of genes in orthogroups	Number of unassigned genes	Number of orthogroups containing species	Percentage of orthogroups containing species	Number of species-specific orthogroups	Number of genes in species-specific orthogroups
Aquilegia coerulea	24102	21852	2250	13368	45.4%	365	1181
Coptis chinensis	34241	32659	1582	14608	49.6%	1214	5911
Menispermum canadense	55164	48005	7159	17944	60.9%	1244	8533
Macleaya cordata	20525	19710	815	13076	44.4%	120	470
Menispermum dauricum	40951	37064	3887	16948	57.6%	628	1593
Nelumbo nucifera	28619	23959	4660	13552	46.0%	764	2410
Papaver somniferum	37269	32474	4795	14697	49.9%	1923	7624
Stephania cephalantha	29012	25865	3147	15329	52.1%	434	1655
Stephania japonica	25157	22738	2419	14160	48.1%	404	2316
Stephania yunnanensis	29379	26349	3030	15400	52.3%	469	2169
Tinospora sagittata	22386	21743	643	13993	47.5%	190	560

Table S8.
Strains and plasmids used in this study.

Strain	Description	Source
BL21 (DE3)	*fhuA2 [lon] ompT gal (λ DE3) [dcm] ΔhsdS*	New England BioLabs

Plasmid	Description	Source
pHis8-4b-His_8-*Mc*DAH	His_8-*Mc*DAH (T7), *lacI*, *lacO*, Kan^R, F1-ori	This study
pHis8-4b-His_8-*Mc*FLS	His_8-*Mc*FLS (T7), *lacI*, *lacO*, Kan^R, F1-ori	This study
pHis8-4b-His_8-*Mc*FLS D230G	His_8-*Mc*FLS-D230G (T7), *lacI*, *lacO*, Kan^R, F1-ori	This study
pHis8-4b-His_8-*Mc*$FLS_{(6)}$	His_8-*Mc*$FLS_{(6)}$ (T7), *lacI*, *lacO*, Kan^R, F1-ori	This study
pHis8-4b-His_8-*Mc*$FLS_{(14)}$	His_8-*Mc*$FLS_{(14)}$ (T7), *lacI*, *lacO*, Kan^R, F1-ori	This study
pHis8-4b-His_8-*Mc*$FLS_{(44)}$	His_8-*Mc*$FLS_{(44)}$ (T7), *lacI*, *lacO*, Kan^R, F1-ori	This study
pHis8-4b-His_8-*Mc*$DAH_{(14)}$	His_8-*Mc*$DAH_{(14)}$ (T7), *lacI*, *lacO*, Kan^R, F1-ori	This study
pHis8-4b-His_8-*Mc*$DAH_{(44)}$	His_8-*Mc*$DAH_{(44)}$ (T7), *lacI*, *lacO*, Kan^R, F1-ori	This study
pHis8-4b-His_8-*At*F3H D219G	His_8-*At*F3H D129G (T7), *lacI*, *lacO*, Kan^R, F1-ori	This study
pBA0221-0141-*Hn*H6H D219A-His_8	*Hn*H6H D219A-His_8 (T7), *lacI*, *lacO*, Kan^R, F1-ori, pBR322-ori, Tet^R	This study
pHis8-4b-His_8-*Mc*DAH T231A	His_8-*Mc*DAH T231A (T7), *lacI*, *lacO*, Kan^R, F1-ori	This study
pHis8-4b-His_8-*Mc*DAH N262A	His_8-*Mc*DAH N262A (T7), *lacI*, *lacO*, Kan^R, F1-ori	This study
pHis8-4b-His_8-*Mc*DAH K205A	His_8-*Mc*DAH K205A (T7), *lacI*, *lacO*, Kan^R, F1-ori	This study
pHis8-4b-His_8-*Mc*DAH K332A	His_8-*Mc*DAH K332A (T7), *lacI*, *lacO*, Kan^R, F1-ori	This study
pHis8-4b-His_8-*Mc*DAH L115A	His_8-*Mc*DAH L115A (T7), *lacI*, *lacO*, Kan^R, F1-ori	This study
pHis8-4b-His_8-*Mc*DAH Y227A	His_8-*Mc*DAH Y227A (T7), *lacI*, *lacO*, Kan^R, F1-ori	This study
pHis8-4b-His_8-*Mc*DAH L333A	His_8-*Mc*DAH L333A (T7), *lacI*, *lacO*, Kan^R, F1-ori	This study
pHis8-4b-His_8-*Mc*DAH Y126A	His_8-*Mc*DAH Y126A (T7), *lacI*, *lacO*, Kan^R, F1-ori	This study
pHis8-4b-His_8-*Mc*DAH V222A	His_8-*Mc*DAH V222A (T7), *lacI*, *lacO*, Kan^R, F1-ori	This study
pHis8-4b-His_8-*Mc*DAH L135A	His_8-*Mc*DAH L135A (T7), *lacI*, *lacO*, Kan^R, F1-ori	This study
pHis8-4b-His_8-*Mc*DAH R121A	His_8-*Mc*DAH R121A (T7), *lacI*, *lacO*, Kan^R, F1-ori	This study
pHis8-4b-His_8-*Mc*DAH V221A	His_8-*Mc*DAH V221A (T7), *lacI*, *lacO*, Kan^R, F1-ori	This study
pHis8-4b-His_8-*Mc*DAH F137A	His_8-*Mc*DAH F137A (T7), *lacI*, *lacO*, Kan^R, F1-ori	This study
pHis8-4b-His_8-*Mc*DAH F296A	His_8-*Mc*DAH F296A (T7), *lacI*, *lacO*, Kan^R, F1-ori	This study
pHis8-4b-His_8-*Mc*DAH R108A	His_8-*Mc*DAH R108A (T7), *lacI*, *lacO*, Kan^R, F1-ori	This study
pHis8-4b-His_8-*Mc*DAH R108A/R121A	His_8-*Mc*DAH R108A/R121A (T7), *lacI*, *lacO*, Kan^R, F1-ori	This study

Table S9.
Oligonucleotide sequences reported in this study.

Name	Sequence (5' - 3')
His$_8$-*Mc*DAH-T231A-F (QuikChange)	tgggtatgagaatggccatggcagggtagcc
His$_8$-*Mc*DAH-T231A-R (QuikChange)	ggctaccctgccatggccattctcataccca
His$_8$-*Mc*DAH-N262A-F (QuikChange)	aatctcgatttgatcagctatggcaaccacgagagcatatggaatg
His$_8$-*Mc*DAH-N262A-R (QuikChange)	cattccatatgctctcgtggttgccatagctgatcaaatcgagatt
His$_8$-*Mc*DAH-K205A-F (QuikChange)	catggtgggtagtagttgattgccatcagaaactccatcttctc
His$_8$-*Mc*DAH-K205A-R (QuikChange)	gagaagatggagtttctgatggcaatcaactactacccaccatg
His$_8$-*Mc*DAH-K332A-F (QuikChange)	gcagcactcttgttcttgagcgcgagttgcacataatctttgta
His$_8$-*Mc*DAH-K332A-R (QuikChange)	tacaaagattatgtgcaactcgcgctcaagaacaagagtgctgc
His$_8$-*Mc*DAH-L115A-F (QuikChange)	catagccctccgcagaggttgcaccaggaggcc
His$_8$-*Mc*DAH-L115A-R (QuikChange)	ggcctcctggtgcaacctctgcggagggctatg
His$_8$-*Mc*DAH-Y227A-F (QuikChange)	aatggtcatggcaggggcgccagtgtgtggaacc
His$_8$-*Mc*DAH-Y227A-R (QuikChange)	ggttccacacactggcgcccctgccatgaccatt
His$_8$-*Mc*DAH-L333A-F (QuikChange)	aaaagcagcactcttgttcttggccttgagttgcacataatctttg
His$_8$-*Mc*DAH-L333A-R (QuikChange)	caaagattatgtgcaactcaaggccaagaacaagagtgctgctttt
His$_8$-*Mc*DAH-Y126A-F (QuikChange)	ccaagccttcttgcccacagcgtctctctcaaaccttgtt
His$_8$-*Mc*DAH-Y126A-R (QuikChange)	aacaaggtttgagagagacgctgtgggcaagaaggcttgg
His$_8$-*Mc*DAH-V222A-F (QuikChange)	agccagtgtgtggagccacaccaagagcc
His$_8$-*Mc*DAH-V222A-R (QuikChange)	ggctcttggtgtggctccacacactggct
His$_8$-*Mc*DAH-L135A-F (QuikChange)	ccagacgttgtggaacaaagcatcagcccaagccttcttg
His$_8$-*Mc*DAH-L135A-R (QuikChange)	caagaaggcttgggctgatgctttgttccacaacgtctgg
His$_8$-*Mc*DAH-R121A-F (QuikChange)	ccacatagtctctctcaaacgctgtttcatagccctccaaag
His$_8$-*Mc*DAH-R121A-R (QuikChange)	ctttggagggctatgaaacagcgtttgagagagactatgtgg
His$_8$-*Mc*DAH-V221A-F (QuikChange)	gccagtgtgtggaaccgcaccaagagccaaatc
His$_8$-*Mc*DAH-V221A-R (QuikChange)	gatttggctcttggtgcggttccacacactggc
His$_8$-*Mc*DAH-F137A-F (QuikChange)	gaggccagacgttgtgggccaaaagatcagcccaag
His$_8$-*Mc*DAH-F137A-R (QuikChange)	cttgggctgatcttttggcccacaacgtctggcctc
His$_8$-*Mc*DAH-F296A-F (QuikChange)	tgctggtggtgtacaggctatcggccacgacatcc
His$_8$-*Mc*DAH-F296A-R (QuikChange)	ggatgtcgtggccgatagcctgtacaccaccagca
His$_8$-*Mc*DAH-R108A-F (QuikChange)	caccaggaggcgcagcataggccatcttctcctc
His$_8$-*Mc*DAH-R108A-R (QuikChange)	gaggagaagatggcctatgctgcgcctcctggtg

Table S10.
Gene sequences reported in this study.

Name	Sequence (5’ - 3’)
McDAH **(FUN_009915)**	ATGGAAGTAGAGAAGGTCCTCCAACCAATCTCCAAAAGCGGTGAAAGCGGATATGACCACTTCATGCC<br>GGAAGATTTCATTCGGCCGGAAGATGAACAGCCGGAATTAACCACATTCAAAGGTCCGACACCGGAC<br>ATTCCGGTCATTGACTTGTCGGAGCCGAATGAGGAGAAGCTGGTAAGAGCATTAGTCAGCGCTTCAG<br>AAGAATGGGGCATATTCCAGGTGGTAAATCATGGCATTCCGACGGACGTGATTGATAAGTTTATGCGC<br>GTTGGCCGCGAGTTCTTCGAGCTCCCGCAAGAGGAGAAGATGGCTTACGCTCGCCCGCCGGGCGCC<br>ACAAGTTTAGAAGGGTATGAAACCCGTTTTGAGCGCGACTATGTCGGCAAGAAAGCGTGGGCTGACC<br>TGCTCTTCCACAATGTATGGCCTCCCTCCATCGTGAACTACAGTTTCTGGCCGAAGAACCCGCCTTCC<br>TATCGGGAAGCCACTGAGGAGTACGCGAAACACATCCCGATCGTCGCGGACAAATTATTCAAAATACT<br>GTCACTGGGCCTTGGCCTGGAAGGCAACTCGATCAAGGAAGGGCTCGGTGGTGAGAAAATGGAGTTT<br>CTCATGAAAATAAATTACTATCCACCGTGTCCCCGGCCGGACCTCGCTCTCGGCGTAGTACCGCATAC<br>CGGCTATCCGGCTATGACGATTTTGATACCCACGGACGTTCCTGGTTTACAAGTATTCAAAGACGATTT<br>ATGGTACGACGCCAAATACATTCCATACGCGCTGGTCGTAAATATCGCCGACCAGATTGAGATACTGT<br>CTAATGGAAAATACAAGTCCGTGCTCCACCGCGCAAAAGTAAACAAGGAGAAAGTGCGTATGAGCTG<br>GCCAATATTCTGTACCCCGCCAGCTGACATGGTCATTGGTCCGATACCTGAGCTGATAAATGAGGAGA<br>ACCCAAGCCGTTACAAATACATCGAGTATAAAGACTATGTACAGCTGAAACTGAAGAATAAATCCGCC<br>GCCTTTGAGGGACCCAACGTGGCGAAGGTCACGTCGAAGTGA
McDAHy-like **(FUN_003698)**	ATGGAAGTAGAGTACTCCAAACCGGAAGACAGCGGAGATGACGACTTCATGCCGGAAGATTTCATTC<br>GGGCTGAAGATGAACAGCCGGAAGTGACCACATTCAAAGGTCCGACACCGGACATTCCGATGATTGA<br>CTTGTCGGAGCCGGACGAGGAGAAGCTGGTAAGAGCAGTTGTCAGCGGGTCAGAAGAATGGGGCAT<br>ATTCCAGGTGGTAAATCATGGCATTCCGACGGACGTGATTGATCGTTTTCAGAAAGTTGGCCGCGAGT<br>TCTTCGAGCTCCCGCAAGAGGAGAAGATGGCTTACGCTCGCCCGCCGGGCGCCACAAGTTTAGAAG<br>GGTATGAAACCCGTTTTGAGCGCGACTATGTCGGCAAGAAAGCGTGGGCTGACCTGCTCTTCCACAA<br>TGTATGGCCTCCCTCCATCGTGAACTACCACTTCTGGCCGAACAACCCGACGGGCCTGTCTGCAAAG<br>AATGGTTGGGAAGCCACTGAGGAGTACGTAAAATTTATCCCGATCGTCGCGGACAAATTATTCAAAAT<br>ACTGTCACTGGGCCAAGGCCTGAAGGGCAACACCATCAAGGAAGTGTTTGGTGGTGAGAAAATGGAG<br>TACCTCATGAAAATAAATTACTATCCACCGTGTCCCCGGCCGGACCTCGCTCTCGGCTTGGTACCGCA<br>TACCGGCTATCCGGCTATGATTATTTTGATACCCACGGACGTTCCTAGCTTACAAGTATTCAAAGACGA<br>TTTATGGTACGACGTGAAATACATTCCATACGCGCTGGTCGTAAATATCGCCGACCAGATTGAGATAC<br>TGTCTAATGGAAAATACAAGTCCGTGCTCCACCGCGTCAAAGTAAACAAGGAGAAATTACGTATGAGC<br>TGGCCAATATTCTGTACCCCGCCAGCTGACATGGTCATTGGTCCGATATCCGAGCTGATAAATGAGGA<br>GAACCCAGCCAAGTACAAATACATCAAGTATAAAGACTATGTACAGATCAAACTGAAGAATCGGTCCG<br>CCGCCTTTGAGGGACCCAACGTGGCGAAGGTCACGTCGAACTGA
McDAH-like **(FUN_009914)**	ATGGAGGTAGAGTCACGAGACTTGATGCCCGAAGACTTCATTAGGCCTGAAGACGAGCAGCCTGAGC<br>TCACCACTTTCAAGGGTCCAACCCCAGACATTCCGGTGATCGATCTGAGTGAGCCGGACGAGGAGAA<br>GCTGGTGAGAGCTCTTGTGAGTGCCAGTGAAGAGTGGGGGATCTTCCAAGTGGTGAATCACGGGATT<br>CCGGCAGATGTGATCGACAGGTTTATGAGGGTTGGGAGAGAGTTTTTCGAGTTACCACAAGAGGAGA<br>AGATGGCCTACGCTAGGCCTCCTGGTGCAACCTCTTTGGAGGGCTATGAAACAAGGTTTGAGAGAGA<br>CTATGTGGGCAAGAAGGCTTGGGCTGATCTCTTGTTCCACAACGTTTGGCCTCCCTCCATTGTTAATT<br>ACAACTTCTGGCCCAAGAACCCTCCTTCTTACAGGGAGGCTACAGAAGAGTATGTGAAGTACATACCA<br>ATAGTGGCAGATAAGTTGTTCAAGTGTCTCTCTCTAGGGCTTGGGCTTGAAGGGAATGCTATGAAAGA<br>AGGACTTGGAGGAGAAAAAATGGAGTTTCTGCTGAAGATCAACTACTACCCACCGTGCCCACGTCCTG<br>ATTTGGCTCTTGGTGTGGTTCCACACACTGGCTACCCTGCCATGACCATTCTCATACCCACCGATCGA<br>TGTGCCTGCAATGGCAAGTATAAGAGTGTCTTGCATCGAGCAAAGGTAGACAAGGAGAGGGTGAGGA<br>TGTCGTGGCCAATATTCTGCACGCCACCGACAGATATGGTTATTGGACCGATTCCAAAGCTGATCAAC<br>GAAGAAAATCCAGCGAAGTATAAATATATTAAGTACAACGATTATGTGCAAATTAAGCTCAAAAACAAG<br>AGTGCTGCTTTTGAGGGTCCTAATGTAGCCAAGCCATATGAAGATTAG
McFLS **(FUN_009913)**	ATGGAGGTCGATTATCAGAGAGTACAAGTCATAGCTACTCTATCGAAGAACACCACCGACGCCGATAA<br>CATTCCCGAGGAGTTCATCAGGCCAGAGCGAGAACAGCCGGCGCTCACAACGTCCGCCGGCCCTGT<br>TCCTGAGATCCCCACCGTCGATCTCAGCGAGTCGTCATCAGATGAGGAGAGTCTCGTGCGTTCGATA<br>CGTGATGCGAGCCGAGAGTGGGGGTTTTTCCAGATCGTGAACCACGGGATCCCAATACAGGTGATCG<br>AGAGGTTGCAGAGAGCGGGGAAGGAGTTCTTCGAGCTATCGCAGGAGGAGAAAGAAGCGTACGCAA<br>GGGTTTCTGGAGATAGTGGTTTGGAAGGGTATGGGACGAAGCTTCAGAAGGAGATTGAAGGGAAGAA<br>GGCTTGGGTTGATTACTTGTTTCACAACGTCTGGCCTCCGTCTCGGATCAACTACAGGTTCTGGCCCA<br>AGAACCCAAGTTATTACAGGGAAGCTAATGAAGAGTACGCGAAGTGGTTGAAGGGGGTGGTGGAGAA<br>GCTGATGGAGTGGCTGTCTTTAGGGTTGGGGCTTGAAAAGAATGCACTGATAGATGGAGTTGGCGGA<br>GAATCAATGGAGTATCTTCTCAAGATAAACTACTACCCGCCATGTCCTCGACCTGATCTTGCTCTCGGA<br>GTAGTACCGCATACCGACATGTCTGCTATCACTGTGCTCGTGCCCAACGACGTACCGGGCCTTCAAG<br>TCTTCAAAGACGAGCACTGGTTCGACGCCAAGTACATCCCTAACGCCCTCATTGTCCACATCGGTGAC<br>CAGGTCGAGATAATGAGTAACGGGAAGTATAAGAGTGTGCTTCATAGGACTACTGTGAATAAAGAGAA<br>GGCAAGGATGTCATGGCCAGTGTTTTGCTCACCACCAGCTGAGCATGTTGTTGGGCCACTCCCGCAA<br>CTGGTTAATGAAGAGAACCCACCAAAGTATAAGACCAAGAAATACAAGGATTATGAGTACTGCAAGCT<br>CAACAAACTGCCACAGTAG

Name	Sequence (5' - 3')
McFLS$_{(6)}$	ATGGAGGTCGATTATCAGCGCGTACAGGTTATAGCGACGCTCAGTAAAAATACAACCGATGCGGATAA<br>TATTCCCGAAGAATTTATACGGCCCGAGCGCGAGCAACCTGCCCTGACAACAAGTGCAGGACCAGTT<br>CCAGAAATCCCGACCGTGGACCTGTCAGAGTCTTCAAGCGATGAAGAGAGTTTGGTTCGCTCAATTCG<br>GGATGCGTCACGAGAATGGGGTTTTTTTCAAATTGTAAACCACGGCATTCCTATCCAGGTAATTGAGC<br>GTTTACAGCGTGCCGGTAAAGAATTCTTCGAATTGTCCCAGGAAGAAAAAGAAGCGTATGCACGCGTT<br>TCTGGGGATAGTGGCTTAGAAGGTTATGAAACGCGCTTCGAACGTGATTACGTGGGAAAAAAAGCATG<br>GGTAGACTACCTTTTTCATAATGTATGGCCTCCTTCCCGCATTAATTACCGCTTCTGGCCTAAAAACCC<br>CTCGTACTATCGTGAGGCCAACGAAGAGTACGCCAAATGGCTGAAAGGGGTTGTCGAAAAATTAATG<br>GAGTGGTTATCCCTCGGTCTGGGCCTTGAAAAAAATGCCCTGATAGATGGCGTGGGTGGAGAATCCA<br>TGGAGTATCTCTTAAAAATAAATTACTATCCGCCTTGCCCACGTCCAGACCTGGCACTGGGTGTGGTG<br>CCTCATACCGACATGTCTGCTATCACCGTACTGGTGCCGAATGACGTTCCTGGTTTACAAGTTTTTAAA<br>GATGAACATTGGTTTGATGCTAAATATATTCCGAATGCACTGATTGTTCATATTGGTGATCAGGTCGAG<br>ATAATGTCCAACGGGAAATATAAAAGTGTTCTGCACCGCACTACAGTGAACAAAGAAAAAGCACGCAT<br>GAGCTGGCCGGTGTTTTGCTCCCCTCCAGCGGAACATGTGGTAGGGCCTTTGCCGCAGTTAGTCAAC<br>GAGGAAAACCCGCCGAAGTATAAAACCAAAAAATACAAAGATTATGAATATTGCAAACTTAATAAACTG<br>CCCCAGTAA
McFLS$_{(14)}$	ATGGAGGTAGATTATCAGCGCGTACAAGTGATAGCTACTCTTTCTAAAAATACAACGGATGCGGATAA<br>CATTCCGGAAGAATTTATTCGCCCTGAGCGTGAGCAGCCGGCCCTGACCACAAGTGCCGGACCGGTG<br>CCGGAGATTCCCACAGTGGATTTGAGTGAATCCAGTAGTGATGAAGAAAGTCTGGTCCGTTCAATTCG<br>CGATGCATCTAGAGAATGGGGCTTCTTTCAGATTGTAAATCATGGGATTCCGATTCAGGTAATAGAAC<br>GGCTGCAGCGGGCCGGGAAAGAATTTTTTGAACTGTCTCAGGAAGAGAAAGAGGCTTACGCACGTGT<br>GTCAGGCGACTCAGGGCTCGAAGGTTATGAAACACGTTTTGAGCGTGACTATGTCGGTAAGAAAGCA<br>TGGGTTGACTATTTGTTTCACAATGTCTGGCCGCCCTCAAGAATAAACTACCGTTTTTGGCCTAAAAAT<br>CCCTCATACTATCGGGAAGCGAATGAGGAATATGCTAAATGGCTGAAAGGTGTGGTAGAGAAACTTAT<br>GGAATGGCTCTCTTTAGGACTGGGCCTGGAAAAAAATGCGCTGATCGATGGCGTAGGCGGAGAGTCC<br>ATGGAATATCTTTTAAAAATCAATTACTACCCGCCCTGTCCGAGACCGGATCTGGCGCTTGGCGTGGT<br>TCCTCACACAGGCTATCCAGCCATGACGATATTGATTCCTAATGATGTACCTGGGCTTCAGGTGTTTAA<br>AGATGAGCATTGGTTTGACGCTAAATACATTCCGAATGCATTGATAGTTCATATAGGCGATCAGGTGG<br>AAATTATGTCGAACGGCAAATATAAGTCTGTGTTACATAGAACTACAGTTAACAAGGAAAAAGCCCGCA<br>TGTCGTGGCCTGTGTTTTGTAGTCCGCCAGCAGAACATGTGGTGGGCCCGCTGCCTCAGCTTGTTAA<br>CGAAGAAAACCCTCCAAAATATAAGACAAAGAAATACAAGGACTACGAATACTGTAAATTAAATAAGTT<br>ACCGCAGTAG
McFLS$_{(44)}$	ATGGAAGTAGACTATCAGCGGGTTCAAGTAATCGCTACCCTTAGTAAGAATACCACAGACGCGGACAA<br>CATACCGGAAGAGTTCATTCGGCCAGAGCGCGAGCAACCGGCCCTGACGACAAGCGCAGGTCCAGT<br>CCCAGAGATCCCAACAGTTGACCTTTCTGAGAGCTCATCAGACGAGGAGTCGTTGGTACGTTCGATTC<br>GAGACGCTTCGCGTGAATGGGGATTCTTCCAAATTGTGAACCACGGTATACCGATCCAAGTCATTGAG<br>CGCCTGCAACGGGCCGGCAAAGAGTTCTTCGAGCTTAGTCAAGAGGAAAAGGAAGCATACGCACGC<br>GTCTCGGGAGATTCTGGGTTGGAAGGGTATGAGACTCGTTTCGAGCGTGACTACGTTGGTAAGAAGG<br>CGTGGGTAGACTATCTGTTTCACAACGTCTGGCCGCCATCTCGCATCAACTACCGCTTCTGGCCAAAG<br>AACCCGTCTTACTACCGTGAGGCTAATGAGGAATACGCTAAGTGGCTGAAAGGTGTAGTGGAGAAGC<br>TGATGGAATGGTTGAGCCTTGGTTTAGGACTGGAAAAGAACGCACTCATAGACGGTGTTGGTGGGGA<br>GAGCATGGAGTATCTTCTCAAAATCAACTACTATCCGCCTTGCCCACGGCCGGACCTTGCCTTAGGGG<br>TAGTTCCCCACACAGGCTACCCAGCAATGACCATCCTGATCCCGAACGATGTCCCCGGGTTGCAAGT<br>GTTTAAAGATGAGCACTGGTTTGATGCTAAGTACATCCCTAACGCGCTGATAGTCCACATTGGTGATC<br>AAGTTGAAATCATGAGTAATGGTAAGTACAAGTCTGTACTCCACCGGACGACCGTCAACAAGGAGAAG<br>GCCCGCATGTCGTGGCCCGTGTTCTGTTCACCCCCAGCAGAGCACGTCGTGGGCCCTCTGCCCCAG<br>TTAGTGAATGAAGAAAATCCATCCCGGTATAAGTACATTGAGTATAAGGACTACGTTCAGCTCAAACTG<br>AAGAACAAATCTGCGGCATTCGAAGGCCCTAACGTCGCGAAGGTTACTAGTAAATGA
McDAH$_{(14)}$	ATGGAGGTTGAAAAAGTACTGCAACCCATATCTAAATCAGGCGAAAGTGGCTATGATCATTTTATGCCA<br>GAAGACTTTATTCGTCCAGAGGATGAACAGCCGGAGCTGACCACCTTCAAAGGCCCGACACCGGATA<br>TTCCGGTGATCGATTTAAGTGAGCCAAATGAAGAAAAACTGGTGCGTGCCCTGGTGTCTGCATCGGAA<br>GAATGGGGTATCTTTCAGGTAGTGAACCATGGGATTCCAACTGATGTCATCGATAAGTTCATGCGGGT<br>AGGCCGTGAATTTTTTGAACTGCCGCAGGAAGAGAAAATGGCATACGCACGACCTCCAGGTGCAACC<br>TCCCTGGAAGGGTACGGGACTAAACTTCAGAAGGAGATCGAAGGTAAAAAGGCATGGGCAGACCTGC<br>TTTTTCATAATGTATGGCCTCCTTCCATTGTTAATTATTCTTTTTGGCCGAAGAACCCACCGTCATACAG<br>AGAAGCAACAGAAGAATATGCAAAACATATACCAATCGTAGCAGACAAGTTATTTAAAATTTTATCACT<br>GGGGTTGGGCCTCGAGGGTAATAGCATTAAGGAAGGTCTGGGTGGAGAAAAAATGGAGTTCCTTATG<br>AAGATTAATTACTATCCGCCGTGCCCAAGACCTGATCTTGCTTTAGGAGTTGTTCCACATACCGATATG<br>TCTGCAATCACGGTCCTCGTTCCGACCGATGTTCCTGGGTTACAAGTGTTTAAAGATGACCTCTGGTA<br>CGATGCTAAATATATCCCATACGCCCTCGTCGTTAATATAGCAGACCAGATTGAAATCTTATCCAACGG<br>TAAATATAAATCTGTTCTGCATAGAGCGAAAGTTAATAAAGAAAAAGTAAGAATGAGTTGGCCGATTTTT<br>TGTACCCCCCCTGCAGATATGGTAATCGGTCCGATCCCCGAATTAATTAATGAAGAAAATCCGAGTCG<br>CTATAAATATATAGAATATAAAGATTATGTGCAGCTGAAACTGAAAAACAAAAGCGCGGCCTTTGAAGG<br>CCCAAATGTGGCGAAAGTAACCAGTAAGTAA

Name	Sequence (5’ - 3’)
$McDAH_{(44)}$	ATGGAAGTTGAGAAAGTCCTTCAGCCAATATCGAAAAGTGGCGAAAGTGGCTACGACCACTTTATGCC<br>GGAAGACTTCATTAGACCCGAGGACGAGCAGCCTGAGCTGACAACATTCAAGGGCCCCACCCCCGAC<br>ATACCAGTAATAGATTTATCGGAACCGAATGAAGAAAAGCTTGTTCGCGCACTGGTCAGTGCCTCTGA<br>GGAATGGGGTATCTTTCAAGTTGTTAATCACGGTATCCCGACAGACGTCATAGACAAGTTCATGCGTG<br>TGGGTCGAGAGTTCTTCGAGCTTCCGCAGGAAGAGAAGATGGCCTATGCTCGCCCACCGGGTGCAAC<br>TAGTTTAGAGGGGTACGGTACCAAGCTTCAAAAGGAGATTGAAGGTAAGAAGGCTTGGGCGGATCTG<br>CTGTTTCATAATGTTTGGCCGCCGTCAATAGTTAACTACTCCTTCTGGCCGAAGAACCCACCGAGCTA<br>TCGTGAGGCCACTGAGGAGTACGCTAAGCACATACCGATTGTGGCCGATAAGTTATTCAAGATCTTAT<br>CTTTAGGCCTCGGGCTGGAAGGAAATAGTATAAAAGAGGGGCTGGGCGGCGAAAAGATGGAGTTCCT<br>CATGAAGATCAACTATTACCCACCGTGTCCAAGACCTGATTTAGCACTGGGTGTCGTTCCACATACCG<br>ACATGTCTGCTATCACCGTCCTCGTCCCGACGGACGTTCCAGGCCTGCAGGTTTTCAAGGACGACCT<br>GTGGTACGATGCCAAGTACATTCCCTATGCGCTGGTTGTTAACATCGCGGACCAAATAGAAATTCTGT<br>CAAATGGGAAGTACAAGAGTGTCTTGCACCGCGCGAAAGTTAATAAGGAAAAGGTTCGGATGTCATG<br>GCCAATCTTTTGTACCCCGCCGGCGGACATGGTGATCGGTCCCATACCCGAGCTTATCAATGAGGAA<br>AACCCTTCTCGGTACAAGTATATAGAATATAAGGATTACGTGCAGCTCAAGCTGTGA
AtF3H D219G	ATGGCTCCGGGTACTCTGACCGAACTTGCGGGTGAGTCTAAGCTGAATTCAAAATTTGTCCGGGATGA<br>AGACGAGCGCCCTAAGGTTGCGTATAACGTATTTTCGGATGAGATTCCAGTTATTTCACTCGCGGGTA<br>TTGACGACGTTGATGGTAAGCGTGGGGAAATTTGCCGCCAGATCGTTGAGGCATGTGAGAACTGGGG<br>CATTTTTCAGGTCGTGGACCACGGCGTCGATACGAATTTGGTGGCCGACATGACCCGCCTTGCTCGG<br>GACTTTTTTGCTCTGCCACCGGAGGATAAGCTGCGGTTTGATATGTCAGGGGGTAAAAAAGGGGGCT<br>TCATCGTGTCGTCGCACCTGCAAGGTGAAGCGGTCCAGGACTGGCGCGAGATTGTAACTTACTTCAG<br>TTACCCTGTGCGCAACCGCGACTACTCTCGGTGGCCAGACAAGCCTGAAGGTTGGGTCAAAGTAACT<br>GAGGAGTACTCCGAGCGGCTGATGAGTTTGGCGTGCAAATTGCTGGAGGTTTTGTCCGAGGCAATGG<br>GGTTGGAAAAGGAGTCACTCACTAATGCATGTGTGGATATGGATCAGAAGATCGTTGTGAATTATTAC<br>CCTAAATGTCCGCAACCAGATTTAACCCTTGGTCTTAAGCGCCATACCGGTCCTGGGACAATTACGTT<br>GCTGCTTCAAGATCAAGTGGGCGGTCTCCAAGCTACTCGTGATAACGGCAAAACCTGGATTACCGTC<br>CAGCCAGTCGAAGGTGCGTTTGTTGTTAATTTGGGGGATCATGGCCATTTTTTGTCAAATGGCCGCTT<br>CAAGAACGCTGACCACCAAGCTGTGGTAAATAGTAACAGCTCTCGTTTAAGCATCGCGACGTTCCAGA<br>ACCCTGCCCCGGACGCAACAGTTTACCCACTGAAGGTGCGTGAAGGTGAGAAGGCCATCTTGGAGGA<br>ACCTATCACTTTCGCTGAGATGTACAAGCGCAAAATGGGCCGCGACCTGGAATTAGCACGTCTCAAAA<br>AATTAGCGAAAGAGGAACGTGATCACAAAGAGGTAGACAAACCGGTCGACCAAATTTTCGCATAG
HnH6H D219A	ATGGCGACCTTTGTATCGAACTGGTCTACCAAAAGCGTGAGCGAGTCCTTTATTGCGCCTCTGCAAAA<br>ACGCGCCGAAAAAGACGTTCCGGTTGGTAACGATGTGCCAATTATCGACCTTCAACAGCATCACCATC<br>TGCTGGTACAACAGATTACGAAAGCCTGCCAGGACTTTGGTCTGTTCCAAGTTATTAACCACGGTTTC<br>CCGGAGGAGTTGATGCTCGAAACGATGGAAGTTTGTAAGGAGTTTTTCGCGCTGCCAGCAGAGGAGA<br>AGGAAAAATTTAAGCCGAAAGGCGAAGCGGCGAAGTTCGAGCTGCCGCTGGAACAGAAGGCTAAACT<br>GTACGTCGAGGGCGAACAATTATCCAACGAGGAGTTCCTGTACTGGAAGGACACTCTCGCGCATGGT<br>TGTCACCCGCTGGACCAGGACCTGGTGAACTCCTGGCCGGAGAAACCGGCTAAGTATAGAGAAGTCG<br>TGGCGAAGTATAGCGTTGAAGTCCGCAAGCTGACCATGCGTATGTTGGACTACATTTGCGAGGGCCT<br>GGGCTTGAAGTTGGGTTATTTTGATAACGAGCTGAGCCAGATTCAGATGATGTTGACCAATTATTACCC<br>GCCATGCCCGGACCCGTCTAGCACGTTGGGCAGCGGCGGTCACTACGCCGGTAACCTGATTACCCT<br>GCTGCAACAGGACTTGCCGGGTTTACAGCAACTGATTGTGAAGGACGCAACCTGGATCGCGGTCCAA<br>CCTATCCCGACGGCCTTCGTTGTTAATCTGGGTCTGACTTTGAAAGTCATCACCAATGAAAAGTTCGA<br>GGGCAGCATCCACCGTGTGGTGACCGATCCGACCCGTGATCGCGTGTCGATTGCAACCTTGATCGGT<br>CCAGATTACTCATGCACCATCGAACCGGCTAAGGAGCTGCTTAATCAAGACAATCCTCCGCTGTATAA<br>GCCGTATTCGTATAGCGAGTTCGCGGATATTTACCTGTCCGATAAGTCGGATTACGATTCCGGCGTTA<br>AACCGTACAAAATCAATGTG

Table S11.
FLS protonation states and charges (*q*) with neutral charges left blank.

Residue	*q*	Residue	*q*	Residue	*q*	Residue	*q*	Residue	*q*	Residue	*q*	Residue	*q*
MET1		THR50		PHE99		SER148		ASP197	-1	GLN246		MET295	
GLU2	-1	VAL51		PHE100		ARG149	+1	GLY198		VAL247		SER296	
VAL3		ASP52	-1	GLU101	-1	ILE150		VAL199		PHE248		TRP297	
ASP4	-1	LEU53		LEU102		ASN151		GLY200		LYS249	+1	PRO298	
TYR5		SER54		SER103		TYR152		GLY201		ASP250	-1	VAL299	
GLN6		GLU55	-1	GLN104		ARG153	+1	GLU202	-1	GLU251	-1	PHE300	
ARG7	+1	SER56		GLU105	-1	PHE154		SER203		HIP252	+1	CYS301	
VAL8		SER57		GLU106	-1	TRP155		MET204		TRP253		SER302	
GLN9		SER58		LYS107	+1	PRO156		GLU205	-1	PHE254		PRO303	
VAL10		ASP59	-1	GLU108	-1	LYS157	+1	TYR206		ASP255	-1	PRO304	
ILE11		GLU60	-1	ALA109		ASN158		LEU207		ALA256		ALA305	
ALA12		GLU61	-1	TYR110		PRO159		LEU208		LYS257	+1	GLU306	-1
THR13		SER62		ALA111		SER160		LYS209	+1	TYR258		HIE307	
LEU14		LEU63		ARG112	+1	TYR161		ILE210		ILE259		VAL308	
SER15		VAL64		VAL113		TYR162		ASN211		PRO260		VAL309	
LYS16	+1	ARG65	+1	SER114		ARG163	+1	TYR212		ASN261		GLY310	
ASN17		SER66		GLY115		GLU164	-1	TYR213		ALA262		PRO311	
THR18		ILE67		ASP116	-1	ALA165		PRO214		LEU263		LEU312	
THR19		ARG68	+1	SER117		ASN166		PRO215		ILE264		PRO313	
ASP20	-1	ASP69	-1	GLY118		GLU167	-1	CYS216		VAL265		GLN314	
ALA21		ALA70		LEU119		GLU168	-1	PRO217		HIE266		LEU315	
ASP22	-1	SER71		GLU120	-1	TYR169		ARG218	+1	ILE267		VAL316	
ASN23		ARG72	+1	GLY121		ALA170		PRO219		GLY268		ASN317	
ILE24		GLU73	-1	TYR122		LYS171	+1	ASP220	-1	ASP269	-1	GLU318	-1
PRO25		TRP74		GLY123		TRP172		LEU221		GLN270		GLU319	-1
GLU26	-1	GLY75		THR124		LEU173		ALA222		VAL271		ASN320	
GLU27	-1	PHE76		LYS125	+1	LYS174	+1	LEU223		GLU272	-1	PRO321	
PHE28		PHE77		LEU126		GLY175		GLY224		ILE273		PRO322	
ILE29		GLN78		GLN127		VAL176		VAL225		MET274		LYS323	+1
ARG30	+1	ILE79		LYS128	+1	VAL177		VAL226		SER275		TYR324	
PRO31		VAL80		GLU129	-1	GLU178	-1	PRO227		ASN276		LYS325	+1
GLU32	-1	ASN81		ILE130		LYS179	+1	HID228		GLY277		THR326	
ARG33	+1	HIE82		GLU131	-1	LEU180		THR229		LYS278	+1	LYS327	+1
GLU34	-1	GLY83		GLY132		MET181		ASP230	-1	TYR279		LYS328	+1
GLN35		ILE84		LYS133	+1	GLU182	-1	MET231		LYS280	+1	TYR329	
PRO36		PRO85		LYS134	+1	TRP183		SER232		SER281		LYS330	+1
ALA37		ILE86		ALA135		LEU184		ALA233		VAL282		ASP331	-1
LEU38		GLN87		TRP136		SER185		ILE234		LEU283		TYR332	
THR39		VAL88		VAL137		LEU186		THR235		HID284		GLU333	-1
THR40		ILE89		ASP138	-1	GLY187		VAL236		ARG285	+1	TYR334	
SER41		GLU90	-1	TYR139		LEU188		LEU237		THR286		CYS335	
ALA42		ARG91	+1	LEU140		GLY189		VAL238		THR287		LYS336	+1
GLY43		LEU92		PHE141		LEU190		PRO239		VAL288		LEU337	
PRO44		GLN93		HID142		GLU191	-1	ASN240		ASN289		ASN338	
VAL45		ARG94	+1	ASN143		LYS192	+1	ASP241	-1	LYS290	+1	LYS339	+1
PRO46		ALA95		VAL144		ASN193		VAL242		GLU291	-1	LEU340	
GLU47	-1	GLY96		TRP145		ALA194		PRO243		LYS292	+1	PRO341	
ILE48		LYS97	+1	PRO146		LEU195		GLY244		ALA293		GLN342	
PRO49		GLU98	-1	PRO147		ILE196		LEU245		ARG294	+1		

Table S12.
DAH protonation states and charges (*q*) with neutral charges left blank.

Residue	*q*	Residue	*q*	Residue	*q*	Residue	*q*	Residue	*q*	Residue	*q*	Residue	*q*
MET1		SER52		LYS103	+1	ASN154		LYS205	+1	PRO256		PRO307	
GLU2	-1	GLU53	-1	MET104		PRO155		ILE206		TYR257		ILE308	
VAL3		PRO54		ALA105		PRO156		ASN207		ALA258		PRO309	
GLU4	-1	ASP55	-1	TYR106		SER157		TYR208		LEU259		GLU310	-1
LYS5	+1	GLU56	-1	ALA107		TYR158		TYR209		VAL260		LEU311	
VAL6		GLU57	-1	ARG108	+1	ARG159	+1	PRO210		VAL261		ILE312	
LEU7		LYS58	+1	PRO109		GLU160	-1	PRO211		ASN262		ASN313	
GLN8		LEU59		PRO110		ALA161		CYS212		ILE263		GLU314	-1
PRO9		VAL60		GLY111		THR162		PRO213		ALA264		GLU315	-1
ILE10		ARG61	+1	ALA112		GLU163	-1	ARG214	+1	ASP265	-1	ASN316	
SER11		ALA62		THR113		GLU164	-1	PRO215		GLN266		PRO317	
LYS12	+1	LEU63		SER114		TYR165		ASP216	-1	ILE267		SER318	
SER13		VAL64		LEU115		ALA166		LEU217		GLU268	-1	ARG319	+1
GLY14		SER65		GLH116	+1	LYS167	+1	ALA218		ILE269		TYR320	
GLU15	-1	ALA66		GLY117		HIE168		LEU219		LEU270		LYS321	+1
SER16		SER67		TYR118		ILE169		GLY220		SER271		TYR322	
GLY17		GLU68	-1	GLU119	-1	PRO170		VAL221		ASN272		ILE323	
TYR18		GLU69	-1	THR120		ILE171		VAL222		GLY273		GLU324	-1
ASP19	-1	TRP70		ARG121	+1	VAL172		PRO223		LYS274	+1	TYR325	
HIE20		GLY71		PHE122		ALA173		HID224		TYR275		LYS326	+1
PHE21		ILE72		GLU123	-1	ASP174	-1	THR225		LYS276	+1	ASP327	-1
MET22		PHE73		ARG124	+1	LYS175	+1	GLY226		SER277		TYR328	
PRO23		GLN74		ASP125	-1	LEU176		TYR227		VAL278		VAL329	
GLU24	-1	VAL75		TYR126		PHE177		PRO228		LEU279		GLN330	
ASP25	-1	VAL76		VAL127		LYS178	+1	ALA229		HID280		LEU331	
PHE26		ASN77		GLY128		ILE179		MET230		ARG281	+1	LYS332	+1
ILE27		HIE78		LYS129	+1	LEU180		THR231		ALA282		LEU333	
ARG28	+1	GLY79		LYS130	+1	SER181		ILE232		LYS283	+1	LYS334	+1
PRO29		ILE80		ALA131		LEU182		LEU233		VAL284		ASN335	
GLU30	-1	PRO81		TRP132		GLY183		ILE234		ASN285		LYS336	+1
ASP31	-1	THR82		ALA133		LEU184		PRO235		LYS286	+1	SER337	
GLU32	-1	ASP83	-1	ASP134	-1	GLY185		THR236		GLU287	-1	ALA338	
GLN33		VAL84		LEU135		LEU186		ASP237	-1	LYS288	+1	ALA339	
PRO34		ILE85		LEU136		GLU187	-1	VAL238		VAL289		PHE340	
GLU35	-1	ASP86	-1	PHE137		GLY188		PRO239		ARG290	+1	GLU341	-1
LEU36		LYS87	+1	HIE138		ASN189		GLY240		MET291		GLY342	
THR37		PHE88		ASN139		THR190		LEU241		SER292		PRO343	
THR38		MET89		VAL140		ILE191		GLN242		TRP293		ASN344	
PHE39		ARG90	+1	TRP141		LYS192	+1	VAL243		PRO294		VAL345	
LYS40	+1	VAL91		PRO142		GLU193	-1	PHE244		ILE295		ALA346	
GLY41		GLY92		PRO143		GLY194		LYS245	+1	PHE296		LYS347	+1
PRO42		ARG93	+1	SER144		LEU195		ASP246	-1	CYS297		VAL348	
THR43		GLU94	-1	ILE145		GLY196		ASP247	-1	THR298		THR349	
PRO44		PHE95		VAL146		GLY197		LEU248		PRO299		SER350	
ASP45	-1	PHE96		ASN147		GLU198	-1	TRP249		PRO300		LYS351	+1
ILE46		GLU97	-1	TYR148		LYS199	+1	TYR250		ALA301			
PRO47		LEU98		SER149		MET200		ASP251	-1	ASP302	-1		
VAL48		PRO99		PHE150		GLU201	-1	ALA252		MET303			
ILE49		GLN100		TRP151		PHE202		LYS253	+1	VAL304			
ASP50	-1	GLU101	-1	PRO152		LEU203		TYR254		ILE305			
LEU51		GLU102	-1	LYS153	+1	MET204		ILE255		GLY306			

Table S13.

Flat-bottom harmonic restraints of FLS and DAH MD simulations. Flat-bottom harmonic restraints derived from experimentally motivated distance (Å) and angle (°) values were applied to each system. In all cases, the two restraints were the distance between the hydrogen atom target and the iron center (Fe H), and the angle between the hydrogen atom target, the iron center, and the oxo ligand (∠H-Fe-oxo). The flat bottom of the restraint spans r2 and r3, and the force is applied with a harmonic force constant between r1 and r2 as well as between r3 and r4. The restraint is centered on the experimental value with a width of the experimental uncertainty.

	Distances (Å)				Angles (°)			
**Simulation**	**r1**	**r2**	**r3**	**r4**	**r1**	**r2**	**r3**	**r4**
FLS acute	2.2	3.2	3.4	4.4	20	30	40	50
FLS obtuse	2.9	3.9	4.5	5.5	65	75	95	105
DAH acute	2.2	3.2	3.4	4.4	20	30	40	50
DAH obtuse	2.9	3.9	4.5	5.5	65	75	95	105

Table S14.

DBSCAN clustering statistics for all FLS MD simulations. The CPPTraj implementation was used and e was tuned incrementally until there were fewer than six clusters with the top three clusters shown. The clustering selection was chosen with the goal of identifying a representative frame for QM/MM. The centroid of the largest cluster was chosen. All other clusters were discarded. The clustering mask was selected to include the first and second coordination sphere, the substrate, the iron center, and residues in contact with the substrate. A large mask was used to identify a centroid that accurately captured the equilibrium conformation. The FLS mask was: L119, N127, F141, K209, H228, D230, S232, T235, H266, H284, R294, F300, S302, Y329, E333.

**FLS 2OG unrestrained**					
**#Cluster**	**Frames**	**Frac**	**AvgDist**	**Stdev**	**Centroid**
0	115228	0.922	0.975	0.155	33670
1	4077	0.033	0.833	0.169	903
**FLS succinate unrestrained**					
**#Cluster**	**Frames**	**Frac**	**AvgDist**	**Stdev**	**Centroid**
0	122172	0.977	1.127	0.222	94744
1	428	0.003	0.657	0.098	116362
**FLS succinate acute**					
**#Cluster**	**Frames**	**Frac**	**AvgDist**	**Stdev**	**Centroid**
0	117864	0.943	0.942	0.172	76269
1	476	0.004	0.615	0.075	6916
2	369	0.003	0.597	0.065	6392

Table S15.

DBSCAN clustering statistics for all DAH MD simulations. The CPPTraj implementation was used and e was tuned incrementally until there were fewer than six clusters with the top three shown. The clustering selection was chosen with the goal of identifying a representative frame for QM/MM. The centroid of the largest cluster was chosen. All other clusters were discarded. The clustering mask was selected to include the first and second coordination sphere, the substrate, the iron center, and residues in contact with the substrate. A large mask was used to identify a centroid that accurately captured the equilibrium conformation. The DAH mask was: L115, R121, L135, F137, K205, N207, H224, Y227, P228, T231, F296, H280, L333.

**DAH 2OG unrestrained**					
**#Cluster**	**Frames**	**Frac**	**AvgDist**	**Stdev**	**Centroid**
0	66508	0.532	1.273	0.322	61940
1	33415	0.267	0.989	0.233	29207
2	9578	0.077	0.960	0.175	115829
3	5693	0.046	1.077	0.202	109300
4	2180	0.017	0.936	0.172	2757
**DAH succinate eq-oxo unrestrained**					
**#Cluster**	**Frames**	**Frac**	**AvgDist**	**Stdev**	**Centroid**
0	75121	0.601	0.856	0.147	80588
1	19803	0.158	1.703	0.546	46050
2	18945	0.152	1.212	0.311	6133
**DAH succinate eq-oxo obtuse**					
**#Cluster**	**Frames**	**Frac**	**AvgDist**	**Stdev**	**Centroid**
0	63003	0.504	1.042	0.195	59067
1	44909	0.359	1.006	0.199	29882
2	5990	0.048	0.736	0.137	74059
3	3039	0.024	0.722	0.120	1335
4	530	0.004	0.662	0.087	104168
**DAH succinate ax-oxo unrestrained**					
**#Cluster**	**Frames**	**Frac**	**AvgDist**	**Stdev**	**Centroid**
0	107202	0.858	1.146	0.325	41101
1	550	0.004	0.703	0.096	105067
2	494	0.004	0.654	0.102	326
**DAH succinate ax-oxo obtuse**					
**#Cluster**	**Frames**	**Frac**	**AvgDist**	**Stdev**	**Centroid**
0	53171	0.425	0.782	0.123	114031
1	47607	0.381	1.101	0.342	66374
2	15578	0.125	0.952	0.233	14977

Table S16.
The residues in the QM region for the QM/MM FLS simulations with 2OG. The table contains the total number of atoms prior to adding link atoms along with their corresponding net charge.

Residue	# of atoms	Charge
Lys209	22	1
His228	17	0
Asp230	12	-1
His284	17	0
Fe	1	2
2-oxoglutarate (2OG)	14	-2
Dihydrokaempferol	33	0
**Total**	**116**	**0**

Table S17.

The residues in the QM region for the QM/MM FLS simulations with succinate. The table contains the total number of atoms prior to adding link atoms along with their corresponding net charge.

Residue	# of atoms	Charge
Lys209	22	1
His228	17	0
Asp230	12	-1
His284	17	0
Fe	1	4
Oxo	1	-2
Succinate	12	-2
Dihydrokaempferol	33	0
**Total**	**115**	**0**

Table S18.
The residues in the QM region for the QM/MM DAH simulations with 2OG. The table contains the total number of atoms prior to adding link atoms along with their corresponding net charge.

Residue	# of atoms	Charge
Lys205	22	1
His224	17	0
His280	16	0
Fe	1	2
Cl	1	-1
2-oxoglutarate (2OG)	14	-2
Dechloroacutumine	54	0
**Total**	**125**	**0**

Table S19.

The residues in the QM region for the QM/MM DAH simulations with succinate. The table contains the total number of atoms prior to adding link atoms along with their corresponding net charge.

Residue	# of atoms	Charge
Lys205	22	1
His224	17	0
His280	16	0
Fe	1	4
Oxo	1	-2
Cl	1	-1
Succinate	12	-2
Dechloroacutumine	54	0
**Total**	**124**	**0**

REFERENCES AND NOTES

1. V. Agarwal, Z. D. Miles, J. M. Winter, A. S. Eustáquio, A. A. El Gamal, B. S. Moore, Enzymatic halogenation and dehalogenation reactions: Pervasive and mechanistically diverse. *Chem. Rev.* **117**, 5619–5674 (2017).

2. F. H. Vaillancourt, E. Yeh, D. A. Vosburg, S. Garneau-Tsodikova, C. T. Walsh, Nature's inventory of halogenation catalysts: Oxidative strategies predominate. *Chem. Rev.* **106**, 3364–3378 (2006).

3. W.-J. Chung, C. D. Vanderwal, Stereoselective halogenation in natural product synthesis. *Angew. Chem. Int. Ed. Engl.* **55**, 4396–4434 (2016).

4. C. Y. Kim, A. J. Mitchell, C. M. Glinkerman, F.-S. Li, T. Pluskal, J.-K. Weng, The chloroalkaloid (−)-acutumine is biosynthesized via a Fe(II)- and 2-oxoglutarate-dependent halogenase in Menispermaceae plants. *Nat. Commun.* **11**, 1867 (2020).

5. J. M. Bollinger Jr., W.-C. Chang, M. L. Matthews, R. J. Martinie, A. K. Boal, C. Krebs, "Mechanisms of 2-oxoglutarate-dependent oxygenases: The hydroxylation paradigm and beyond" in *2-oxoglutarate-dependent oxygenases*" (Royal Society of Chemistry Cambridge, 2015), pp. 95–122.

6. S. Martinez, R. P. Hausinger, Catalytic mechanisms of Fe(II)- and 2-oxoglutarate-dependent oxygenases. *J. Biol. Chem.* **290**, 20702–20711 (2015).

7. A. J. Mitchell, J.-K. Weng, Unleashing the synthetic power of plant oxygenases: From mechanism to application. *Plant Physiol.* **179**, 813–829 (2019).

8. A. J. Mitchell, Q. Zhu, A. O. Maggiolo, N. R. Ananth, M. L. Hillwig, X. Liu, A. K. Boal, Structural basis for halogenation by iron- and 2-oxo-glutarate-dependent enzyme WelO5. *Nat. Chem. Biol.* **12**, 636–640 (2016).

9. M. E. Neugebauer, K. H. Sumida, J. G. Pelton, J. L. McMurry, J. A. Marchand, M. C. Y. Chang, A family of radical halogenases for the engineering of amino-acid-based products. *Nat. Chem. Biol.* **15**, 1009–1016 (2019).

10. C. A. Gomez, D. Mondal, Q. Du, N. Chan, J. C. Lewis, Directed evolution of an Iron(II)- and α-ketoglutarate-dependent dioxygenase for site-selective azidation of unactivated aliphatic C-H bonds. *Angew. Chem. Int. Ed. Engl.* **62**, e202301370 (2023).

11. A. J. Mitchell, N. P. Dunham, J. A. Bergman, B. Wang, Q. Zhu, W.-C. Chang, X. Liu, A. K. Boal, Structure-guided reprogramming of a hydroxylase to halogenate its small molecule substrate. *Biochemistry* **56**, 441–444 (2017).

12. A. Papadopoulou, F. Meyer, R. M. Buller, Engineering Fe(II)/α-ketoglutarate-dependent halogenases and desaturases. *Biochemistry* **62**, 229–240 (2023).

13. M. E. Neugebauer, E. N. Kissman, J. A. Marchand, J. G. Pelton, N. A. Sambold, D. C. Millar, M. C. Y. Chang, Reaction pathway engineering converts a radical hydroxylase into a halogenase. *Nat. Chem. Biol.* **18**, 171–179 (2022).

14. L. Chae, T. Kim, R. Nilo-Poyanco, S. Y. Rhee, Genomic signatures of specialized metabolism in plants. *Science* **344**, 510–513 (2014).

15. R. E. Kerwin, J. E. Hart, P. D. Fiesel, Y.-R. Lou, P. Fan, A. D. Jones, R. L. Last, Tomato root specialized metabolites evolved through gene duplication and regulatory divergence within a biosynthetic gene cluster. *Sci. Adv.* **10**, eadn3991 (2024).

16. M. Seppey, M. Manni, E. M. Zdobnov, BUSCO: Assessing genome assembly and annotation completeness. *Methods Mol. Biol.* **1962**, 227–245 (2019).

17. Y. Nevers, A. Warwick Vesztrocy, V. Rossier, C.-M. Train, A. Altenhoff, C. Dessimoz, N. M. Glover, Quality assessment of gene repertoire annotations with OMArk. *Nat. Biotechnol.* **43**, 124–133 (2025).

18. A. Rhie, B. P. Walenz, S. Koren, A. M. Phillippy, Merqury: Reference-free quality, completeness, and phasing assessment for genome assemblies. *Genome. Biol.* **21**, 245 (2020).

19. D. M. Emms, S. Kelly, OrthoFinder: Solving fundamental biases in whole genome comparisons dramatically improves orthogroup inference accuracy. *Genome Biol.* **16**, 157 (2015).

20. Z. Yang, PAML 4: Phylogenetic analysis by maximum likelihood. *Mol. Biol. Evol.* **24**, 1586–1591 (2007).

21. M. S. Barker, N. C. Kane, M. Matvienko, A. Kozik, R. W. Michelmore, S. J. Knapp, L. H. Rieseberg, Multiple paleopolyploidizations during the evolution of the compositae reveal parallel patterns of duplicate gene retention after millions of years. *Mol. Biol. Evol.* **25**, 2445–2455 (2008).

22. M. S. Barker, K. M. Dlugosch, L. Dinh, R. S. Challa, N. C. Kane, M. G. King, L. H. Rieseberg, EvoPipes.net: Bioinformatic tools for ecological and evolutionary genomics. *Evol. Bioinform. Online* **6**, 143–149 (2010).

23. G. P. Tiley, M. S. Barker, J. G. Burleigh, Assessing the performance of Ks plots for detecting ancient whole genome duplications. *Genome Biol. Evol.* **10**, 2882–2898 (2018).

24. T. Benaglia, *Mixtools: An R Package for Analyzing Finite Mixture Models* (2009).

25. Amborella Genome Project, The Amborella genome and the evolution of flowering plants. *Science* **342**, 1241089 (2013).

26. X. Yang, S. Gao, L. Guo, B. Wang, Y. Jia, J. Zhou, Y. Che, P. Jia, J. Lin, T. Xu, J. Sun, K. Ye, Three chromosome-scale Papaver genomes reveal punctuated patchwork evolution of the morphinan and noscapine biosynthesis pathway. *Nat. Commun.* **12**, 6030 (2021).

27. L. Leng, Z. Xu, B. Hong, B. Zhao, Y. Tian, C. Wang, L. Yang, Z. Zou, L. Li, K. Liu, W. Peng, J. Liu, Z. An, Y. Wang, B. Duan, Z. Hu, C. Zheng, S. Zhang, X. Li, M. Li, Z. Liu, Z. Bi, T. He, B. Liu, H. Fan, C. Song, Y. Tong, S. Chen, Cepharanthine analogs mining and genomes of Stephania accelerate anti-coronavirus drug discovery. *Nat. Commun.* **15**, 1537 (2024).

28. L. Glick, S. Castiglione, G. Loewenthal, P. Raia, T. Pupko, I. Mayrose, Phylogenetic analysis of 590 species reveals distinct evolutionary patterns of intron-exon gene structures across eukaryotic lineages. *Mol. Biol. Evol.* **41**, msae248 (2024).

29. M. Chorev, L. Carmel, The function of introns. *Front. Genet.* **3**, 55 (2012).

30. J. Jumper, R. Evans, A. Pritzel, T. Green, M. Figurnov, O. Ronneberger, K. Tunyasuvunakool, R. Bates, A. Žídek, A. Potapenko, A. Bridgland, C. Meyer, S. A. A. Kohl, A. J. Ballard, A. Cowie, B. Romera-Paredes, S. Nikolov, R. Jain, J. Adler, T. Back, S. Petersen, D. Reiman, E. Clancy, M. Zielinski, M. Steinegger, M. Pacholska, T. Berghammer, S. Bodenstein, D. Silver, O. Vinyals, A. W. Senior, K. Kavukcuoglu, P. Kohli, D. Hassabis, Highly accurate protein structure prediction with AlphaFold. *Nature* **596**, 583–589 (2021).

31. R. W. D. Welford, I. J. Clifton, J. J. Turnbull, S. C. Wilson, C. J. Schofield, Structural and mechanistic studies on anthocyanidin synthase catalysed oxidation of flavanone substrates: The effect of C-2 stereochemistry on product selectivity and mechanism. *Org. Biomol. Chem.* **3**, 3117–3126 (2005).

32. D. W. Kastner, A. Nandy, R. Mehmood, H. J. Kulik, Mechanistic insights into substrate positioning that distinguish non-heme Fe(II)/α-ketoglutarate-dependent halogenases and hydroxylases. *ACS Catal.* **13**, 2489–2501 (2023).

33. R. De Smet, K. L. Adams, K. Vandepoele, M. C. E. Van Montagu, S. Maere, Y. Van de Peer, Convergent gene loss following gene and genome duplications creates single-copy families in flowering plants. *Proc. Natl. Acad. Sci. U.S.A.* **110**, 2898–2903 (2013).

34. F. He, A. Jacobson, Nonsense-mediated mRNA decay: Degradation of defective transcripts is only part of the story. *Annu. Rev. Genet.* **49**, 339–366 (2015).

35. G. Amitai, R. D. Gupta, D. S. Tawfik, Latent evolutionary potentials under the neutral mutational drift of an enzyme. *HFSP J.* **1**, 67–78 (2007).

36. D. M. Weinreich, N. F. Delaney, M. A. Depristo, D. L. Hartl, Darwinian evolution can follow only very few mutational paths to fitter proteins. *Science* **312**, 111–114 (2006).

37. R. Mehmood, V. Vennelakanti, H. J. Kulik, Spectroscopically guided simulations reveal distinct strategies for positioning substrates to achieve selectivity in nonheme Fe(II)/α-ketoglutarate-dependent halogenases. *ACS Catal.* **11**, 12394–12408 (2021).

38. R. J. Martinie, J. Livada, W.-C. Chang, M. T. Green, C. Krebs, J. M. Bollinger Jr., A. Silakov, Experimental correlation of substrate position with reaction outcome in the aliphatic halogenase, SyrB2. *J. Am. Chem. Soc.* **137**, 6912–6919 (2015).

39. J. W. Slater, C.-Y. Lin, M. E. Neugebauer, M. J. McBride, D. Sil, M. A. Nair, B. J. Katch, A. K. Boal, M. C. Y. Chang, A. Silakov, C. Krebs, J. M. Bollinger Jr., Synergistic binding of the halide and cationic prime substrate of l-lysine 4-chlorinase, BesD, in both ferrous and ferryl states. *Biochemistry* **62**, 2480–2491 (2023).

40. E. R. Smithwick, R. H. Wilson, S. Chatterjee, Y. Pu, J. J. Dalluge, A. R. Damodaran, A. Bhagi-Damodaran, Electrostatically regulated active site assembly governs reactivity in non-heme iron halogenases. bioRxiv 542349 [Preprint] (2023). https://doi:10.1101/2023.05.25.542349.

41. W. Wang, S. Song, N. Jiao, Late-stage halogenation of complex substrates with readily available halogenating reagents. *Acc. Chem. Res.* **57**, 3161–3181 (2024).

42. B. R. Smith, C. M. Eastman, J. T. Njardarson, Beyond C, H, O, and N! Analysis of the elemental composition of U.S. FDA approved drug architectures. *J. Med. Chem.* **57**, 9764–9773 (2014).

43. J. Dauparas, I. Anishchenko, N. Bennett, H. Bai, R. J. Ragotte, L. F. Milles, B. I. M. Wicky, A. Courbet, R. J. de Haas, N. Bethel, P. J. Y. Leung, T. F. Huddy, S. Pellock, D. Tischer, F. Chan, B. Koepnick, H. Nguyen, A. Kang, B. Sankaran, A. K. Bera, N. P. King, D. Baker, Robust deep learning-based protein sequence design using ProteinMPNN. *Science* **378**, 49–56 (2022).

44. J. A. Ruffolo, A. Bhatnagar, J. Beazer, S. Nayfach, J. Russ, E. Hill, R. Hussain, J. Gallagher, A. Madani, Adapting protein language models for structure-conditioned design. bioRxiv 606485 [Preprint] (2024). https://doi.org/10.1101/2024.08.03.606485.

45. S. Chen, Y. Zhou, Y. Chen, J. Gu, fastp: An ultra-fast all-in-one FASTQ preprocessor. *Bioinformatics* **34**, i884–i890 (2018).

46. G. Marçais, C. Kingsford, A fast, lock-free approach for efficient parallel counting of occurrences of k-mers. *Bioinformatics* **27**, 764–770 (2011).

47. T. R. Ranallo-Benavidez, K. S. Jaron, M. C. Schatz, GenomeScope 2.0 and Smudgeplot for reference-free profiling of polyploid genomes. *Nat. Commun.* **11**, 1432 (2020).

48. H. Cheng, G. T. Concepcion, X. Feng, H. Zhang, H. Li, Haplotype-resolved de novo assembly using phased assembly graphs with hifiasm. *Nat. Methods* **18**, 170–175 (2021).

49. N. C. Durand, M. S. Shamim, I. Machol, S. S. P. Rao, M. H. Huntley, E. S. Lander, E. L. Aiden, Juicer provides a one-click system for analyzing loop-resolution Hi-C experiments. *Cell systems* **3**, 95–98 (2016).

50. O. Dudchenko, S. S. Batra, A. D. Omer, S. K. Nyquist, M. Hoeger, N. C. Durand, M. S. Shamim, I. Machol, E. S. Lander, A. P. Aiden, E. L. Aiden, De novo assembly of the *Aedes aegypti* genome using Hi-C yields chromosome-length scaffolds. *Science* **356**, 92–95 (2017).

51. N. C. Durand, J. T. Robinson, M. S. Shamim, I. Machol, J. P. Mesirov, E. S. Lander, E. L. Aiden, Juicebox provides a visualization system for Hi-C contact maps with unlimited zoom. *Cell Syst.* **3**, 99–101 (2016).

52. A. Mikheenko, A. Prjibelski, V. Saveliev, D. Antipov, A. Gurevich, Versatile genome assembly evaluation with QUAST-LG. *Bioinformatics* **34**, i142–i150 (2018).

53. L. S. Johnson, S. R. Eddy, E. Portugaly, Hidden Markov model speed heuristic and iterative HMM search procedure. *BMC Bioinformatics* **11**, 431 (2010).

54. E. Levy Karin, M. Mirdita, J. Söding, MetaEuk-sensitive, high-throughput gene discovery, and annotation for large-scale eukaryotic metagenomics. *Microbiome* **8**, 48 (2020).

55. Website. J. Palmer, J.Stajich, nextgenusfs/funannotate: funannotate v1.5.3. (2019); https://doi.org/10.5281/zenodo.2604804.

56. W. Li, L. Jaroszewski, A. Godzik, Tolerating some redundancy significantly speeds up clustering of large protein databases. *Bioinformatics* **18**, 77–82 (2002).

57. D. M. Emms, S. Kelly, OrthoFinder: Phylogenetic orthology inference for comparative genomics. *Genome Biol.* **20**, 238 (2019).

58. K. Katoh, D. M. Standley, MAFFT multiple sequence alignment software version 7: Improvements in performance and usability. *Mol. Biol. Evol.* **30**, 772–780 (2013).

59. M. Suyama, D. Torrents, P. Bork, PAL2NAL: Robust conversion of protein sequence alignments into the corresponding codon alignments. *Nucleic Acids Res.* **34**, W609–W612 (2006).

60. S. Capella-Gutiérrez, J. M. Silla-Martínez, T. Gabaldón, trimAl: A tool for automated alignment trimming in large-scale phylogenetic analyses. *Bioinformatics* **25**, 1972–1973 (2009).

61. A. Stamatakis, RAxML version 8: A tool for phylogenetic analysis and post-analysis of large phylogenies. *Bioinformatics* **30**, 1312–1313 (2014).

62. C. Zhang, M. Rabiee, E. Sayyari, S. Mirarab, ASTRAL-III: Polynomial time species tree reconstruction from partially resolved gene trees. *BMC Bioinformatics* **19**, 153 (2018).

63. Y. Liu, B. Wang, S. Shu, Z. Li, C. Song, D. Liu, Y. Niu, J. Liu, J. Zhang, H. Liu, Z. Hu, B. Huang, X. Liu, W. Liu, L. Jiang, M. M. Alami, Y. Zhou, Y. Ma, X. He, Y. Yang, T. Zhang, H. Hu, M. S. Barker, S. Chen, X. Wang, J. Nie, Analysis of the *Coptis chinensis* genome reveals the diversification of protoberberine-type alkaloids. *Nat. Commun.* **12**, 3276 (2021).

64. S. Kumar, M. Suleski, J. M. Craig, A. E. Kasprowicz, M. Sanderford, M. Li, G. Stecher, S. B. Hedges, TimeTree 5: An expanded resource for species divergence times. *Mol. Biol. Evol.* **39**, msac174 (2022).

65. F. K. Mendes, D. Vanderpool, B. Fulton, M. W. Hahn, CAFE 5 models variation in evolutionary rates among gene families. *Bioinformatics* **36**, 5516–5518 (2021).

66. M. D. Smith, J. O. Wertheim, S. Weaver, B. Murrell, K. Scheffler, S. L. Kosakovsky Pond, Less is more: An adaptive branch-site random effects model for efficient detection of episodic diversifying selection. *Mol. Biol. Evol.* **32**, 1342–1353 (2015).

67. S. L. K. Pond, S. D. W. Frost, S. V. Muse, HyPhy: Hypothesis testing using phylogenies. *Bioinformatics* **21**, 676–679 (2005).

68. R. C. Edgar, MUSCLE: Multiple sequence alignment with high accuracy and high throughput. *Nucleic Acids Res.* **32**, 1792–1797 (2004).

69. S. Kumar, G. Stecher, M. Li, C. Knyaz, K. Tamura, MEGA X: Molecular evolutionary genetics analysis across computing platforms. *Mol. Biol. Evol.* **35**, 1547–1549 (2018).

70. Y. Wang, H. Tang, X. Wang, Y. Sun, P. V. Joseph, A. H. Paterson, Detection of colinear blocks and synteny and evolutionary analyses based on utilization of MCScanX. *Nat. Protoc.* **19**, 2206–2229 (2024).

71. J. Pan, E. S. Wenger, M. L. Matthews, C. J. Pollock, M. Bhardwaj, A. J. Kim, B. D. Allen, R. B. Grossman, C. Krebs, J. M. Bollinger Jr., Evidence for modulation of oxygen rebound rate in control of outcome by iron(II)- and 2-oxoglutarate-dependent oxygenases. *J. Am. Chem. Soc.* **141**, 15153–15165 (2019).

72. R. Anandakrishnan, B. Aguilar, A. V. Onufriev, H++ 3.0: Automating pK prediction and the preparation of biomolecular structures for atomistic molecular modeling and simulations. *Nucleic Acids Res.* **40**, W537–W541 (2012).

73. J. C. Gordon, J. B. Myers, T. Folta, V. Shoja, L. S. Heath, A. Onufriev, H++: A server for estimating pKas and adding missing hydrogens to macromolecules. *Nucleic Acids Res.* **33**, W368–W371 (2005).

74. J. Myers, G. Grothaus, S. Narayanan, A. Onufriev, A simple clustering algorithm can be accurate enough for use in calculations of pKs in macromolecules. *Proteins* **63**, 928–938 (2006).

75. J. Eberhardt, D. Santos-Martins, A. F. Tillack, S. Forli, AutoDock Vina 1.2.0: New docking methods, expanded force field, and Python bindings. *J. Chem. Inf. Model.* **61**, 3891–3898 (2021).

76. O. Trott, A. J. Olson, AutoDock Vina: Improving the speed and accuracy of docking with a new scoring function, efficient optimization, and multithreading. *J. Comput. Chem.* **31**, 455–461 (2010).

77. J. A. Maier, C. Martinez, K. Kasavajhala, L. Wickstrom, K. E. Hauser, C. Simmerling, ff14SB: Improving the accuracy of protein side chain and backbone parameters from ff99SB. *J. Chem. Theory Comput.* **11**, 3696–3713 (2015).

78. C. I. Bayly, P. Cieplak, W. Cornell, P. A. Kollman, A well-behaved electrostatic potential based method using charge restraints for deriving atomic charges: The RESP model. *J. Phys. Chem.* **97**, 10269–10280 (1993).

79. P. C. Hariharan, J. A. People, The influence of polarization functions on molecular orbital hydrogenation energies. *Theor. Chem. Acc.* **28**, 213–222 (1973).

80. P. Li, K. M. Merz Jr., MCPB.py: A Python based metal center parameter builder. *J. Chem. Inf. Model.* **56**, 599–604 (2016).

81. C. Lee, W. Yang, R. G. Parr, Development of the Colle-Salvetti correlation-energy formula into a functional of the electron density. *Phys. Rev. B Condens. Matter* **37**, 785–789 (1988).

82. P. J. Stephens, F. J. Devlin, C. F. Chabalowski, M. J. Frisch, Ab-Initio calculation of vibrational absorption and circular-dichroism spectra using density-functional force-fields. *J. Phys. Chem.* **98**, 11623–11627 (1994).

83. J. M. Seminario, Calculation of intramolecular force fields from second-derivative tensors. *Int. J. Quantum Chem.* **60**, 1271–1277 (1996).

84. W. L. Jorgensen, J. Chandrasekhar, J. D. Madura, R. W. Impey, M. L. Klein, Comparison of simple potential functions for simulating liquid water. *J. Chem. Phys.* **79**, 926–935 (1983).

85. T. Darden, D. York, L. Pedersen, Particle mesh Ewald: An $N \cdot \log(N)$ method for Ewald sums in large systems. *J. Chem. Phys.* **98**, 10089–10092 (1993).

86. A. W. Götz, M. J. Williamson, D. Xu, D. Poole, S. Le Grand, R. C. Walker, Routine microsecond molecular dynamics simulations with AMBER on GPUs. 1. Generalized Born. *J. Chem. Theory Comput.* **8**, 1542–1555 (2012).

87. R. Salomon-Ferrer, A. W. Götz, D. Poole, S. Le Grand, R. C. Walker, Routine microsecond molecular dynamics simulations with AMBER on GPUs. 2. Explicit eolvent particle mesh Ewald. *J. Chem. Theory Comput.* **9**, 3878–3888 (2013).

88. J.-P. Ryckaert, G. Ciccotti, H. J. C. Berendsen, Numerical integration of the cartesian equations of motion of a system with constraints: Molecular dynamics of n-alkanes. *J. Comput. Phys.* **23**, 327–341 (1977).

89. T. M. Casey, P. K. Grzyska, R. P. Hausinger, J. McCracken, Measuring the orientation of taurine in the active site of the non-heme Fe(II)/α-ketoglutarate-dependent taurine hydroxylase (TauD) using electron spin echo envelope modulation (ESEEM) spectroscopy. *J. Phys. Chem. B* **117**, 10384–10394 (2013).

90. A. J. Mitchell, N. P. Dunham, R. J. Martinie, J. A. Bergman, C. J. Pollock, K. Hu, B. D. Allen, W.-C. Chang, A. Silakov, J. M. Bollinger Jr., C. Krebs, A. K. Boal, Visualizing the reaction cycle in an iron(II)- and 2-(oxo)-glutarate-dependent hydroxylase. *J. Am. Chem. Soc.* **139**, 13830–13836 (2017).

91. R. Mehmood, H. W. Qi, A. H. Steeves, H. J. Kulik, The protein's role in substrate positioning and reactivity for biosynthetic enzyme complexes: The case of SyrB2/SyrB1. *ACS Catal.* **9**, 4930–4943 (2019).

92. J. J. Shane, P. Höfer, E. J. Reijerse, E. de Boer, Hyperfine sublevel correlation spectroscopy (HYSCORE) of disordered solids. *J. Magn. Reson.* **99**, 596–604 (1992).

93. M. L. Matthews, C. S. Neumann, L. A. Miles, T. L. Grove, S. J. Booker, C. Krebs, C. T. Walsh, J. M. Bollinger Jr., Substrate positioning controls the partition between halogenation and hydroxylation in the aliphatic halogenase, SyrB2. *Proc. Natl. Acad. Sci. U.S.A.* **106**, 17723–17728 (2009).

94. D. R. Roe, T. E. Cheatham III, PTRAJ and CPPTRAJ: Software for processing and analysis of molecular dynamics trajectory data. *J. Chem. Theory Comput.* **9**, 3084–3095 (2013).

95. B. R. Miller III, T. D. McGee Jr., J. M. Swails, N. Homeyer, H. Gohlke, A. E. Roitberg, MMPBSA.py: An efficient program for end-state free energy calculations. *J. Chem. Theory Comput.* **8**, 3314–3321 (2012).

96. I. Massova, P. A. Kollman, Combined molecular mechanical and continuum solvent approach (MM-PBSA/GBSA) to predict ligand binding. *Perspect. Drug Discov. Des.* **18**, 113–135 (2000).

97. A. Onufriev, D. Bashford, D. A. Case, Exploring protein native states and large-scale conformational changes with a modified generalized born model. *Proteins* **55**, 383–394 (2004).

98. T. Hou, J. Wang, Y. Li, W. Wang, Assessing the performance of the MM/PBSA and MM/GBSA methods. 1. The accuracy of binding free energy calculations based on molecular dynamics simulations. *J. Chem. Inf. Model.* **51**, 69–82 (2011).

99. L. L. C. Schrodinger, The PyMOL molecular graphics system. in version 1.8 (2015).

100. S. Seritan, C. Bannwarth, B. S. Fales, E. G. Hohenstein, C. M. Isborn, S. I. L. Kokkila-Schumacher, X. Li, F. Liu, N. Luehr, J. W. Snyder, C. C. Song, V. Titov, I. S. Ufimtsev, L. P. Wang, T. J. Martinez, TeraChem: Graphical processing unit-acceleratedelectronic structure package forlarge-scaleab initio molecular dynamics. *WIREs Comput. Mol. Sci.* **11**, e1494 (2020).

101. H. J. Kulik, N. Luehr, I. S. Ufimtsev, T. J. Martinez, Ab initio quantum chemistry for protein structures. *J. Phys. Chem. B* **116**, 12501–12509 (2012).

102. I. S. Ufimtsev, T. J. Martínez, Quantum chemistry on graphical processing units. 1. Strategies for two-electron integral evaluation. *J. Chem. Theory Comput.* **4**, 222–231 (2008).

103. M. A. Rohrdanz, K. M. Martins, J. M. Herbert, A long-range-corrected density functional that performs well for both ground-state properties and time-dependent density functional theory excitation energies, including charge-transfer excited states. *J. Chem. Phys.* **130**, 054112 (2009).

104. P. J. Hay, W. R. Wadt, Ab initio effective core potentials for molecular calculations. Potentials for the transition metal atoms Sc to Hg. *J. Chem. Phys.* **82**, 270–283 (1985).

105. P. M. Rice, Peter M. Rice, Alan J. Bleasby, Jon C. Ison, A. J. Bleasby, J. C. Ison, *EMBOSS User's Guide: Practical Bioinformatics with EMBOSS* (Cambridge Univ. Press).

106. Tomato Genome Consortium, The tomato genome sequence provides insights into fleshy fruit evolution. *Nature* **485**, 635–641 (2012).

107. L. Guo, T. Winzer, X. Yang, Y. Li, Z. Ning, Z. He, R. Teodor, Y. Lu, T. A. Bowser, I. A. Graham, K. Ye, The opium poppy genome and morphinan production. *Science* **362**, 343–347 (2018).

108. K. M. Davis, M. Altmyer, R. J. Martinie, I. Schaperdoth, C. Krebs, J. M. Bollinger Jr., A. K. Boal, Structure of a ferryl mimic in the archetypal Iron(II)- and 2-(Oxo)-glutarate-dependent dioxygenase, TauD. *Biochemistry* **58**, 4218–4223 (2019).

109. A. Onufriev, D. Bashford, D. A. Case, Modification of the generalized born model suitable for macromolecules. *J. Phys. Chem. B* **104**, 3712–3720 (2000).

110. H. Li, Toward better understanding of artifacts in variant calling from high-coverage samples. *Bioinformatics* **30**, 2843–2851 (2014).

111. H. Li, Minimap and miniasm: Fast mapping and de novo assembly for noisy long sequences. *Bioinformatics* **32**, 2103–2110 (2016).

112. Z. An, R. Gao, S. Chen, Y. Tian, Q. Li, L. Tian, W. Zhang, L. Kong, B. Zheng, L. Hao, T. Xin, H. Yao, Y. Wang, W. Song, X. Hua, C. Liu, J. Song, H. Fan, W. Sun, S. Chen, Z. Xu, Lineage-specific CYP80 expansion and benzylisoquinoline alkaloid diversity in early-siverging eudicots. *Adv. Sci.* **11**, e2309990 (2024).
